# Author Correction: A therapeutic regimen using neoantigen-specific TCR-T cells for HLA-A*2402-positive solid tumors

**DOI:** 10.1038/s44321-026-00487-5

**Published:** 2026-07-16

**Authors:** Yuncheng Bei, Ying Huang, Nandie Wu, Yishan Li, Ruihan Xu, Baorui Liu, Rutian Li

**Affiliations:** 1 The Comprehensive Cancer Center, Nanjing Drum Tower Hospital, The Affiliated Hospital of Nanjing University Medical School, 210008 Nanjing, China; 2 Department of Oncology, The Affiliated Huai’an Hospital of Xuzhou Medical University and The Second People’s Hospital of Huai’an, 223022 Huai’an, China; 3 Clinical Cancer Institute of Nanjing University, 210008 Nanjing, China

## Abstract

**Figure:**
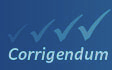

**Correction to:** *EMBO Molecular Medicine* (2025) 17, 365–383. 10.1038/s44321-024-00184-1 | Published online 02 January 2025

The authors contacted the journal to inform them of a labelling error in Fig. 4C. After reviewing the corrected figure provided by the authors, the journal agrees to retract and replace Fig. 4C.

**Figure 4C is retracted and replaced**.

**Original Figure**

**Figure:**
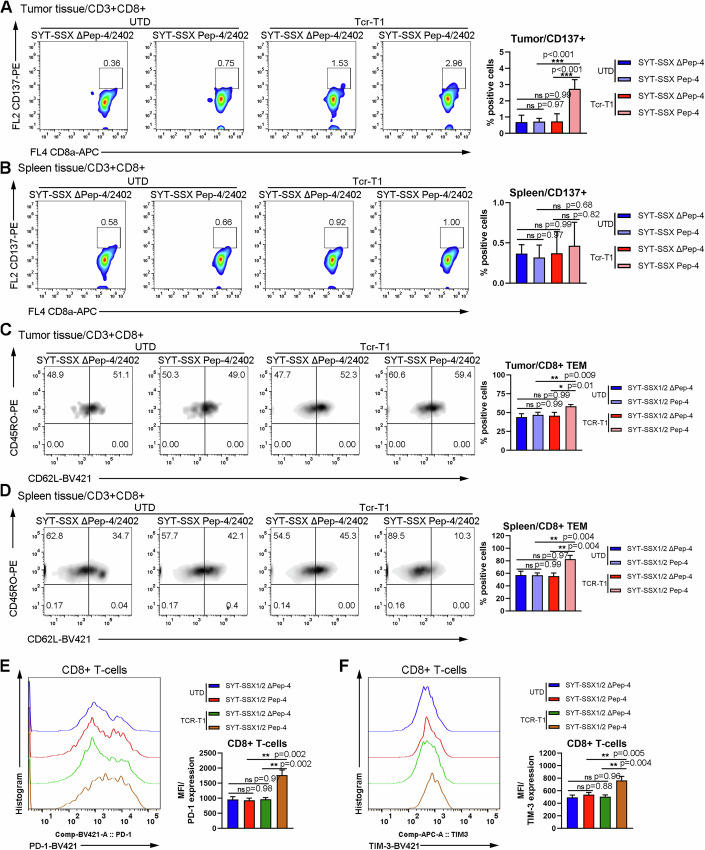

**Corrected Figure**

**Figure:**
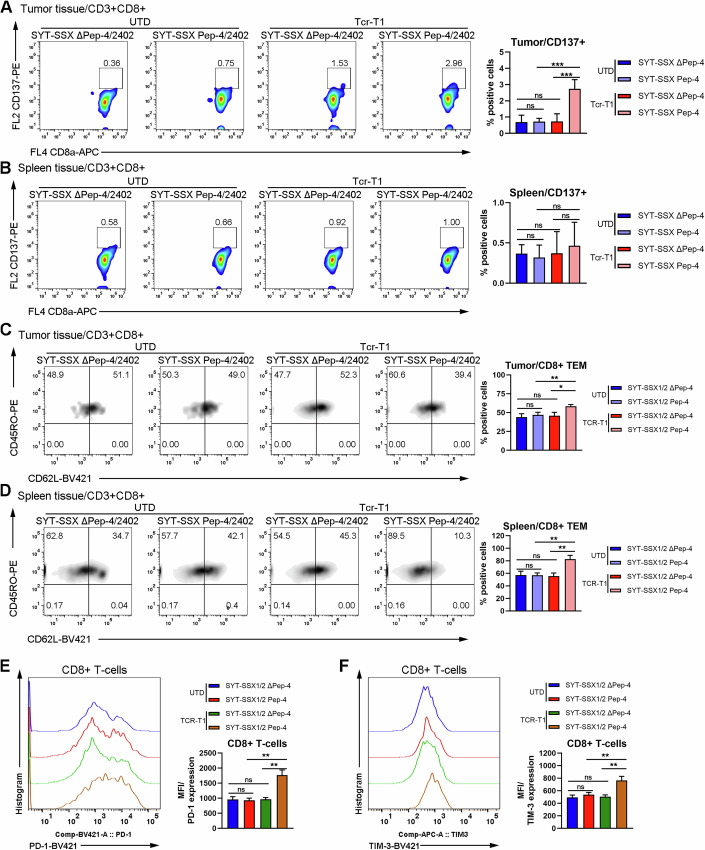

Author statement:

The percentage in the upper-right quadrant of the flow cytometry plot should read “39.4” but was mistakenly labeled “59.4.” This has now been corrected.

This correction does not affect the conclusions of the study. All authors agree to this correction.

