# Supplementary material for: A therapeutic regimen using neoantigen-specific TCR-T cells for HLA-A*2402-positive solid tumors
Source: EMBO Mol Med. 2025 Jan 2;17(2):365–83. doi: 10.1038/s44321-024-00184-1 (PMC11821884; doi:10.1038/s44321-024-00184-1)
Supplement: Supplementary file 1 — Appendix [file 44321_2024_184_MOESM1_ESM.pdf]

Appendix

Appendix Figure S1.	Identification of SYT-SSX fusion mutation.
Appendix Figure S2.	Identification of immunogenicity of potential neo-peptides.
Appendix Figure S3.	ScRNA-seq and scTCR-seq revealed Pep-4-specific TCR repertoire
Appendix Figure S4.	Tcr-T1 cells exhibited antigen-specific cytotoxicity against HLA-A*2402-positive synovial sarcoma cells
Appendix Figure S5.	Examination of toxicity of transfer of Tcr-T1 cells
Appendix Figure S6.	Flow cytometric analysis of T-cell activation in tumor and spleen tissues.
Appendix Figure S7.	Flow cytometric analysis of memory T-cell subsets and exhaustion.
Appendix Table S1.	Top 7 Clonotype CDR3 Sequences
Appendix Table S2.	AA sequences of synthetic TCR
Appendix Table S3	Properties of Pep-4 NP

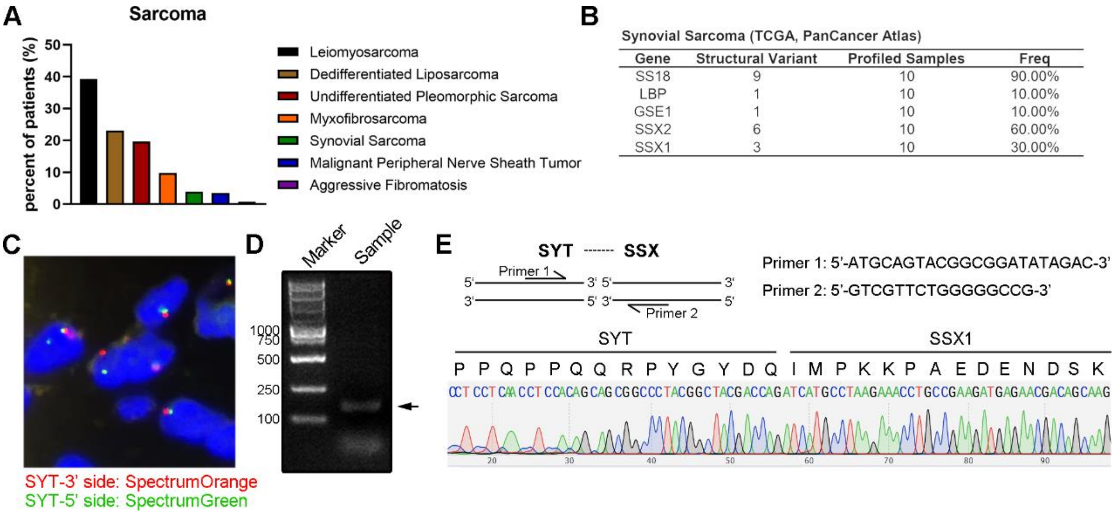

Appendix Figure S1. Identification of SYT-SSX fusion mutation.

(A) Incidence of sarcoma at cBioPortal website. **(B)** Frequency of gene fusion mutation in synovial sarcoma at cBioPortal website. **(C)** The FISH assay detected SYT-SSX fusion mutation in clinical specimens from patients with synovial sarcoma. **(D-E)** Identification of SYT-SSX fusion mutation via Sanger sequencing. DNA fragment (D) of SYT-SSX fusion mutation was amplified by using primer 1, and primer 2 (E, top), and consequently subjected to Sanger sequencing (E, bottom).

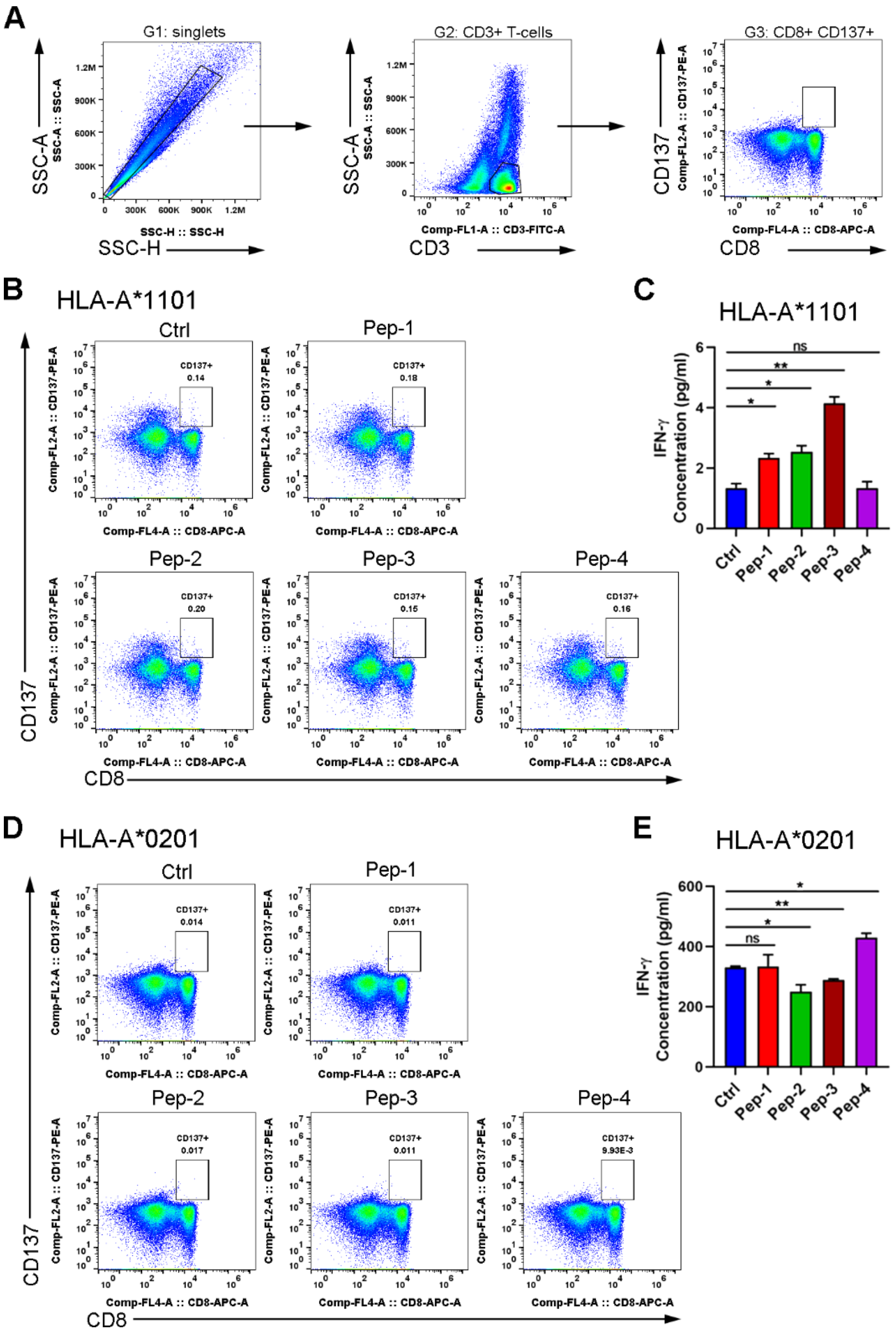

Appendix Figure S2. Identification of immunogenicity of potential neo-peptides.

(A-E) The immunogenicity of candidate neo-peptides, predicted via online NetMHC-pan4.0, was examined by using an optimized two-step culture protocol for fast in-vitro expansion of NRT cells as described in methods. Flow cytometric image exhibited gating strategy (A). Representative flow cytometric analysis of CD137 expression in PBMC derived from HLA-A*1101 (B), and HLA-A*0201 (D) healthy donors after NRT stimulation. IFN-γ production was parallelly determined via CBA assay (C, and E). Data are presented as mean ± SEM. Statistical significance was calculated using the one-way ANOVA (C, E). ns, not significant, $p < 0.05$, *; $p < 0.01$ **; $p < 0.001$, ***.

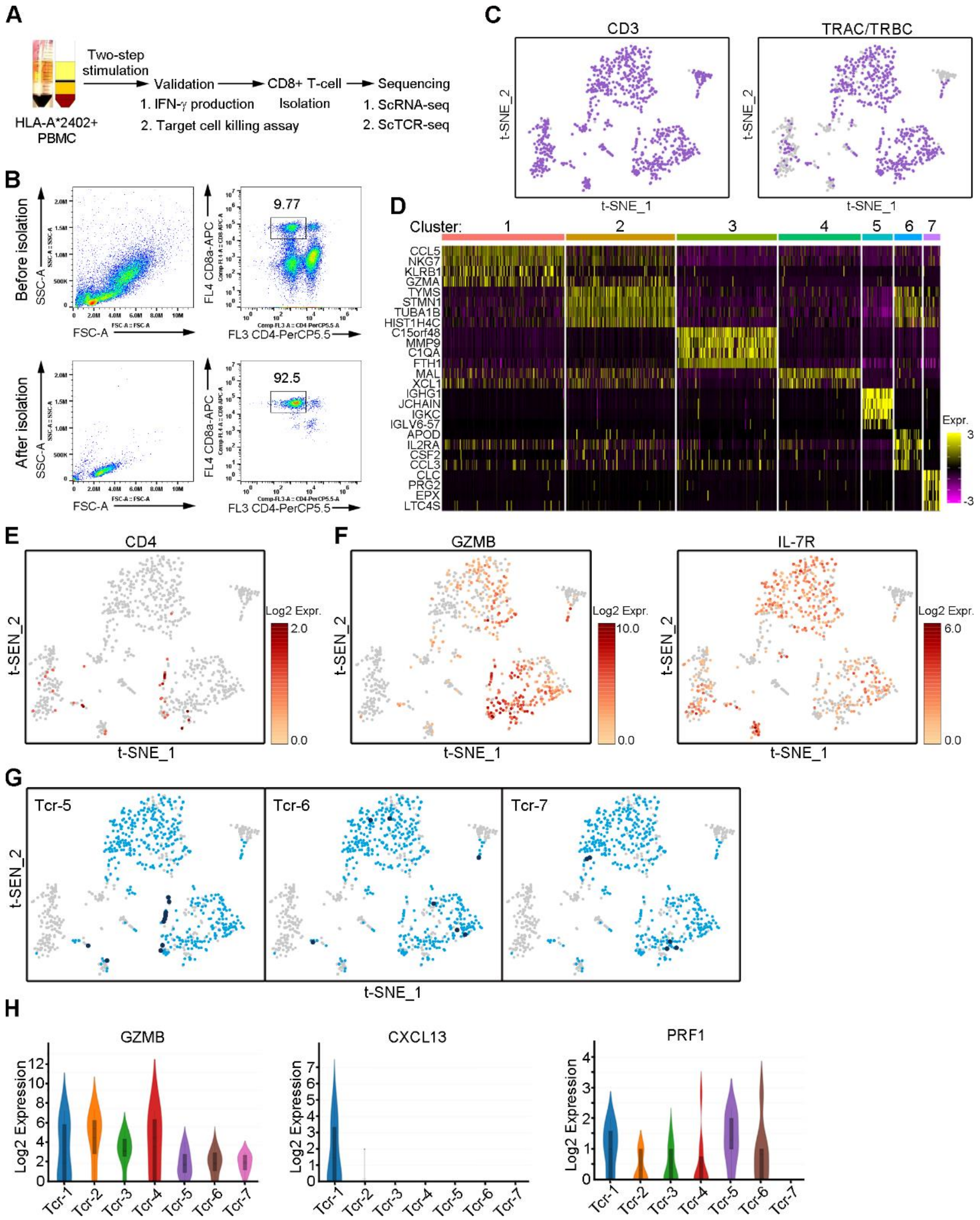

Appendix Figure S3. ScRNA-seq and scTCR-seq revealed Pep-4-specific TCR repertoire **(A)** Schematic image for performing scRNA-seq and scTCR-seq. **(B)** Representative flow cytometric image showed a purity of CD8+ T cells after magnetic cell sorting. **(C)** Independent tSNE plots of isolated NRT cells. CD3+ cells and TRAC/TRBC+ cells were highlighted. **(D)** Heatmap showing differentially expressed genes between the 7 clusters of expanded CD8+ T cells. **(E-F)** Independent tSNE plots of isolated NRT cells. CD4+ T cells (E), GZMB+ (F, left), and IL-7R+ (F, right) T cells were highlighted. **(G)** Tcr-5-positive, Tcr-6-positive, and Tcr-7-positive T cells were highlighted. **(H)** Violin plots of predictive NRT marker genes in each subclonal T cell.

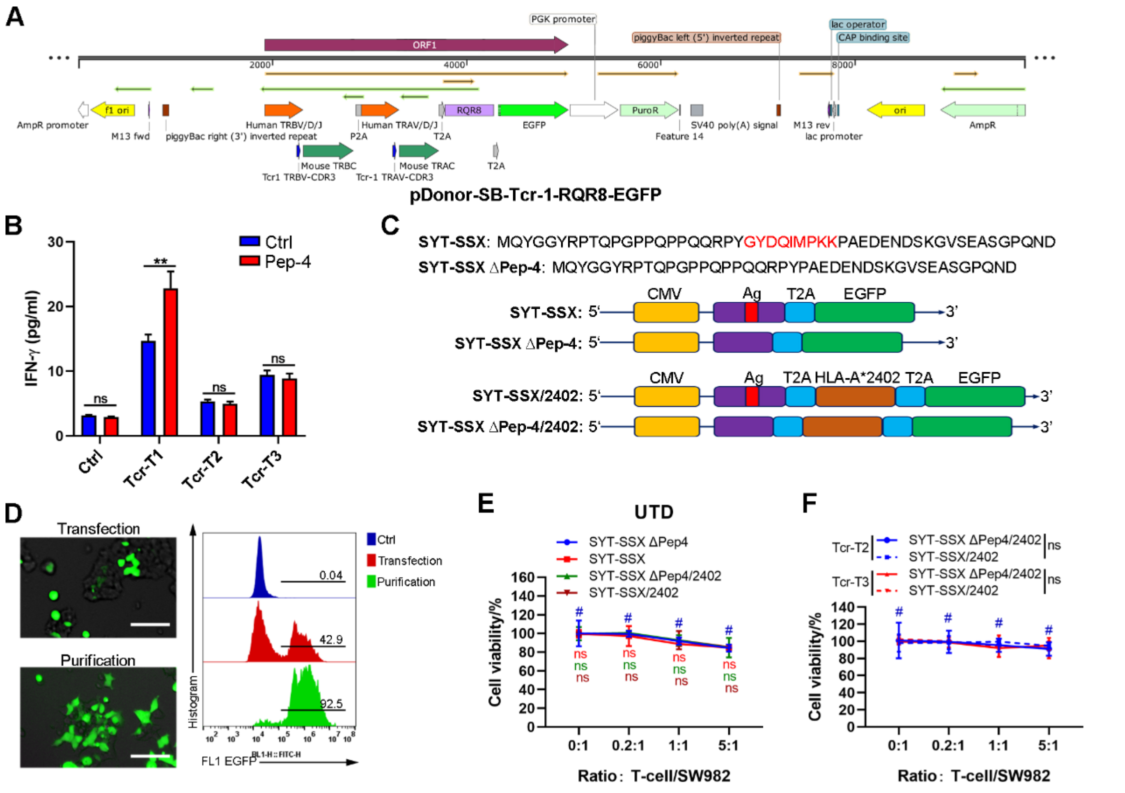

Appendix Figure S4. Tcr-T1 cells exhibited antigen-specific cytotoxicity against HLA-A*2402-positive synovial sarcoma cells

(A) Schematic image of the construction of TCR-expressing vector. **(B)** TCR-T cells that expressed Tcr-T1, Tcr-T2, and Tcr-T3 were generated as mentioned in methods. Subsequently, they were used to incubate with target cells (MKN45) at a ratio of E: T=1:1, with or without Pep-4 pulsing. IFN-γ production was determined by using CBA. **(C-D)** Construction of synovial sarcoma target cells based on cell line SW982. DNA fragments of SYT-SSX fusion mutation containing/deleting Pep-4 sequencing, or DNA coding HLA-A*2402 were cloned singly or combined into expressing vectors (C). These vectors were then transfected into synovial sarcoma cell line SW982, in order to form target cells, including SYT-SSX ΔPep-4 (expressing SYT-SSX fusion protein deleting Pep-4), SYT-SSX Pep-4 (expressing SYT-SSX fusion protein containing Pep-4), SYT-SSX ΔPep-4/2402 (expressing SYT-SSX fusion protein deleting Pep-4, and HLA-A*2402), and SYT-SSX Pep-4 (expressing SYT-SSX fusion protein containing Pep-4, and HLA-A*2402). They were purified by flow cytometry sorting (D). **(E)** Target cells derived from (D) were incubated with UTD T cells at ratio 0:1, 0.2:1, 1:1, and 5:1 (T-cell/SW982). Cell viability was determined by using CCK-8. **(F)** Target cells derived from (D) were incubated with TCR-T cells expressing Tcr-T2, and Tcr-T3, respectively, at ratio 0:1, 0.2:1, 1:1, and 5:1 (T-cell/SW982). Cell viability was determined by using CCK-8. Data are presented as mean ± SEM. Statistical significance was calculated using the one-way ANOVA (B), two-way ANOVA (E-F).. ns, not significant, $p < 0.05$, *; $p < 0.01$ **; $p < 0.001$, ***.

A

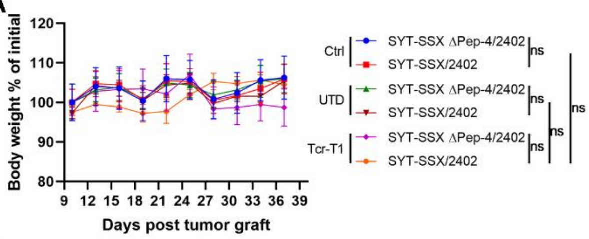

B

Serum biochemical parameters

Parameters	Ctrl			UTD			Tcr-T1		
	SYT-SSX ΔPep-4/2402	SYT-SSX/2402	P value	SYT-SSX ΔPep-4/2402	SYT-SSX/2402	P value	SYT-SSX ΔPep-4/2402	SYT-SSX/2402	P value
ALT (U/L)	0.57±0.24	0.74±0.1	0.528	0.61±0.21	0.46±0.19	0.837518	0.70±0.06	0.53±0.22	0.501899
AST (U/L)	10.97±0.68	10.13±0.81	0.476	8.6±0.5	7.93±0.91	0.559178	8.8±1.56	8.73±0.30	0.968563
Total bilirubin (mmol/L)	0.27±0.03	0.25±0.03	0.778	0.23±0.03	0.24±0.03	0.833566	0.23±0.03	0.23±0.03	1
Direct Bilirubin (mmol/L)	0.13±0.09	0.07±0.03	0.565	0.06±0.03	0.07±0.04	0.900148	0.06±0.03	0.06±0.03	1
Total protein (g/L)	5.53±0.12	5.58±0.15	0.818	5.73±0.07	5.67±0.19	0.752315	5.40±0.10	5.77±0.09	0.051374
Albumin (g/L)	4.67±0.14	4.71±0.17	0.847	4.60±0.10	4.66±0.18	0.767644	4.43±0.08	4.77±0.09	0.055654
Globulin (g/L)	0.86±0.03	0.87±0.03	1.000	1.03±0.06	0.97±0.09	0.579015	0.96±0.03	1.00±0.1	0.373901
Albumin/Globulin ratio	5.41±0.37	5.49±0.39	0.885	4.59±0.32	4.67±0.19	0.83813	4.59±0.17	4.77±0.09	0.425517
Urea (mmol/L)	0.67±0.07	0.62±0.02	0.539	0.67±0.09	0.57±0.07	0.416866	0.53±0.03	0.56±0.07	0.677869
Creatine (mmol/L)	1.67±0.33	1.73±0.13	0.862	1.33±0.3	1.53±0.30	0.674518	1.66±0.33	1.20±0.20	0.296181

C

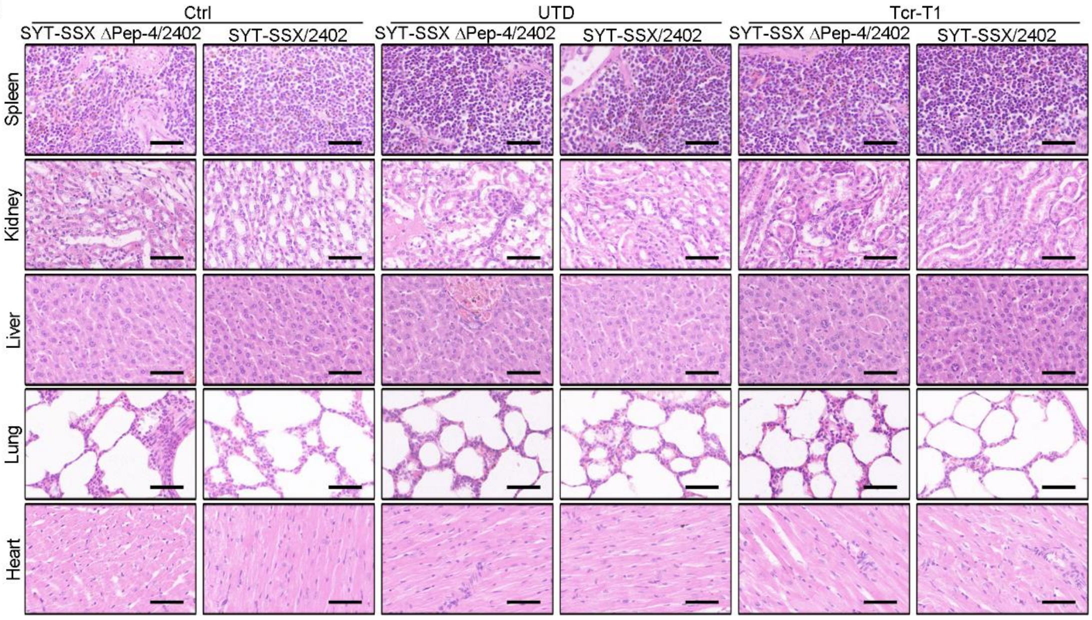

Appendix Figure S5. Examination of toxicity of transfer of Tcr-T1 cells

(A-C) Prepared synovial sarcoma target cells were subcutaneously injected to form a tumor-bearing mouse model. Tcr-T1 cells or UTD T cells were transferred, and body weight was recorded (A). At the endpoint, mice were sacrificed and serum biochemical value was analyzed. Serum aspartate aminotransferase (AST), alanine aminotransferase (ALT), total bilirubin, directed bilirubin, total protein, albumin, globulin, albumin/globulin ratio, urea, creatine were assayed as an indicator of liver and renal functions (B). Major organs were collected from various groups, and subjected to H&E staining (C). Scale bar = 50 μm. Data are presented as mean ± SEM. Statistical significance was calculated using the student's t-test (B), and two-way ANOVA (A). ns, not significant, $p < 0.05$, *; $p < 0.01$ **; $p < 0.001$, ***.

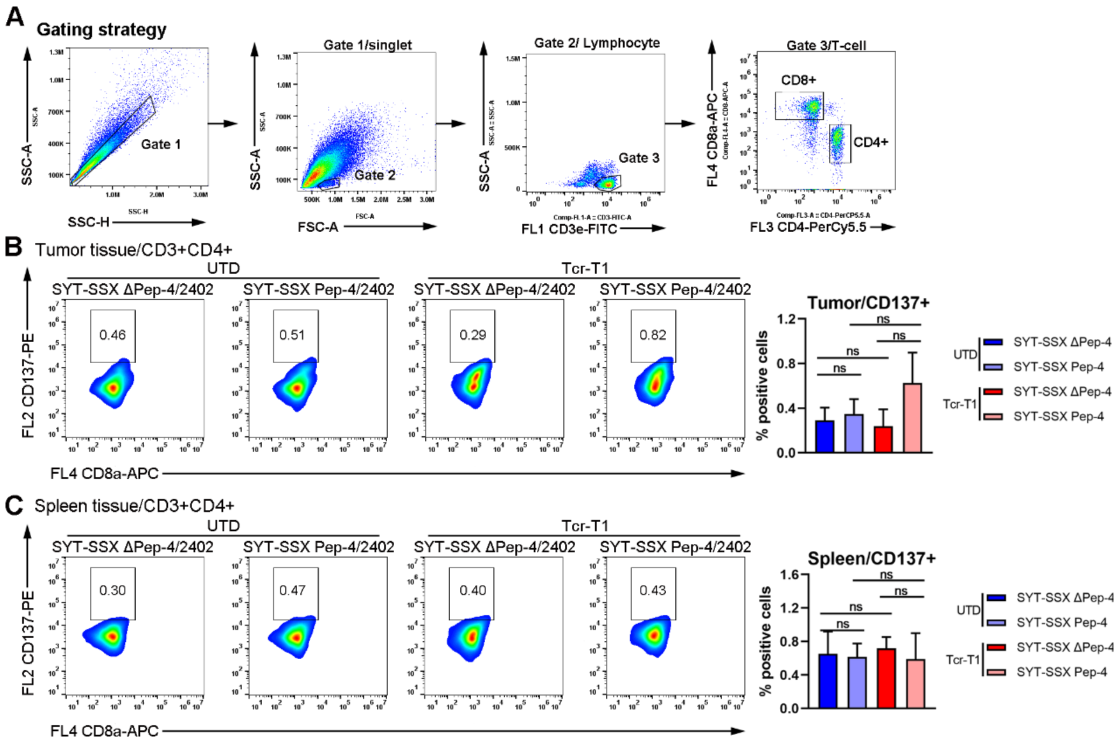

Appendix Figure S6. Flow cytometric analysis of T-cell activation in tumor and spleen tissues.

(A-C) Tumor tissues and spleen tissues were isolated from mice from Figure S5, and subjected to flow cytometric analysis. The gating strategy was shown (A). Representative flow cytometric image of CD137 expression on $CD3^{+}CD4^{+}$ T cells in tumor tissues (B, left), and spleen (C, left) (n=3). The percentage of CD137-positive T cells in tumor tissues (B, right) and spleen (C, right) was summarized. Data are presented as mean ± SEM. Statistical significance was calculated using the one-way ANOVA (B-C). ns, not significant.

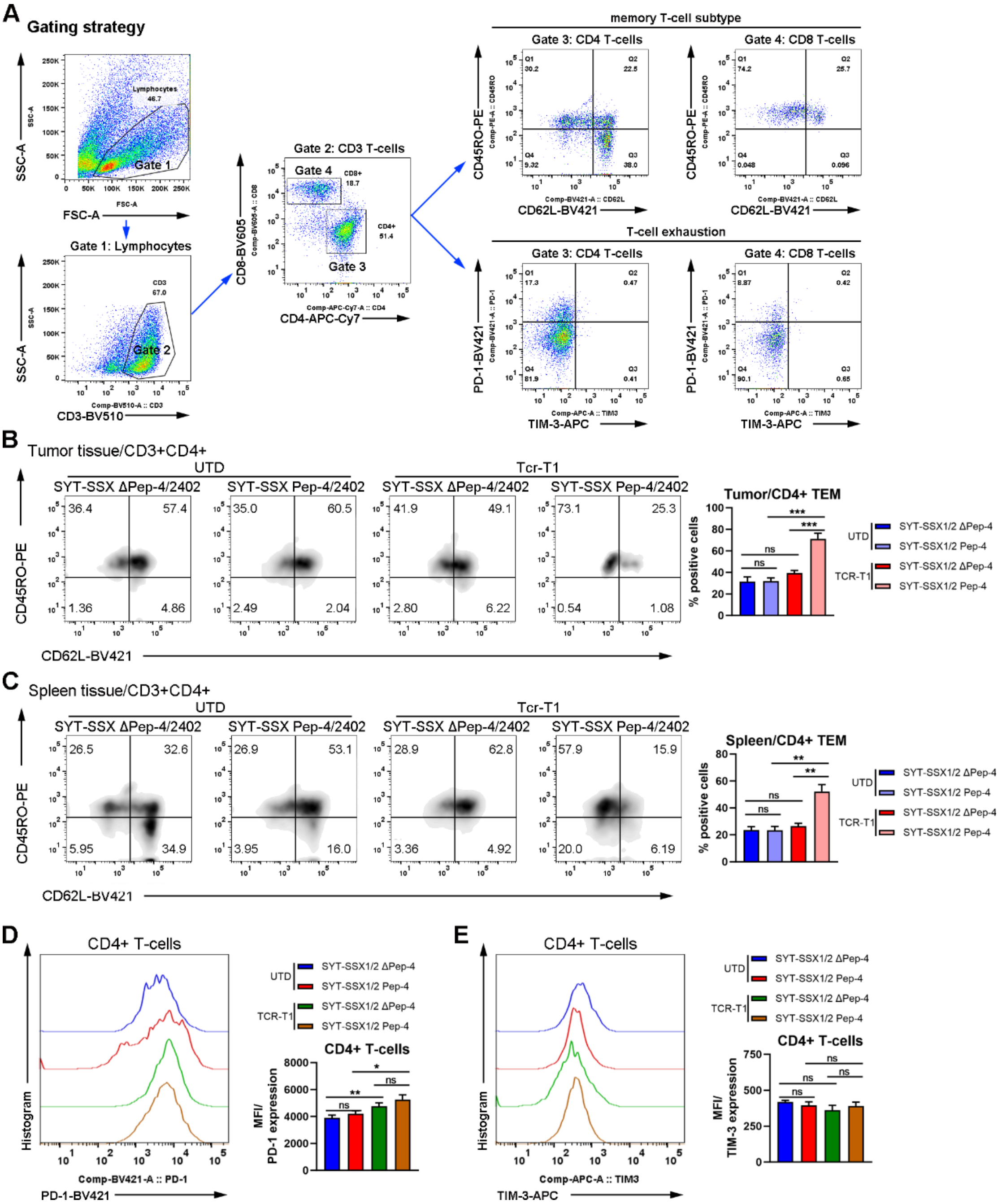

Appendix Figure S7. Flow cytometric analysis of memory T-cell subsets and exhaustion. (A) Tumor tissues and spleen tissues were isolated from mice from Figure S5, and subjected to flow cytometric analysis for examination of memory T-cell subsets and exhaustion. A gating strategy was shown. **(B-C)** Flow cytometric analysis of effector memory CD4+ T-cell (TEM) differentiation, characterized as CD45RO^{+}CD62L^{-} (n=3). Representative flow cytometric image of detecting TEM T cells in tumor tissues (B, left), and spleen (C, left) (n=3). The percentage of TEM in tumor tissues (B, right) and spleen (C, right) was summarized. **(D-E)** Flow cytometric analysis of T-cell exhaustion, characterized as PD-1, and TIM-3 (n=3). Representative flow cytometric image of PD-1 (D, left), and Tim-3 expression (E, left) in CD4+ T-cells in tumor tissues (n=3). The MFI of PD-1 (D, right), and Tim-3 (E, right) expression were summarized.

Statistical significance was calculated using the one-way ANOVA (B-C). ns, not significant, $p < 0.05$, *; $p < 0.01$ **; $p < 0.001$, ***.

Appendix Table S1. Top 7 Clonotype CDR3 Sequences

Name	Chain	V	CDR3 sequence	J	C
Tcr-1	TRA	TRAV12-1	CVALDGQKLLF	TRAJ16	TRAC
	TRB	TRBV3-1	CASSGAGSYNEQFF	TRBJ2-1	TRBC2
Tcr-2	TRA	TRAV3	CAVRDISSGTYKYIF	TRAJ40	TRAC
	TRB	TRBV6-5	CASSQLTGGGYTEAFF	TRBJ1-1	TRBC1
Tcr-3	TRA	TRAV8-4	CAVSDSDFGNEKLTF	TRAJ48	TRAC
	TRB	TRBV6-5	CASSYRGLAAYNEQFF	TRBJ2-1	TRBC2
Tcr-4	TRA	TRAV29	CAVRVLSGTYKYIF	TRAJ23	TRAC
	TRB	TRBV5-1	CASSPNLQIIQETQYF	TRBJ1-3	TRBC1
Tcr-5	TRA	TRAV3	CAAFPYNQGGKLIF	TRAJ40	TRAC
	TRB	TRBV6-2	CASSVSGTASSGNTIYF	TRBJ2-5	TRBC2
Tcr-6	TRA	TRAV12-1	CVAHSGTYKYIF	TRAJ40	TRAC
	TRB	TRBV24-1	CATSGTNTGLAGAQYF	TRBJ2-3	TRBC2
Tcr-7	TRA	TRAV1-2	CAVRVASGGSYIPTF	TRAJ6	TRAC
	TRB	TRBV4-1	CASSQEGLNQPQHF	TRBJ1-5	TRBC1

Appendix Table S2. AA sequences of synthetic TCR

Description	Sequence
human TRBV/BD/BJ with CDR3 region underlined	MAIGLLCCVAFSLLWASPVNAGVTQTPKFQVLKTGQSMTLQCAQDMNHNSMYWYRQDPGMGLRLIYYSASEGTTDKGEVPNGYNVSRLNKREFSLRLESAAPSQTSVYF<u>CASSGAGSYNEQFF</u>GPGTRLTVT
murine TCRB constant region	EDLRNVTPPKVSLFEPSKAEIANKQKATLVCLARGFFPDHVELSWWVNGKEVHSGVCTDPQAYKESNYSYCLSSRLRVSATFWHNPRNHFRCQVQFHGLSEEDKWPEGSPKPVTQNISAEAWGRADCGITSASYQQGVLSATILYEILLGKATLYAVLVSTLVVMAMVKRKNS
human TRAV/AJ with CDR3 region underlined	MISLRVLLVILWLQLSWVWSQRKEVEQDPGPFNVPEGATVAFNCTYSNSASQSFFWYRQDCRKEPKLLMSVYSSGNEDGRFTAQLNRASQYISLLIRDSKLSDSATY<u>LCVALDGQKLLF</u>GTGTKLQVIP
murine TCRA constant region	DIQNPEPAVYQLKDPRSQDSTLCLFTDFDSQINVPKTMESGTFITDKCVLDMKAMDSKSNGAIAWSNQTSFTCQDIFKETNATYPSSDVPCDATLTEKSFETDMNLNFQNLLVIVLRILLLKVAGFNLLMTLRLWSS

RQR8 epitope	MGTSLLCWMALCLLGADHADACPYSNPSLCSGGGGSELPT QGTFSNVSTNVSPAKPTTTACPYSNPSLCSGGGGSPAPRPP TPAPTIASQPLSLRPEACRPAAGGAVHTRGLDFACDIYIWAPL AGTCGVLLLSLVITLYCNHRNRRRVCKCPRPVV

Appendix Table S3. Properties of Pep-4 NP

Group Name	Pep-4 (mg)	mPEG-PVGLIG-PCL (mg)	Treatment	Loading Capacity (%)	Encapsulation Efficiency (%)
1	0.5	5	Freshly prepared	6.45±1.23	60.3±2.5
2	2	5	Freshly prepared	9.85±0.86	42.6±1.1
3	10	5	Freshly prepared	9.35±1.06	5.1±0.2
4	2	5	4°C, 24 h	9.92±0.48	
5	2	5	37°C, 24 h	9.66±0.72	
6	2	5	4°C, 14 d	8.26±0.81	
