# Supplementary figures and images for: A therapeutic regimen using neoantigen-specific TCR-T cells for HLA-A*2402-positive solid tumors (part 1 of 2)
Source: EMBO Mol Med. 2025 Jan 2;17(2):365–83. doi: 10.1038/s44321-024-00184-1 (PMC11821884; doi:10.1038/s44321-024-00184-1)

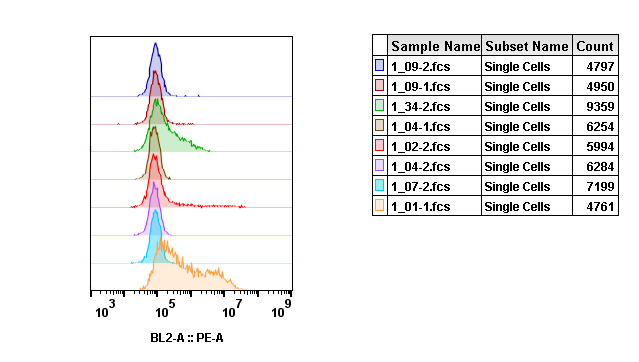

Supplement: Supplementary file 3 — Source data Fig. 1 [file 44321_2024_184_MOESM3_ESM.zip › Fig 1/Fig 1G/09-Nov-2022-Layout-1.png]

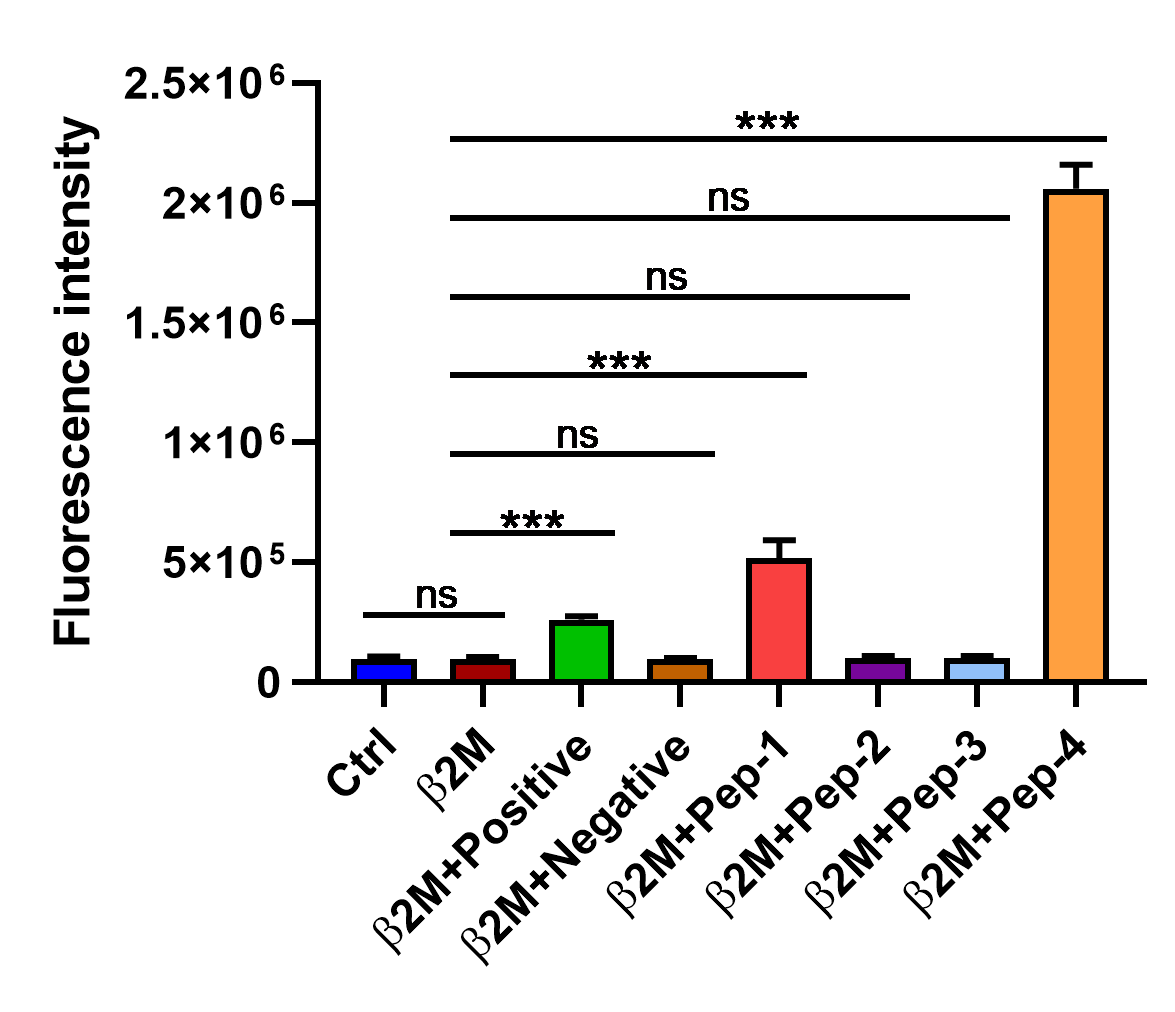

Supplement: Supplementary file 3 — Source data Fig. 1 [file 44321_2024_184_MOESM3_ESM.zip › Fig 1/Fig 1G/1.tif]

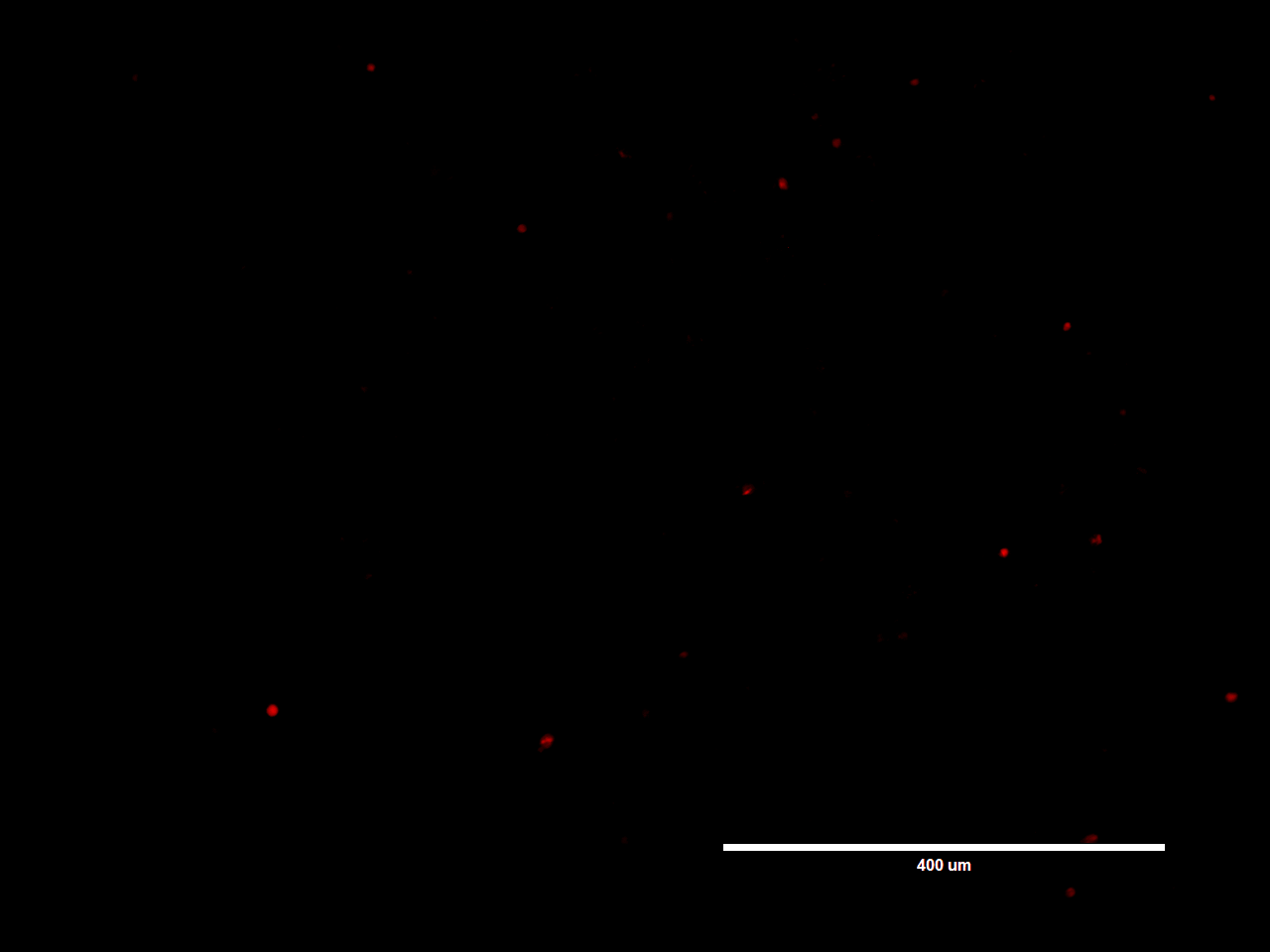

Supplement: Supplementary file 3 — Source data Fig. 1 [file 44321_2024_184_MOESM3_ESM.zip › Fig 1/Fig 1H/c2-r.tif]

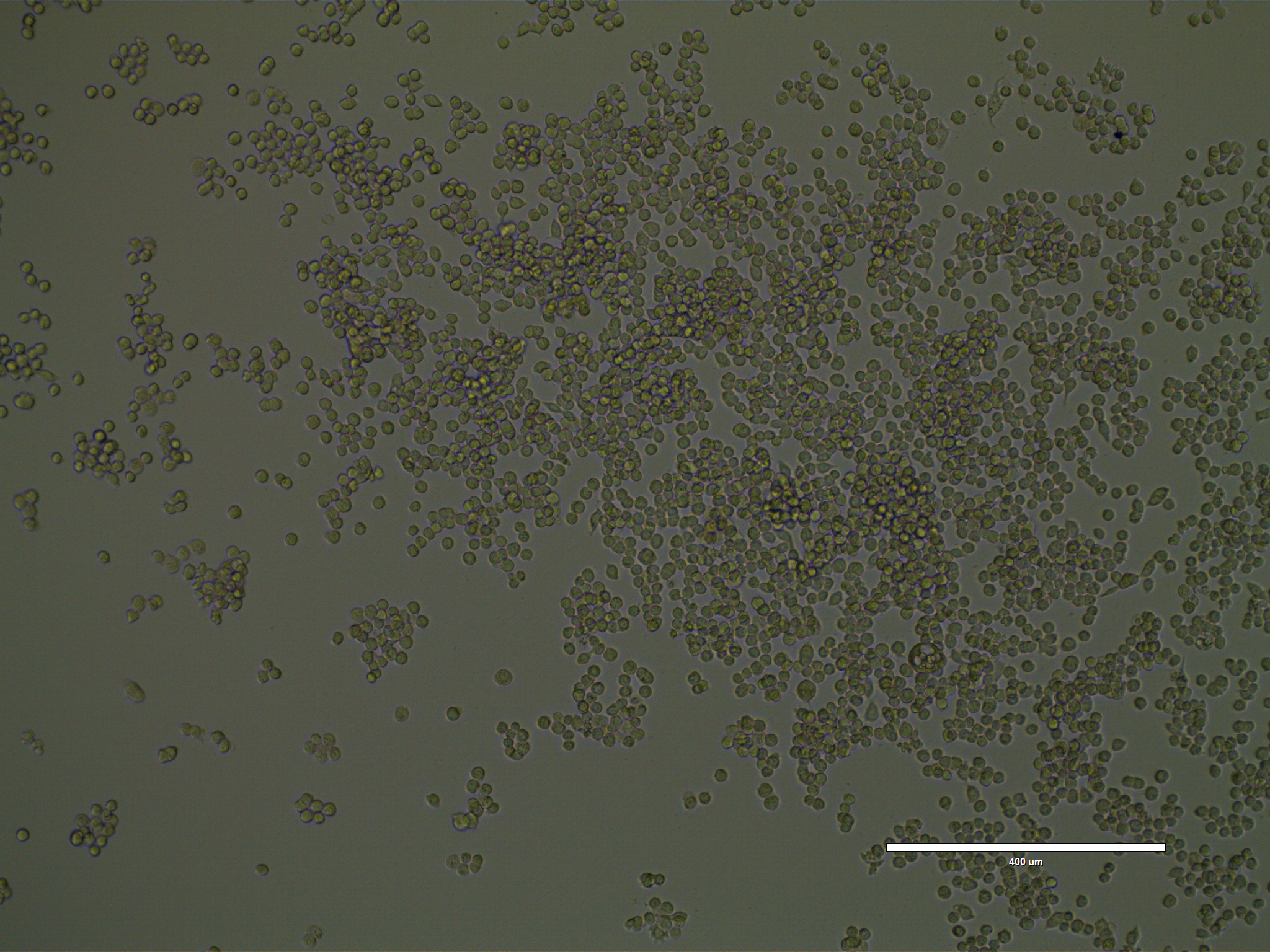

Supplement: Supplementary file 3 — Source data Fig. 1 [file 44321_2024_184_MOESM3_ESM.zip › Fig 1/Fig 1H/c2-w.tif]

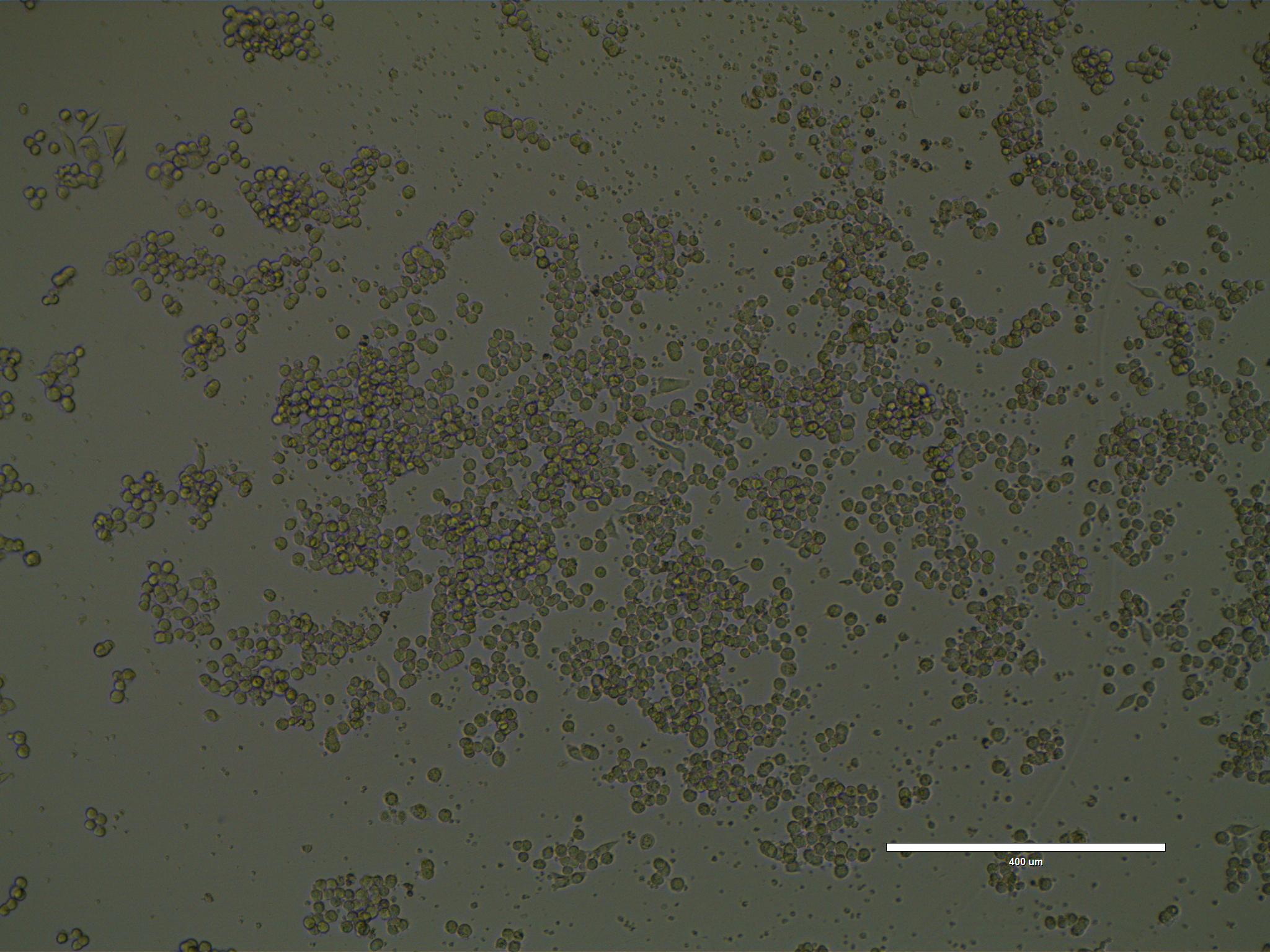

Supplement: Supplementary file 3 — Source data Fig. 1 [file 44321_2024_184_MOESM3_ESM.zip › Fig 1/Fig 1H/c5-w.tif]

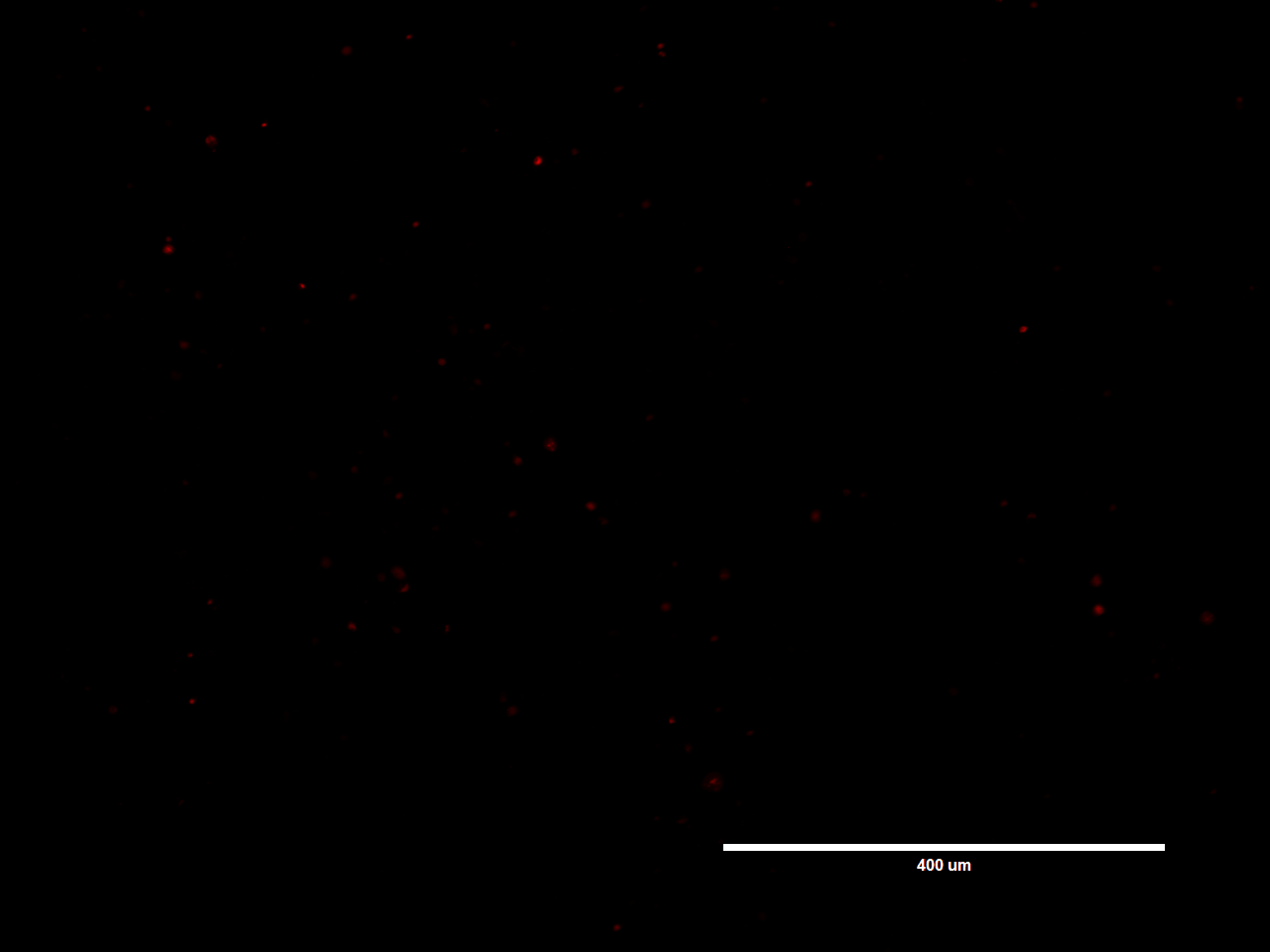

Supplement: Supplementary file 3 — Source data Fig. 1 [file 44321_2024_184_MOESM3_ESM.zip › Fig 1/Fig 1H/c5-r.tif]

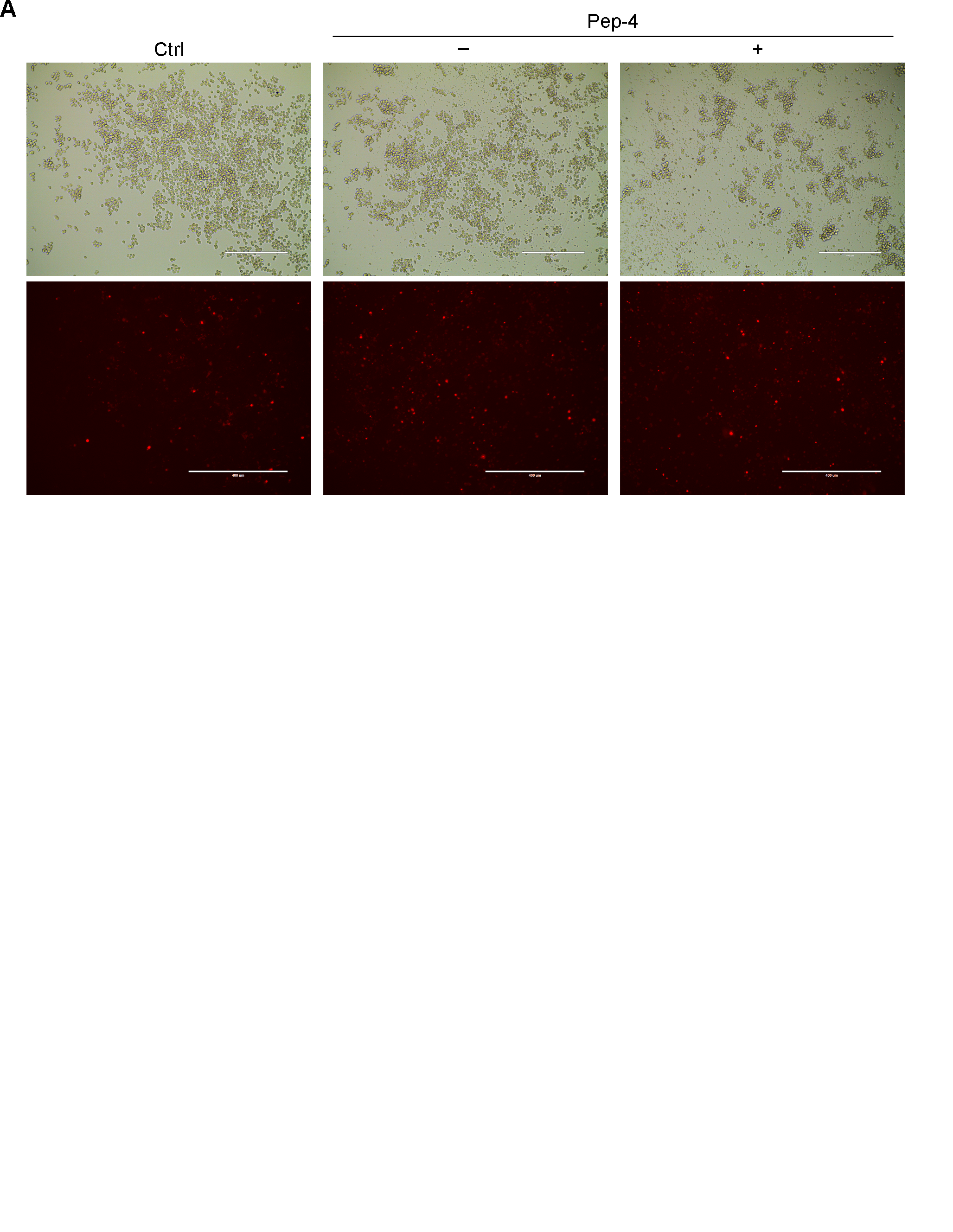

Supplement: Supplementary file 3 — Source data Fig. 1 [file 44321_2024_184_MOESM3_ESM.zip › Fig 1/Fig 1H/res1.tif]

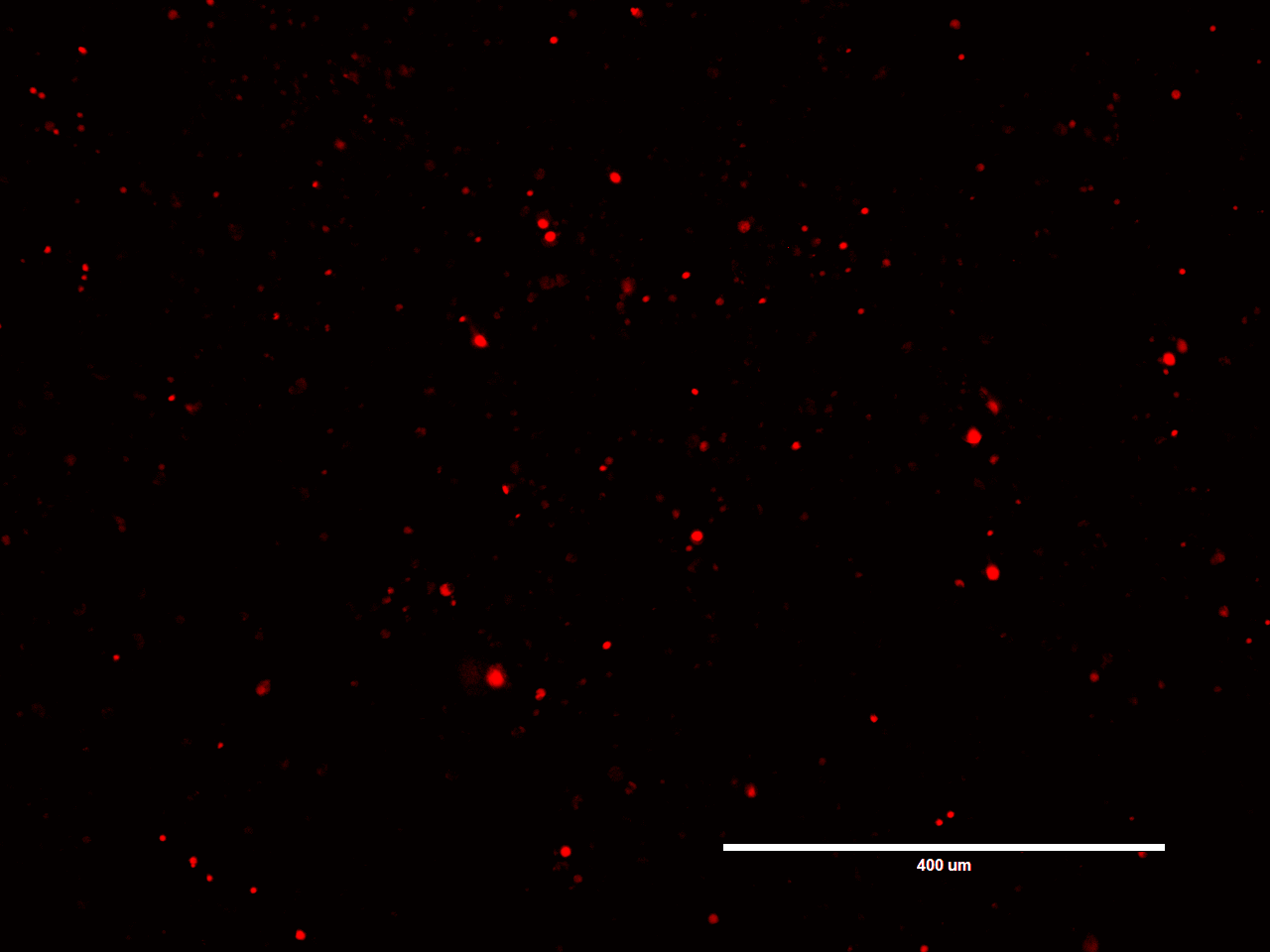

Supplement: Supplementary file 3 — Source data Fig. 1 [file 44321_2024_184_MOESM3_ESM.zip › Fig 1/Fig 1H/c10-r.tif]

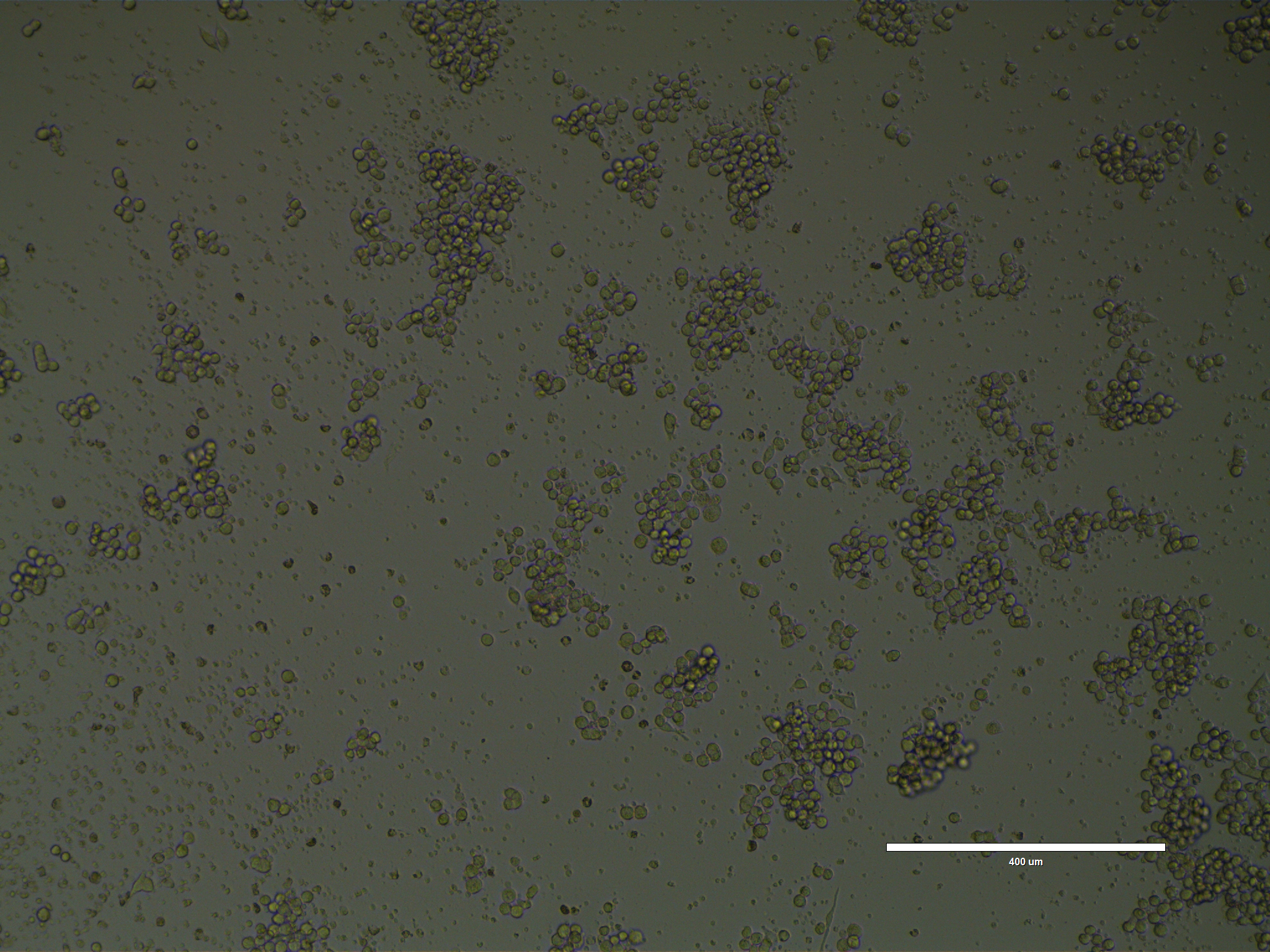

Supplement: Supplementary file 3 — Source data Fig. 1 [file 44321_2024_184_MOESM3_ESM.zip › Fig 1/Fig 1H/c10-w.tif]

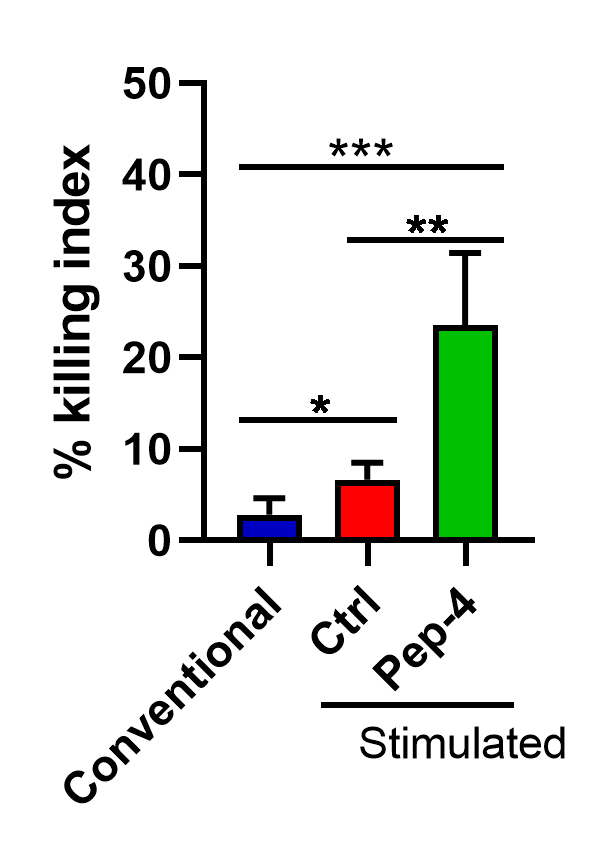

Supplement: Supplementary file 3 — Source data Fig. 1 [file 44321_2024_184_MOESM3_ESM.zip › Fig 1/Fig 1H/Data 1.tif]

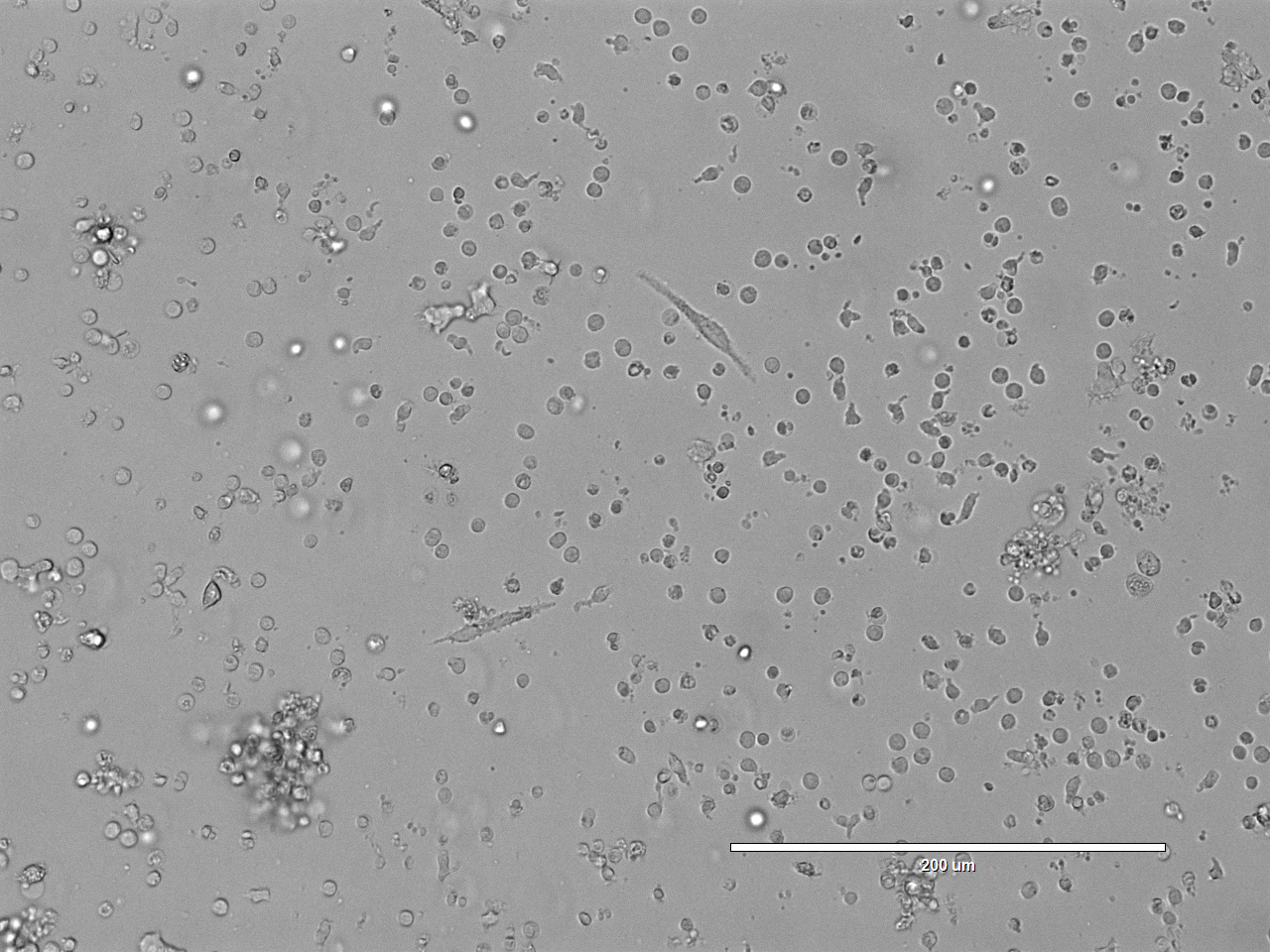

Supplement: Supplementary file 3 — Source data Fig. 1 [file 44321_2024_184_MOESM3_ESM.zip › Fig 1/Fig 1C/2.tif]

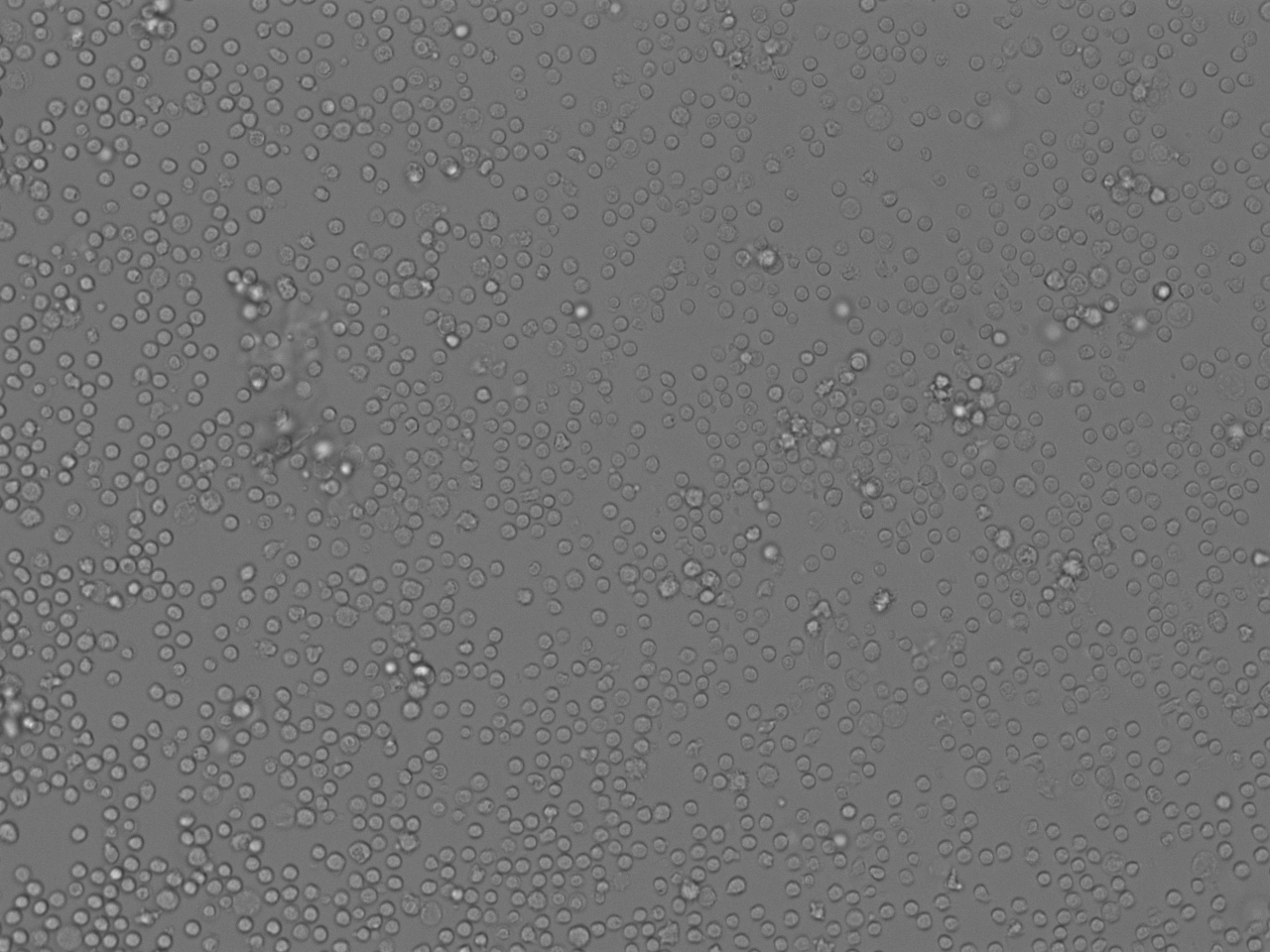

Supplement: Supplementary file 3 — Source data Fig. 1 [file 44321_2024_184_MOESM3_ESM.zip › Fig 1/Fig 1C/1.tif]

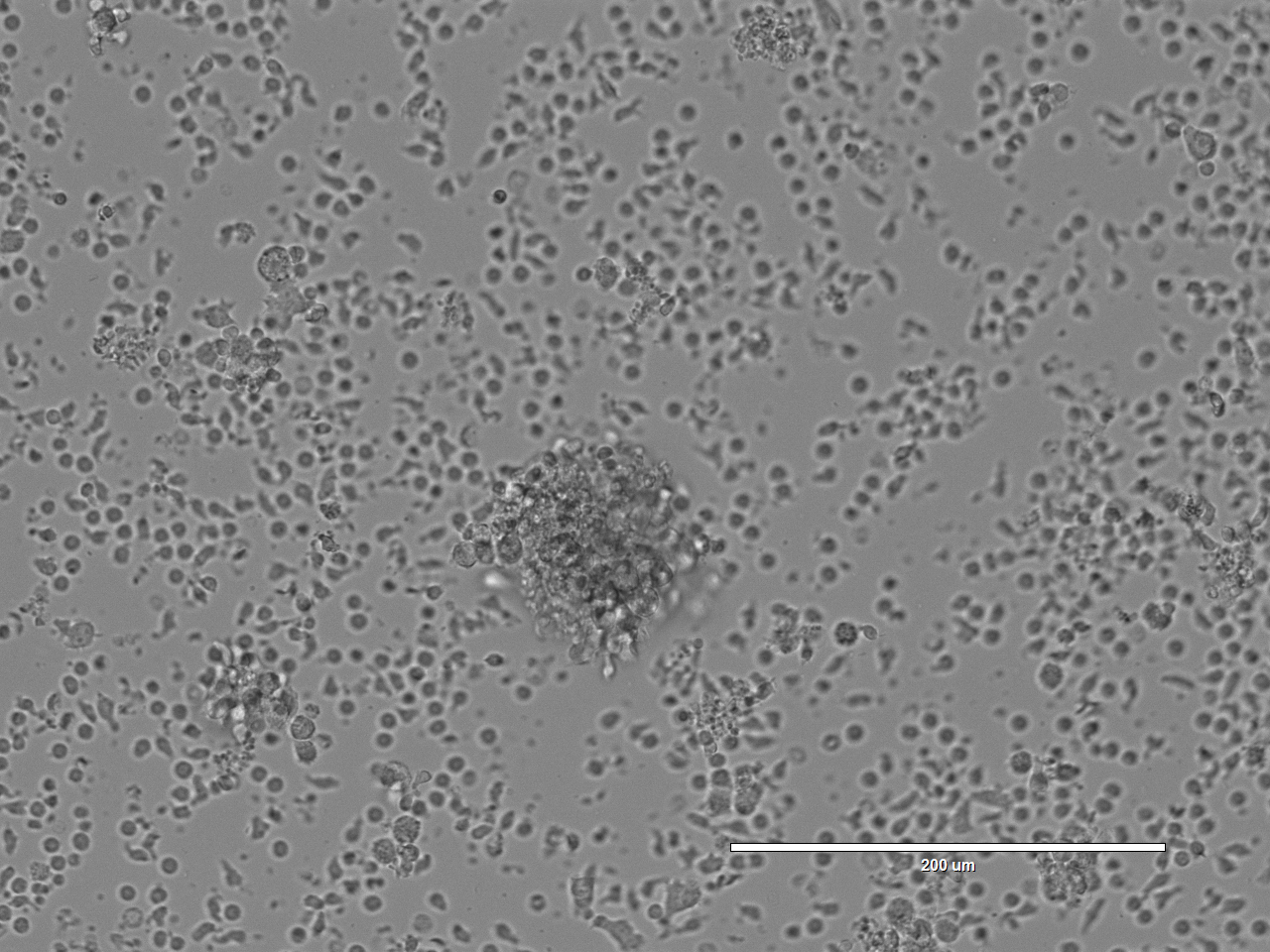

Supplement: Supplementary file 3 — Source data Fig. 1 [file 44321_2024_184_MOESM3_ESM.zip › Fig 1/Fig 1C/4.tif]

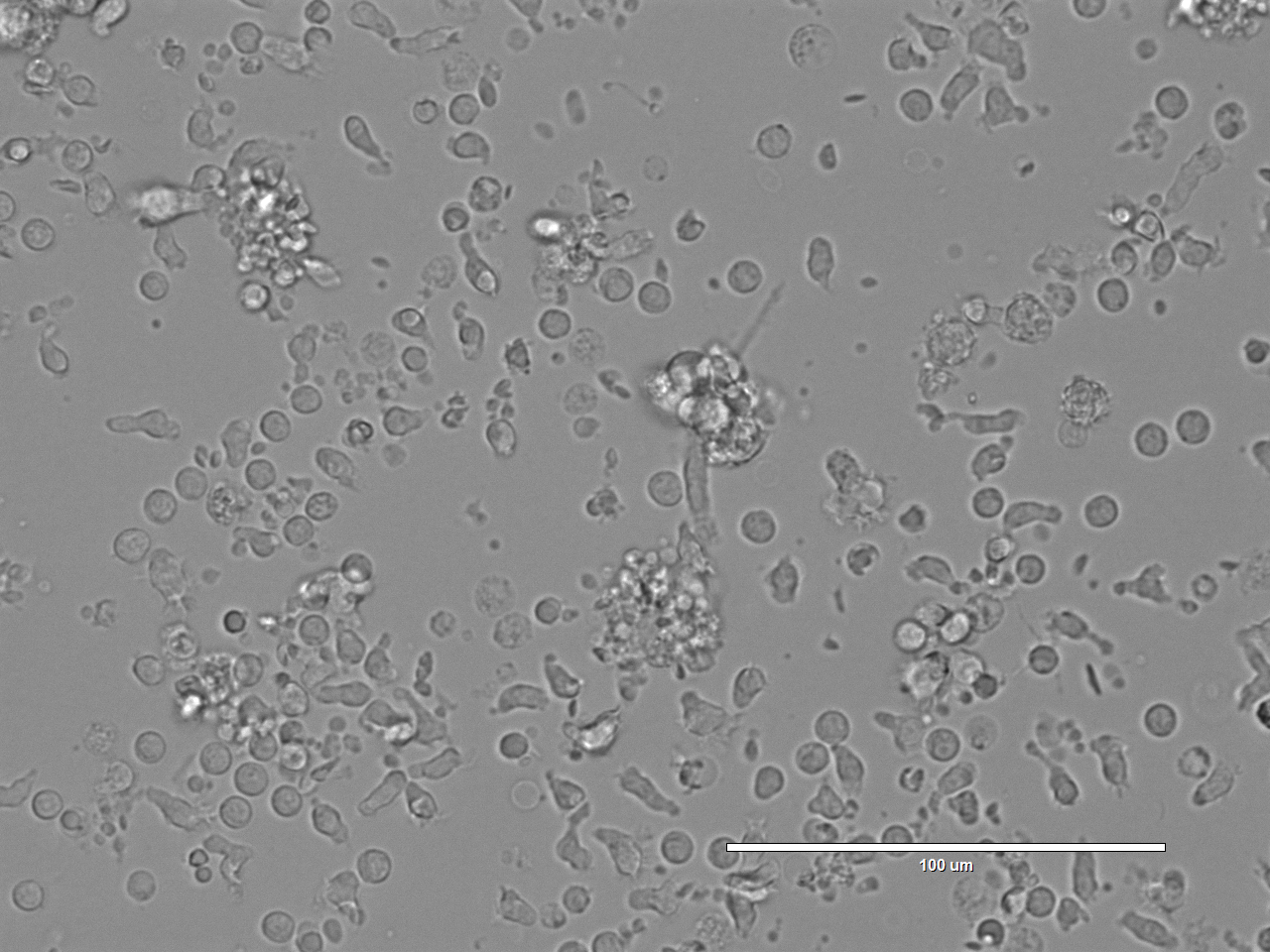

Supplement: Supplementary file 3 — Source data Fig. 1 [file 44321_2024_184_MOESM3_ESM.zip › Fig 1/Fig 1C/3-3.tif]

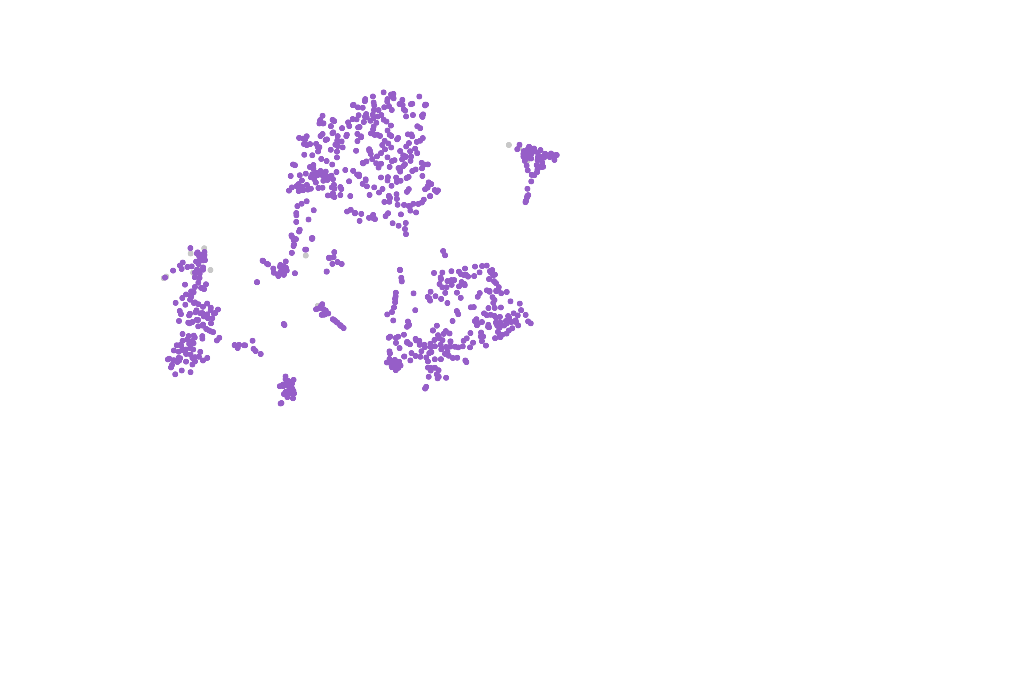

Supplement: Supplementary file 4 — Source data Fig. 2 [file 44321_2024_184_MOESM4_ESM.zip › Fig 2/cd3.png]

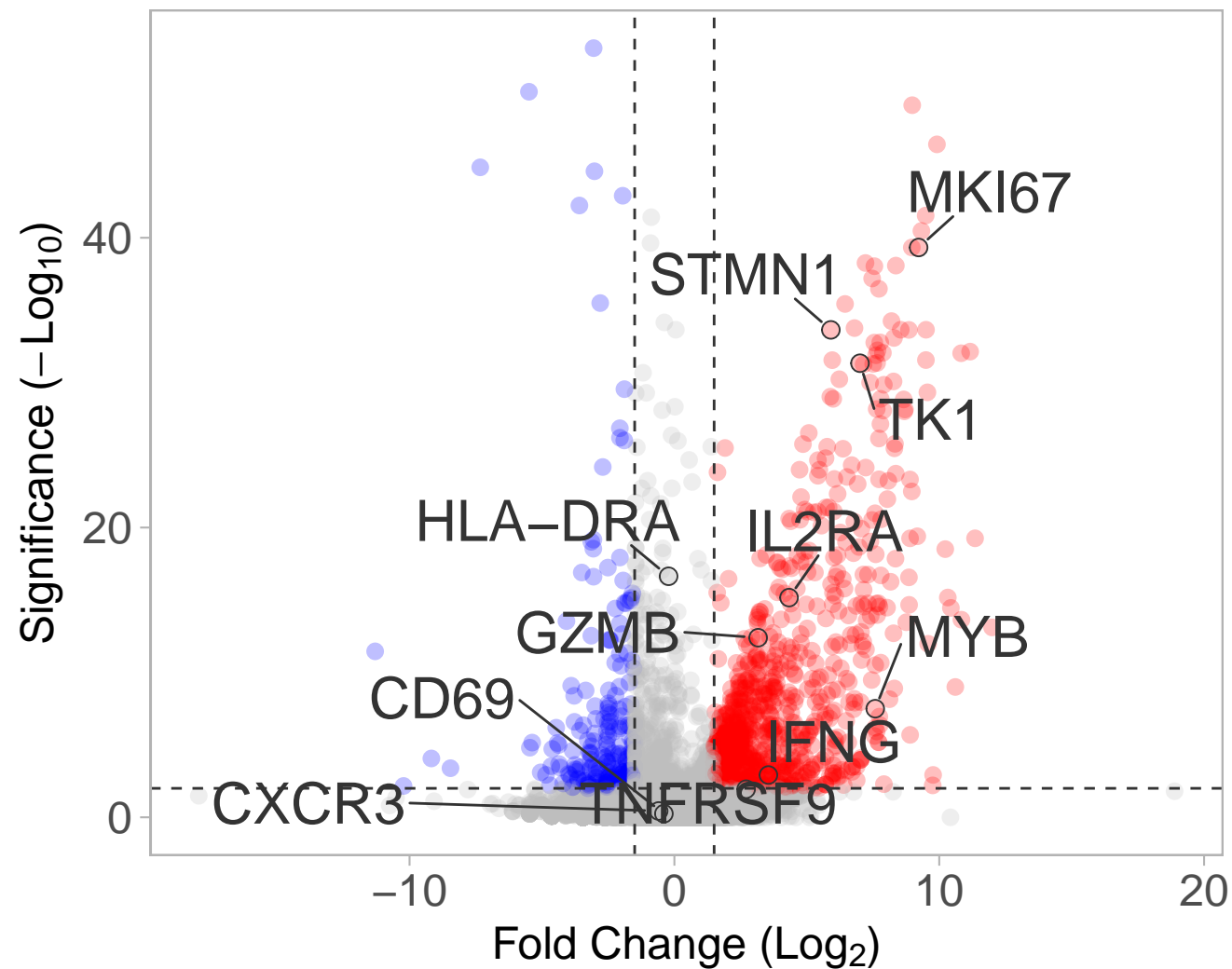

Supplement: Supplementary file 4 — Source data Fig. 2 [file 44321_2024_184_MOESM4_ESM.zip › Fig 2/VolcaNoseR_2024-07-31 08_50_07.591668.pdf]

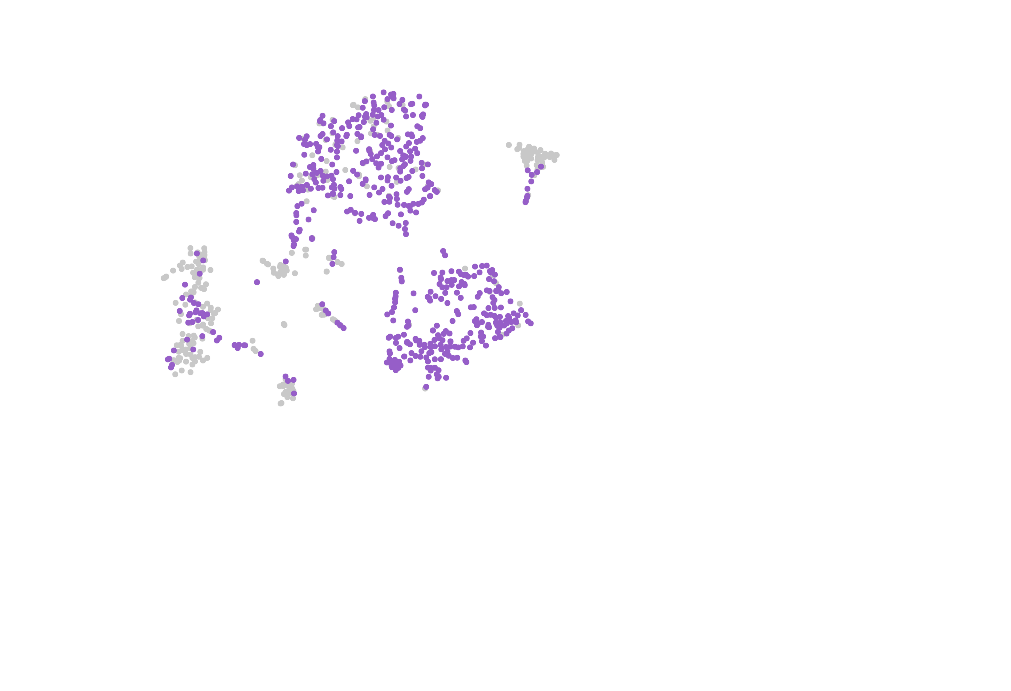

Supplement: Supplementary file 4 — Source data Fig. 2 [file 44321_2024_184_MOESM4_ESM.zip › Fig 2/TRA and TRC.png]

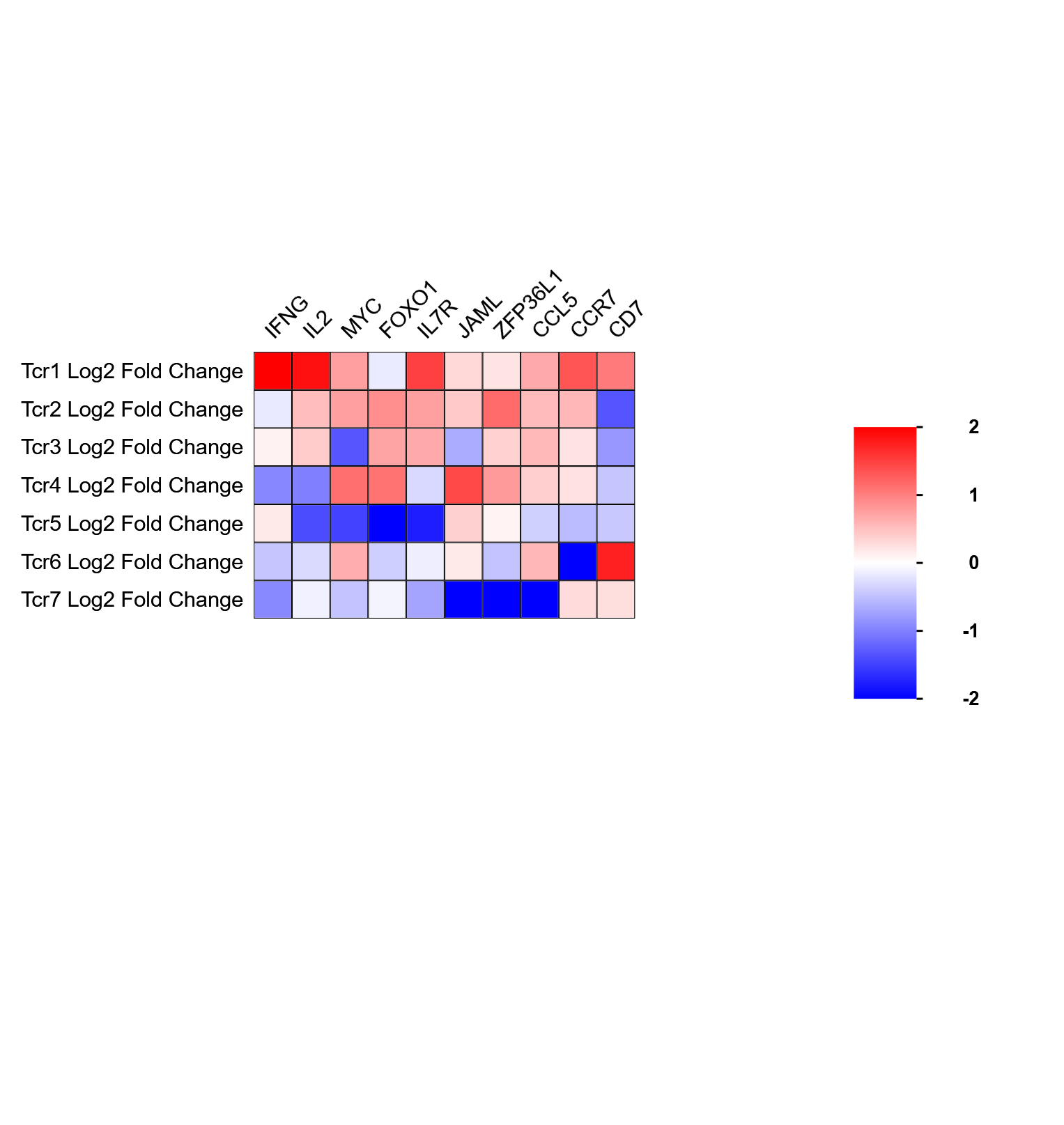

Supplement: Supplementary file 4 — Source data Fig. 2 [file 44321_2024_184_MOESM4_ESM.zip › Fig 2/image.png]

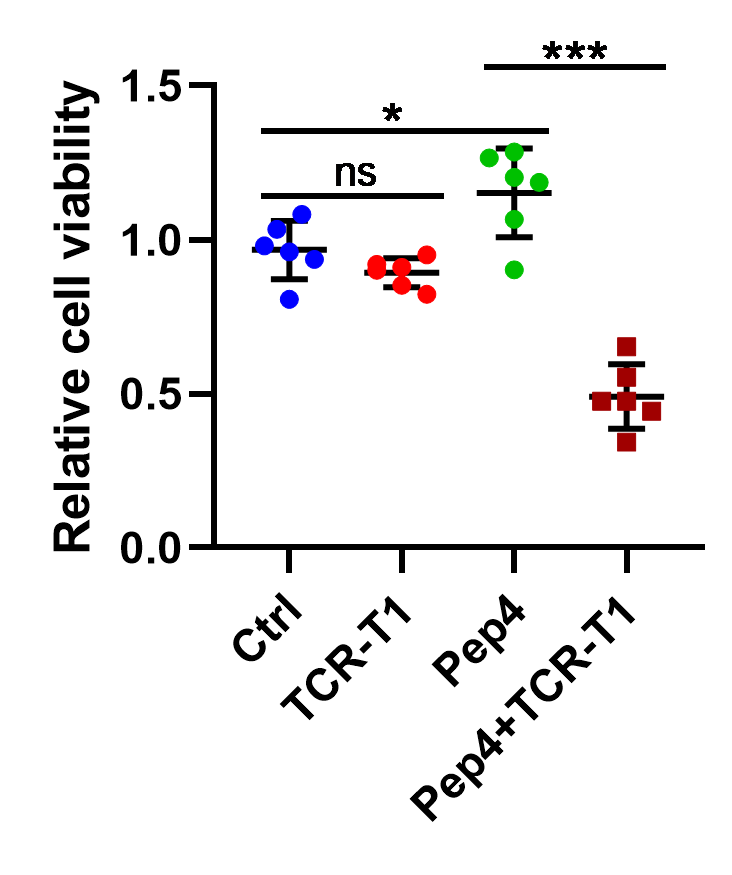

Supplement: Supplementary file 5 — Source data Fig. 3 [file 44321_2024_184_MOESM5_ESM.zip › Fig 3/Fig 3C/1.tif]

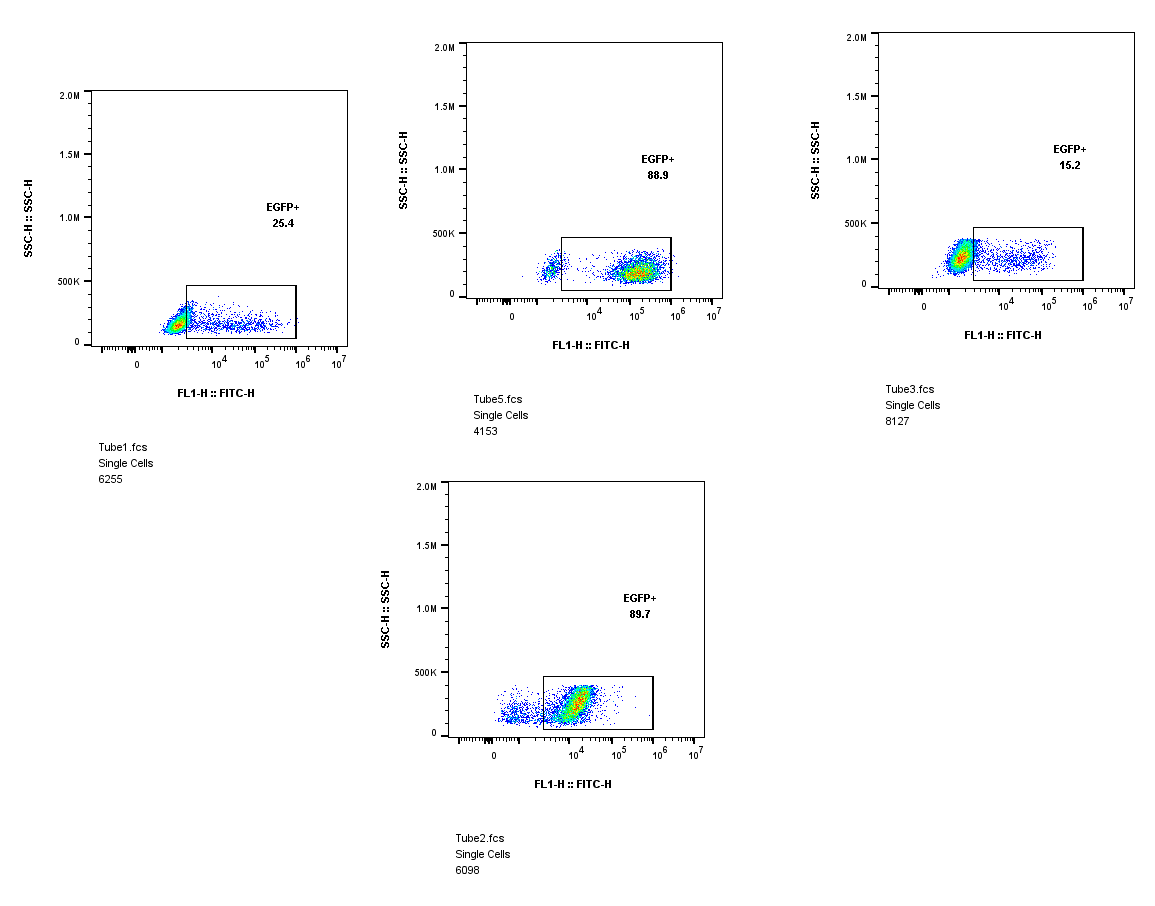

Supplement: Supplementary file 5 — Source data Fig. 3 [file 44321_2024_184_MOESM5_ESM.zip › Fig 3/Fig 3B/17-Feb-2022-Layout.png]

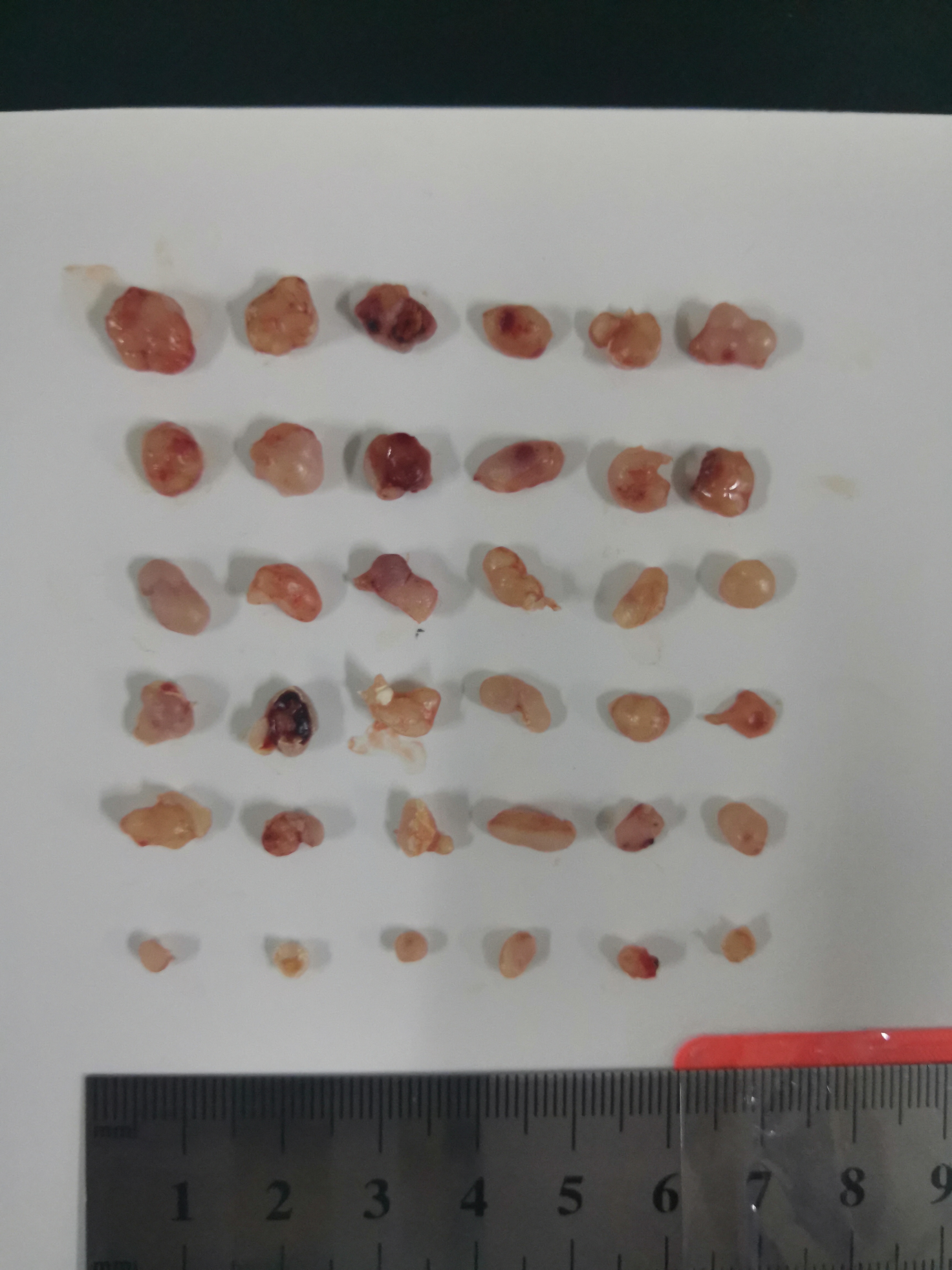

Supplement: Supplementary file 5 — Source data Fig. 3 [file 44321_2024_184_MOESM5_ESM.zip › Fig 3/Fig 3G/008a954b5e43adfdad3c4f7f1a9f11c.jpg]

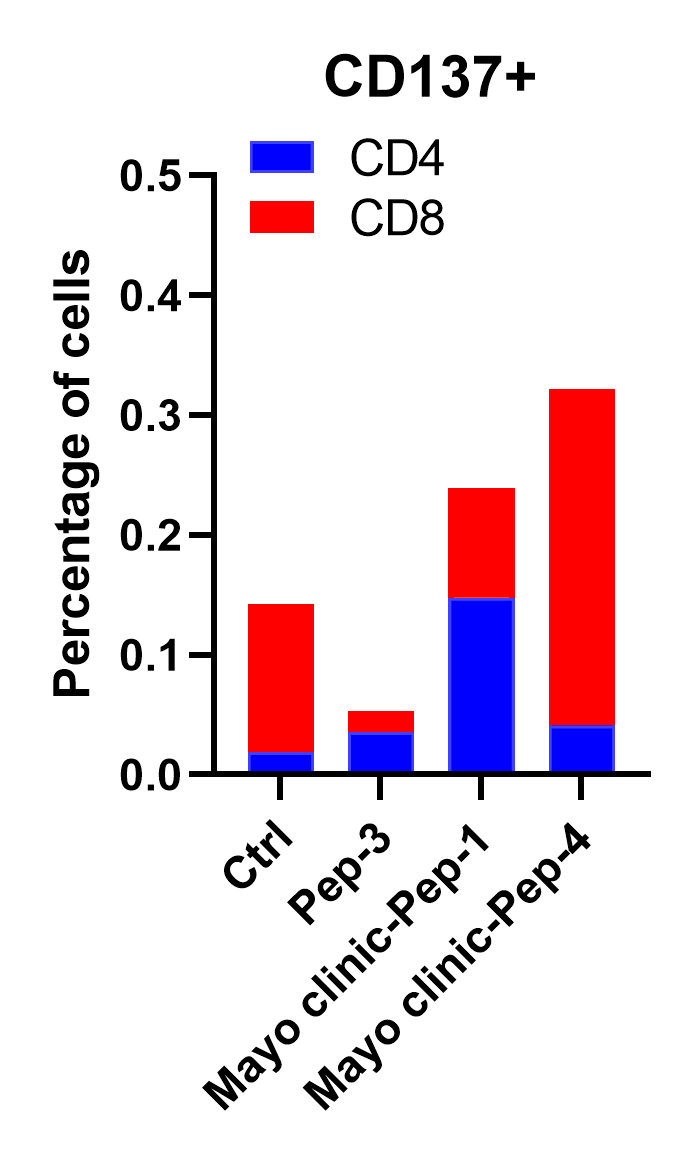

Supplement: Supplementary file 6 — Source data Fig. 4 [file 44321_2024_184_MOESM6_ESM.zip › Fig 4/Fig 4A-B/cd137.tif]

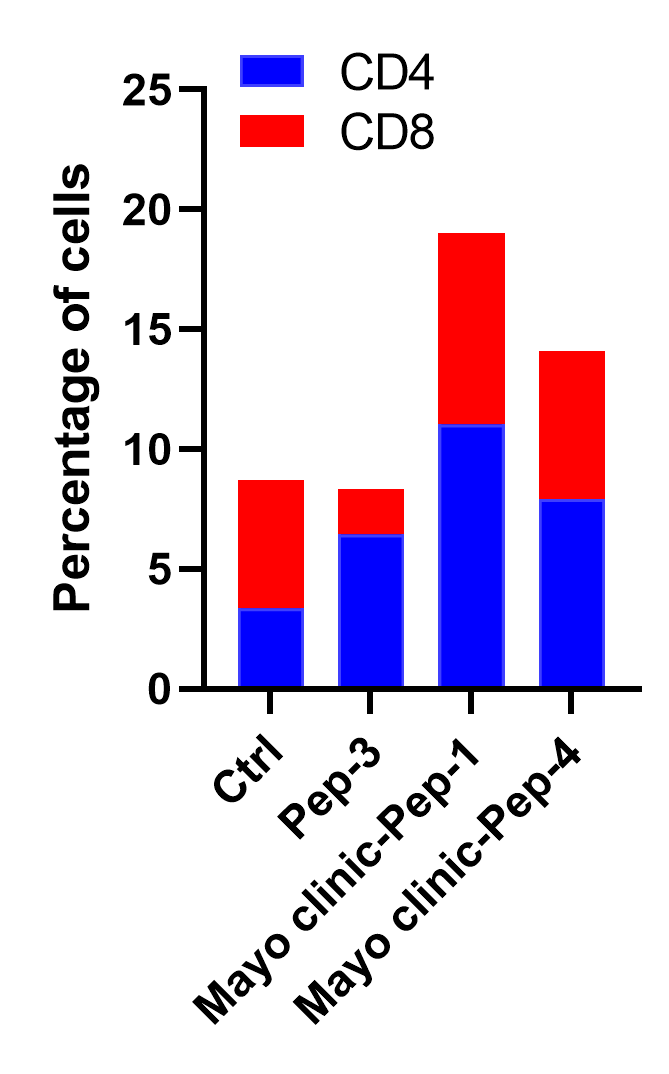

Supplement: Supplementary file 6 — Source data Fig. 4 [file 44321_2024_184_MOESM6_ESM.zip › Fig 4/Fig 4A-B/cd4 cd8.tif]

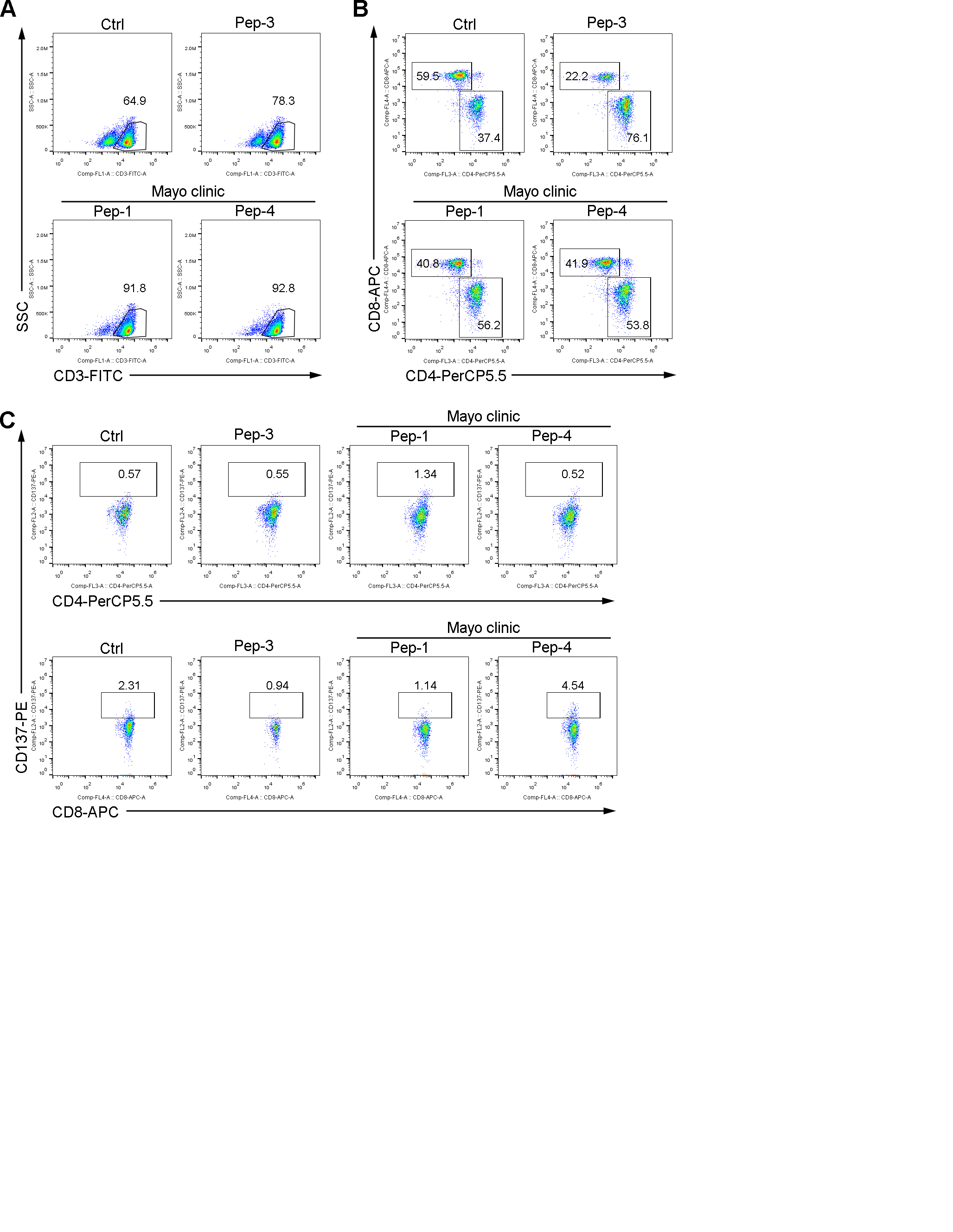

Supplement: Supplementary file 6 — Source data Fig. 4 [file 44321_2024_184_MOESM6_ESM.zip › Fig 4/Fig 4A-B/res.tif]

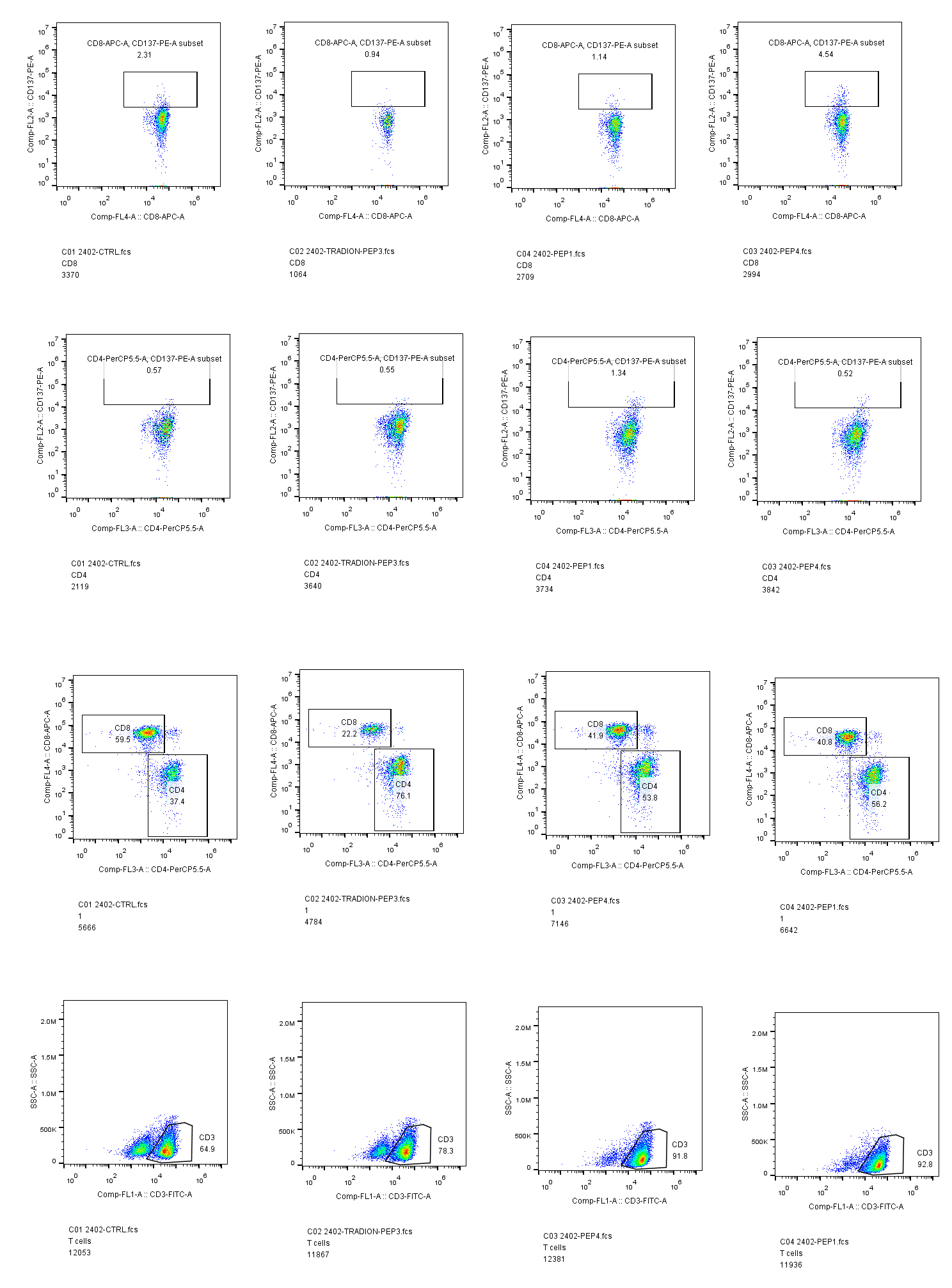

Supplement: Supplementary file 6 — Source data Fig. 4 [file 44321_2024_184_MOESM6_ESM.zip › Fig 4/Fig 4A-B/20201229-Layout.png]

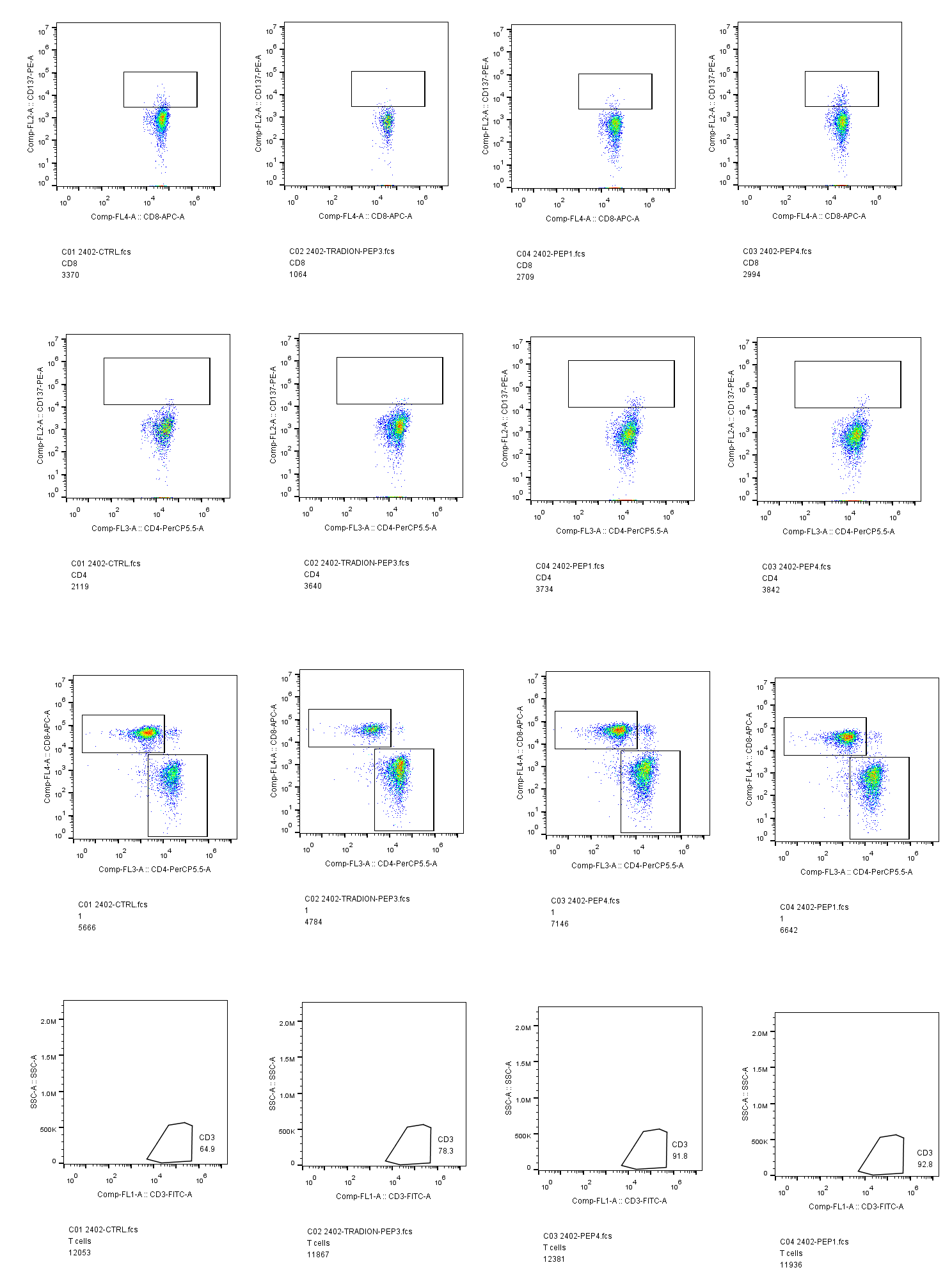

Supplement: Supplementary file 6 — Source data Fig. 4 [file 44321_2024_184_MOESM6_ESM.zip › Fig 4/Fig 4A-B/2.png]

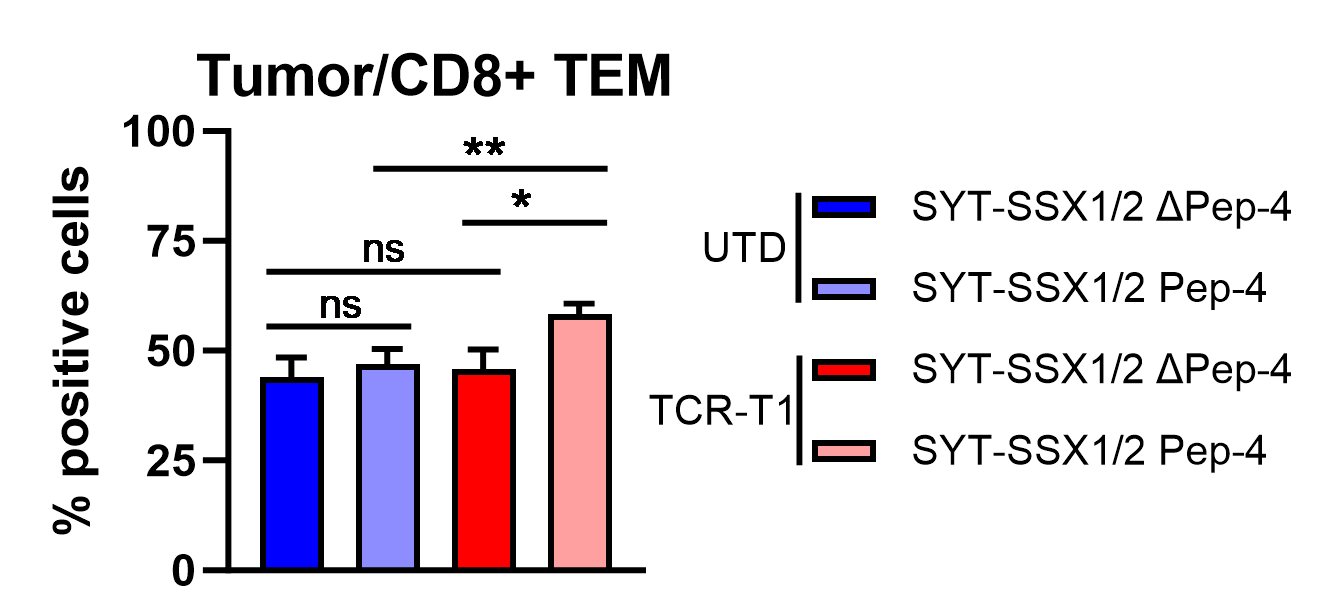

Supplement: Supplementary file 6 — Source data Fig. 4 [file 44321_2024_184_MOESM6_ESM.zip › Fig 4/Fig 4C-D/tumor cd8 tem.tif]

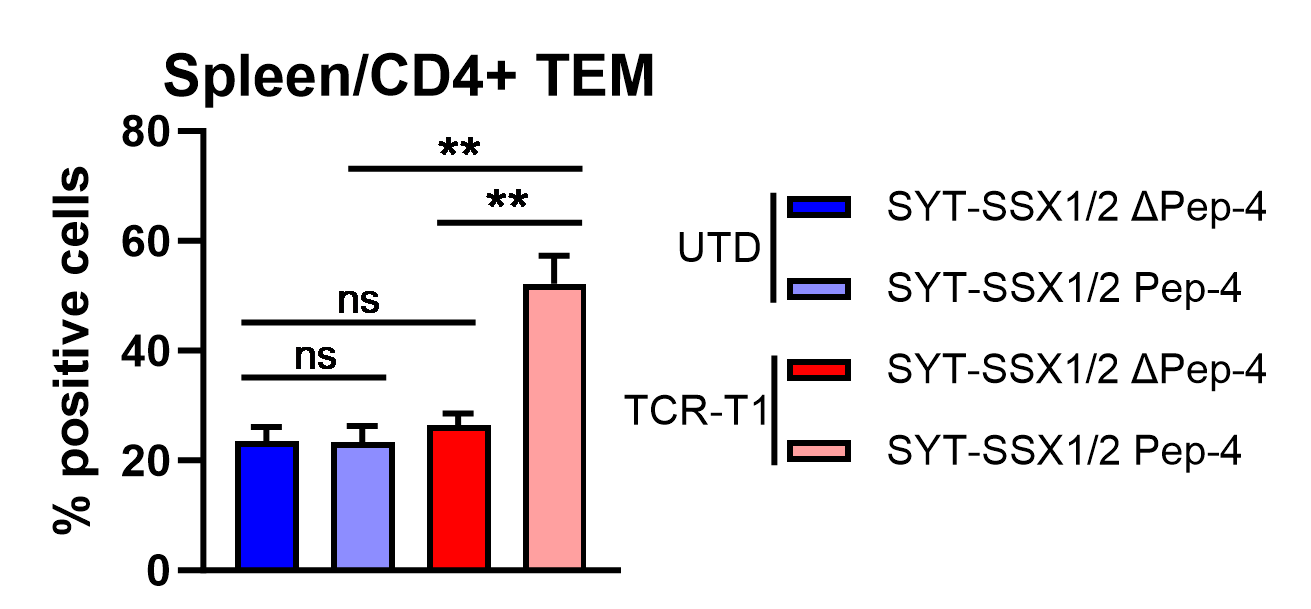

Supplement: Supplementary file 6 — Source data Fig. 4 [file 44321_2024_184_MOESM6_ESM.zip › Fig 4/Fig 4C-D/spleen cd4 tem.tif]

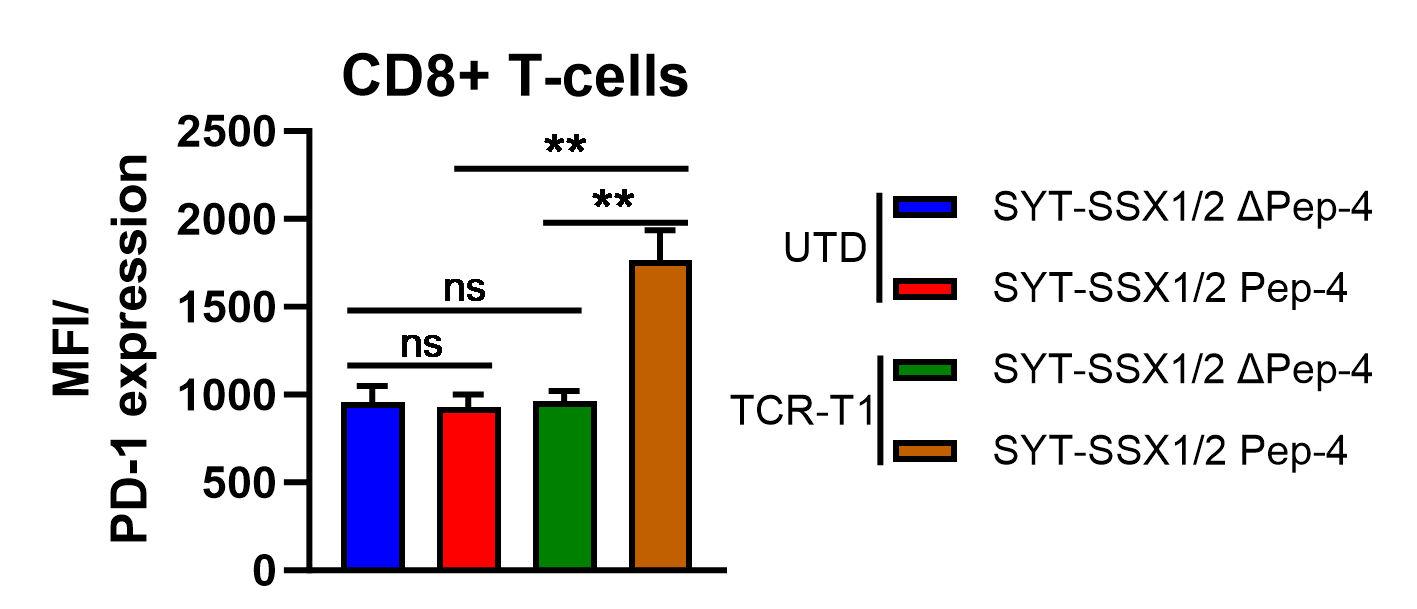

Supplement: Supplementary file 6 — Source data Fig. 4 [file 44321_2024_184_MOESM6_ESM.zip › Fig 4/Fig 4C-D/CD8 PD1.tif]

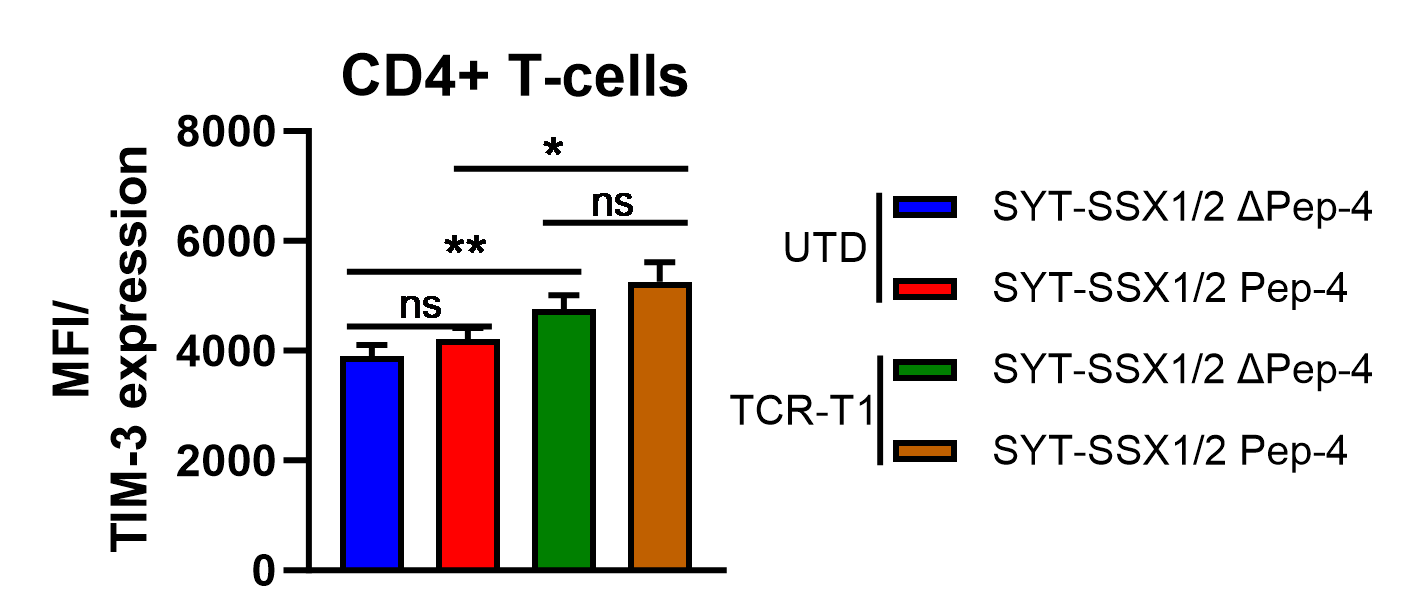

Supplement: Supplementary file 6 — Source data Fig. 4 [file 44321_2024_184_MOESM6_ESM.zip › Fig 4/Fig 4C-D/CD4 PD1.tif]

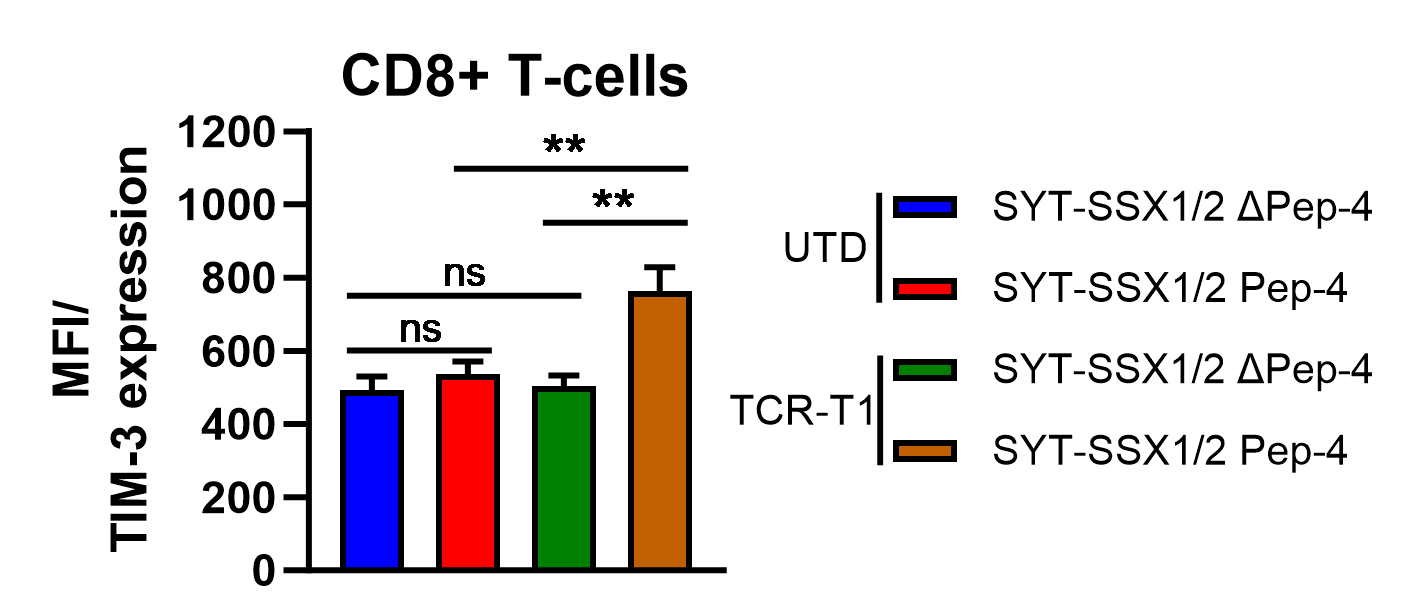

Supplement: Supplementary file 6 — Source data Fig. 4 [file 44321_2024_184_MOESM6_ESM.zip › Fig 4/Fig 4C-D/cd8 tim3.tif]

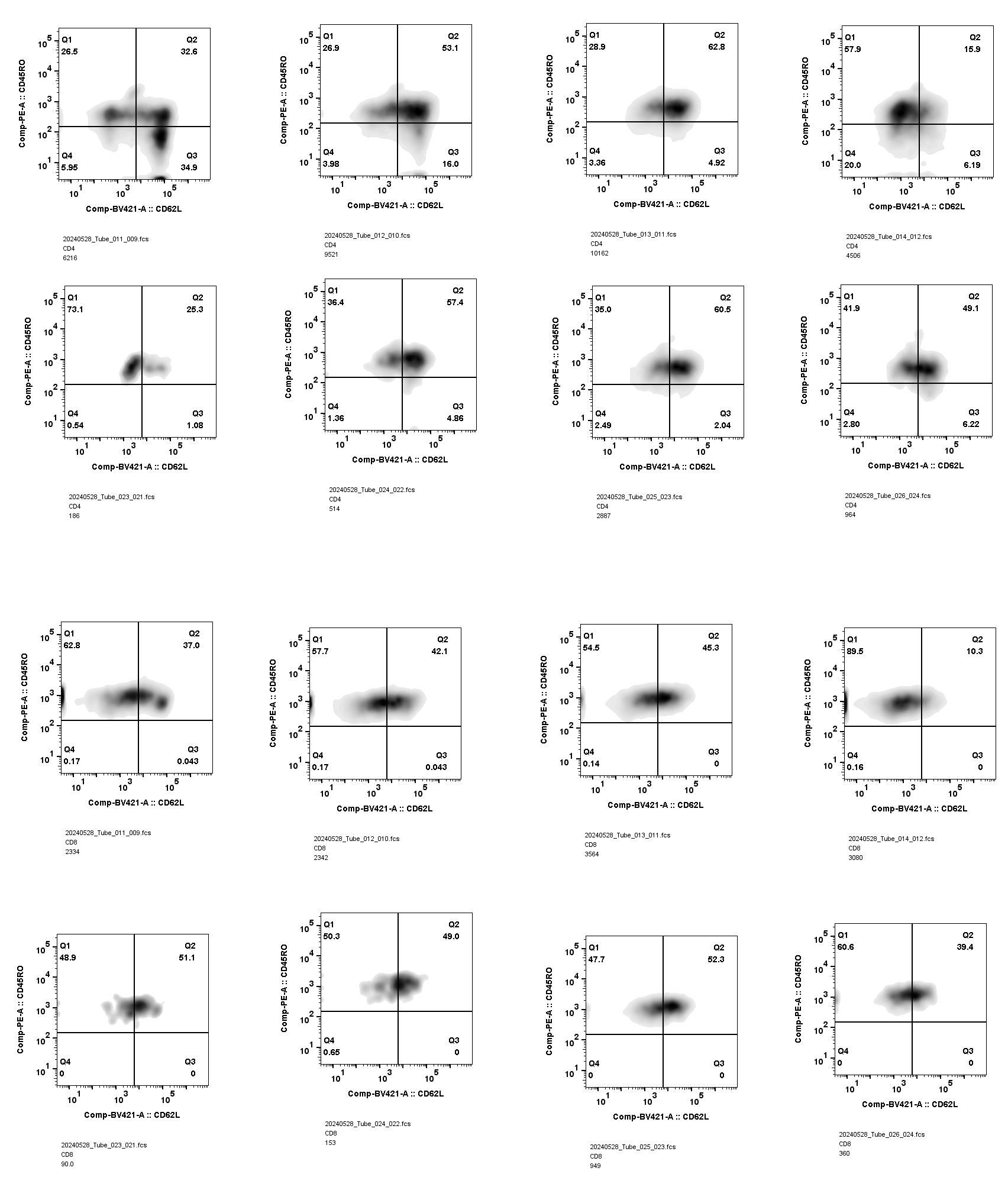

Supplement: Supplementary file 6 — Source data Fig. 4 [file 44321_2024_184_MOESM6_ESM.zip › Fig 4/Fig 4C-D/25-Jul-2024-Layout.png]

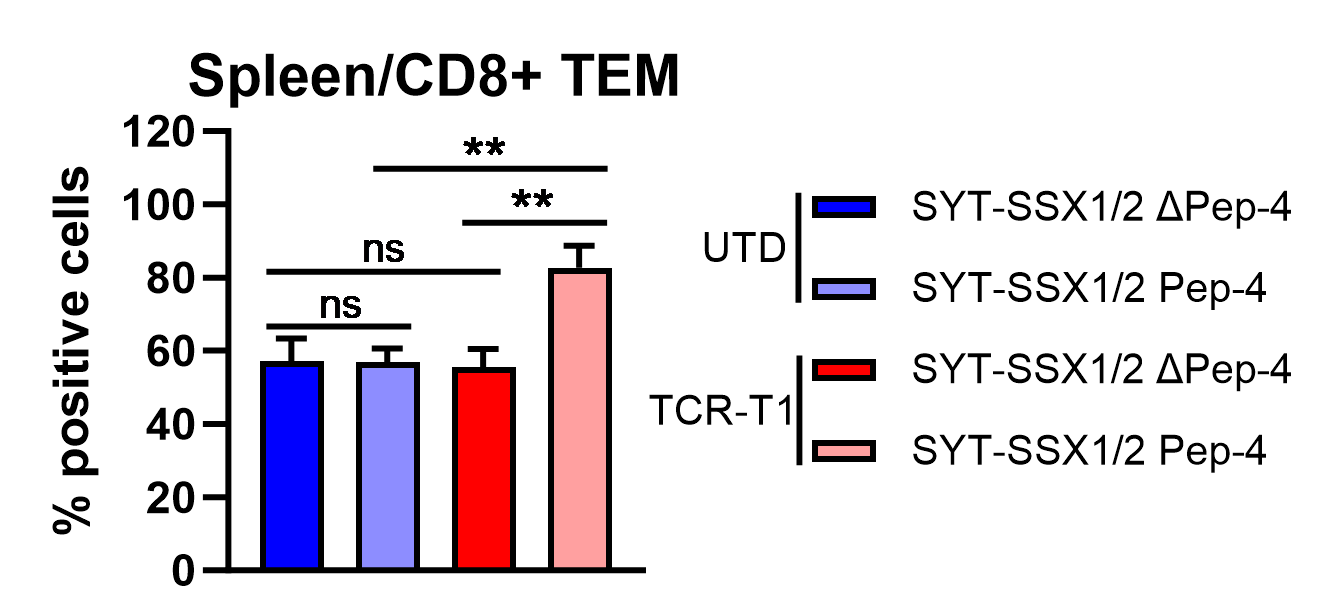

Supplement: Supplementary file 6 — Source data Fig. 4 [file 44321_2024_184_MOESM6_ESM.zip › Fig 4/Fig 4C-D/spleen cd8 tem.tif]

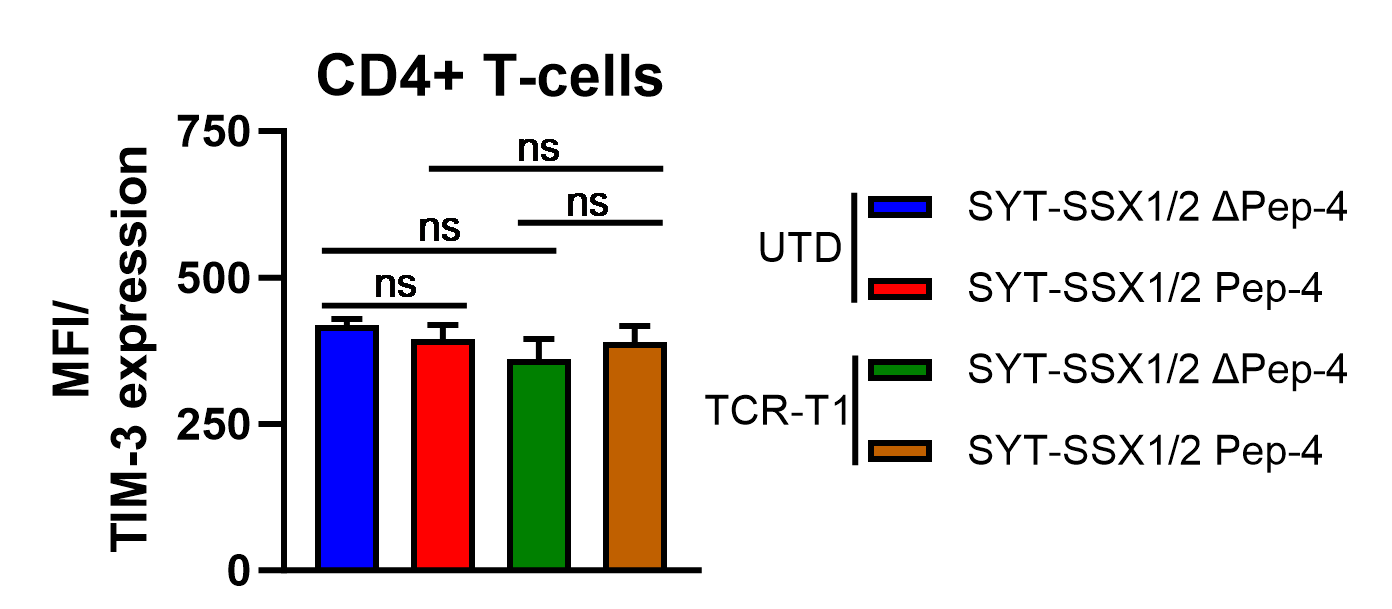

Supplement: Supplementary file 6 — Source data Fig. 4 [file 44321_2024_184_MOESM6_ESM.zip › Fig 4/Fig 4C-D/cd4 tim3.tif]

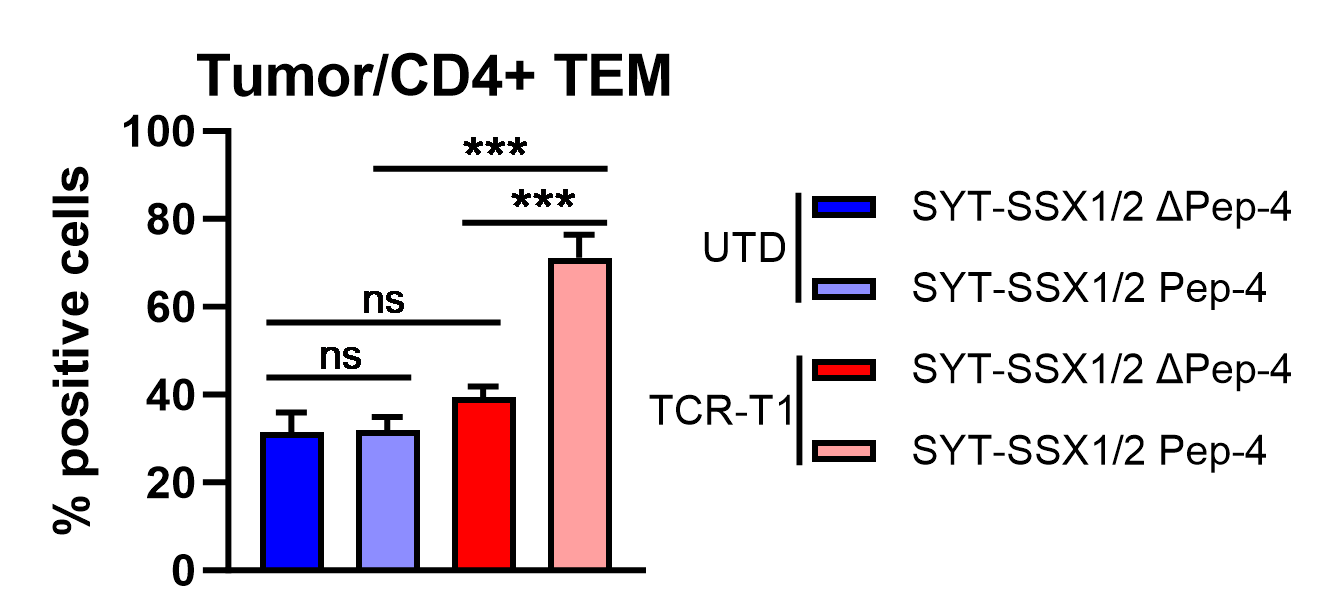

Supplement: Supplementary file 6 — Source data Fig. 4 [file 44321_2024_184_MOESM6_ESM.zip › Fig 4/Fig 4C-D/tumor cd4 tem.tif]

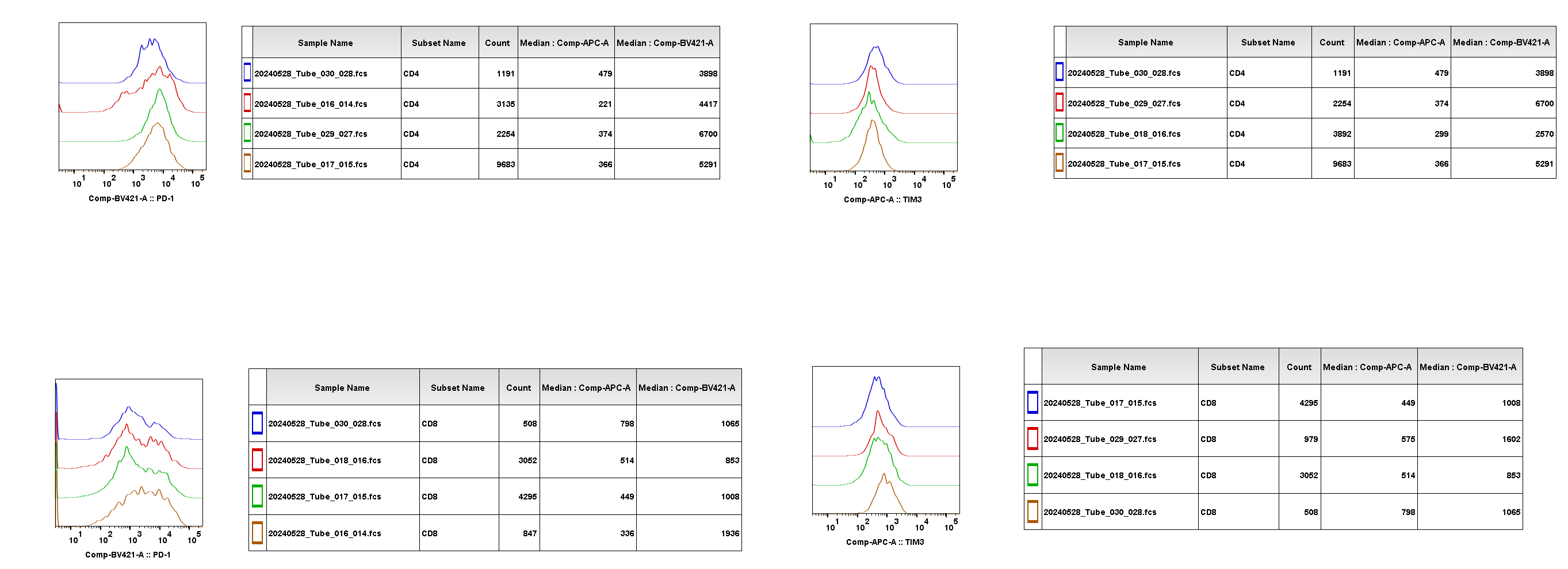

Supplement: Supplementary file 6 — Source data Fig. 4 [file 44321_2024_184_MOESM6_ESM.zip › Fig 4/Fig 4E-F/25-Jul-2024-Layout.png]

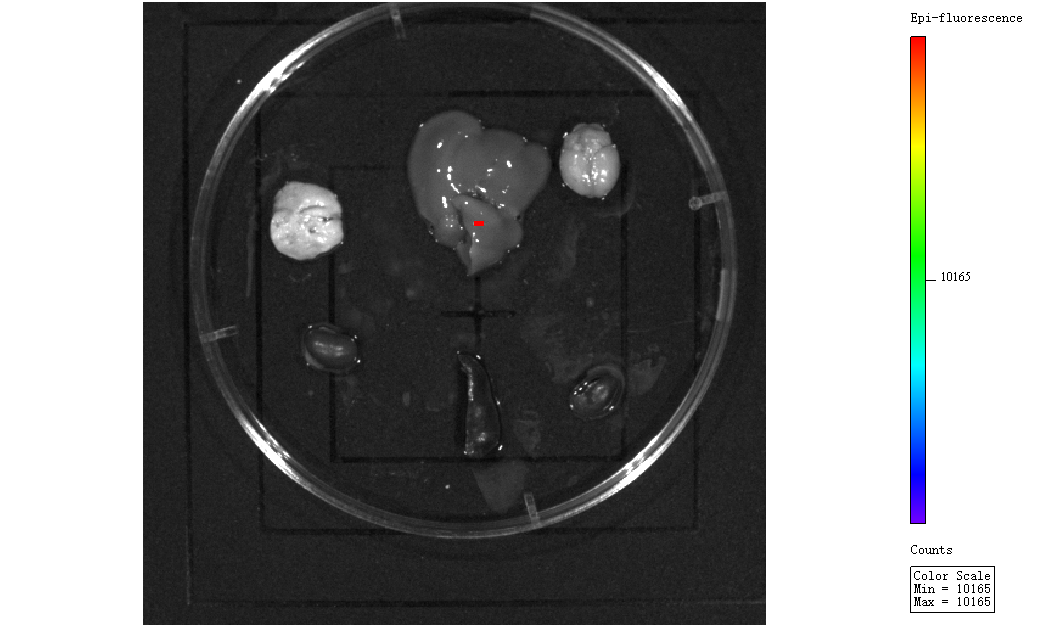

Supplement: Supplementary file 7 — Source data Fig. 5 [file 44321_2024_184_MOESM7_ESM.zip › Fig 5/Fig 5F/1233.png]

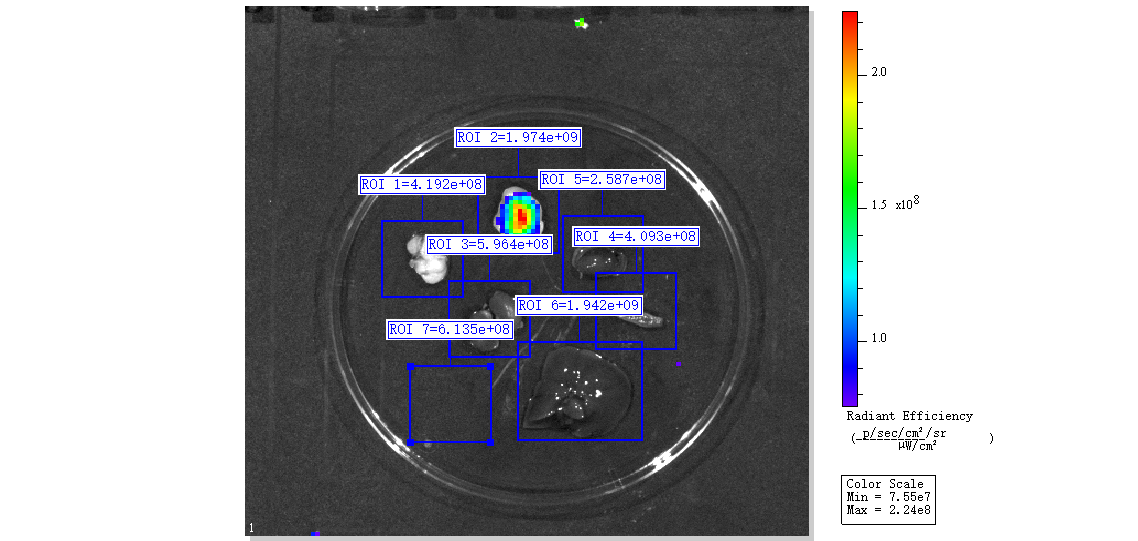

Supplement: Supplementary file 7 — Source data Fig. 5 [file 44321_2024_184_MOESM7_ESM.zip › Fig 5/Fig 5F/123.png]

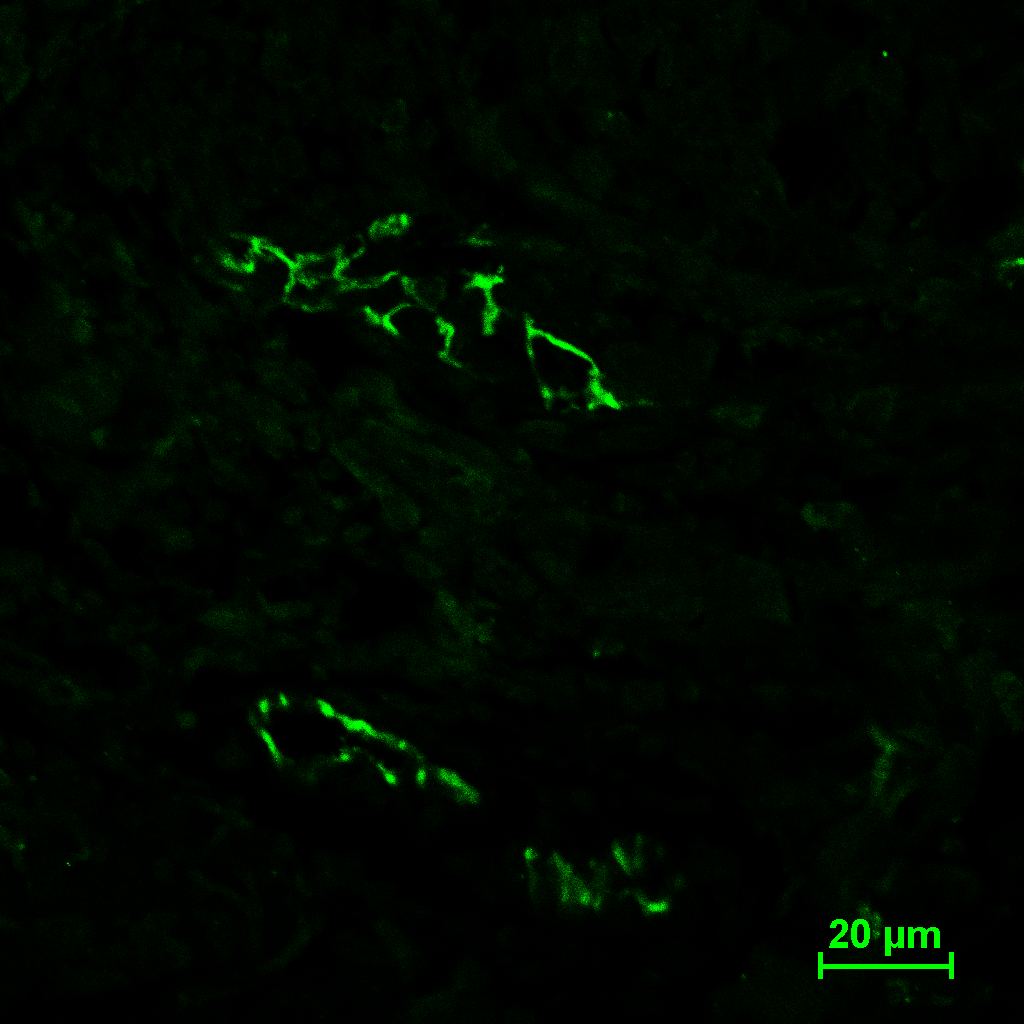

Supplement: Supplementary file 7 — Source data Fig. 5 [file 44321_2024_184_MOESM7_ESM.zip › Fig 5/Fig 5G/40-2c2.tif]

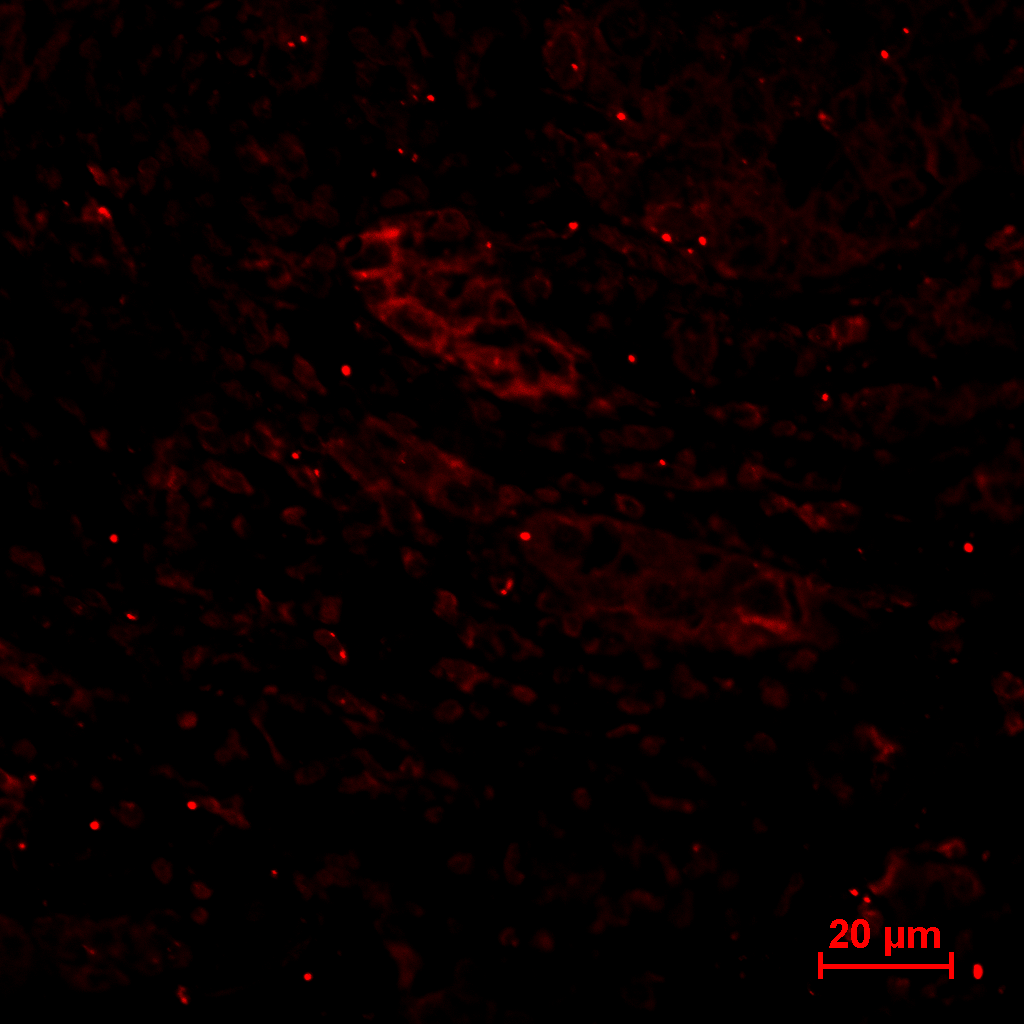

Supplement: Supplementary file 7 — Source data Fig. 5 [file 44321_2024_184_MOESM7_ESM.zip › Fig 5/Fig 5G/40-2c3.tif]

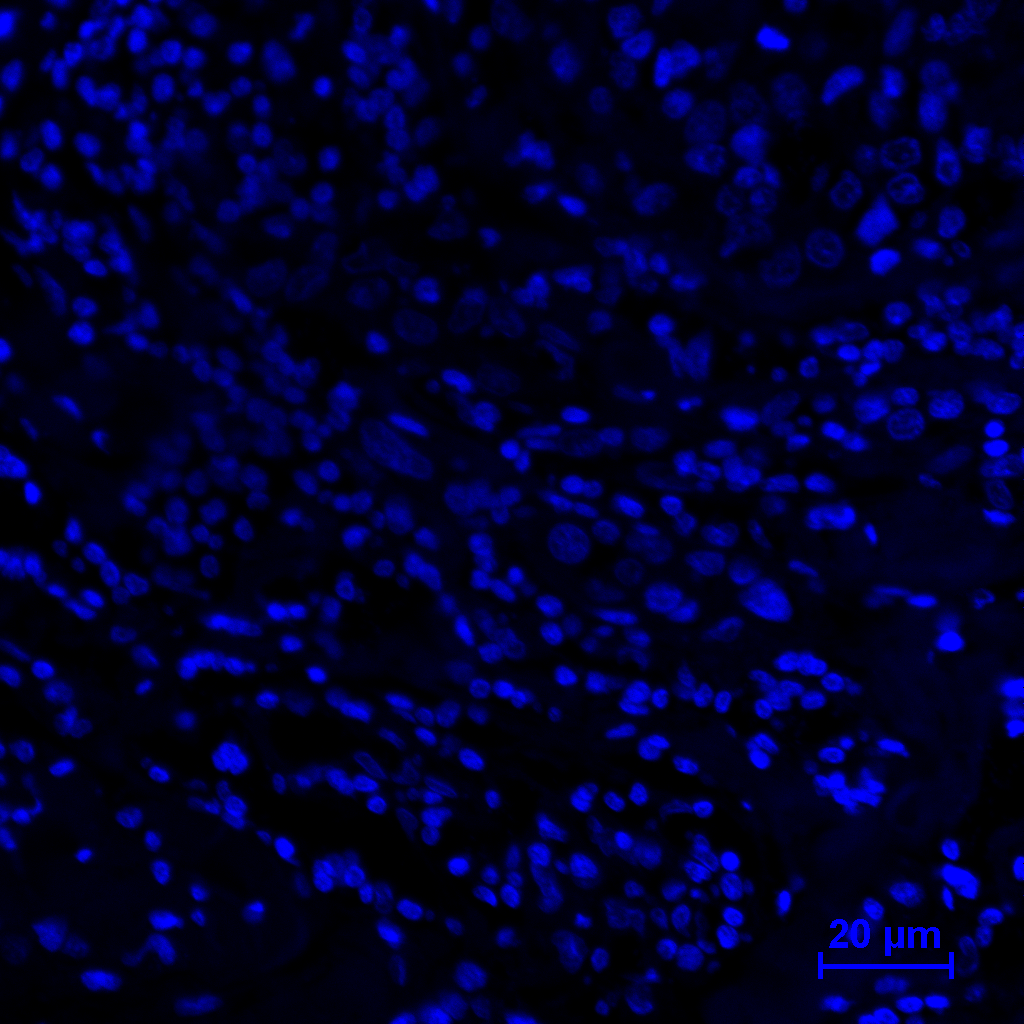

Supplement: Supplementary file 7 — Source data Fig. 5 [file 44321_2024_184_MOESM7_ESM.zip › Fig 5/Fig 5G/40-2c1.tif]

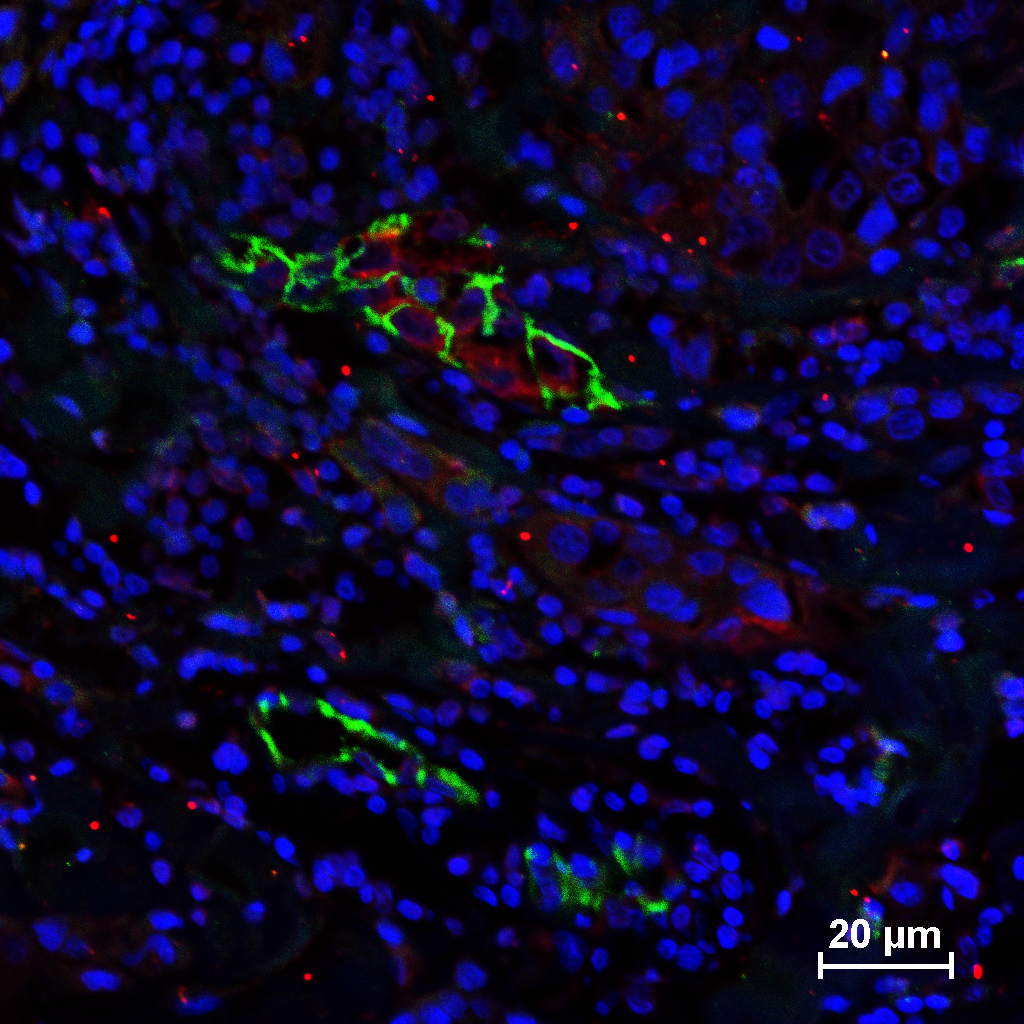

Supplement: Supplementary file 7 — Source data Fig. 5 [file 44321_2024_184_MOESM7_ESM.zip › Fig 5/Fig 5G/40-2.tif]

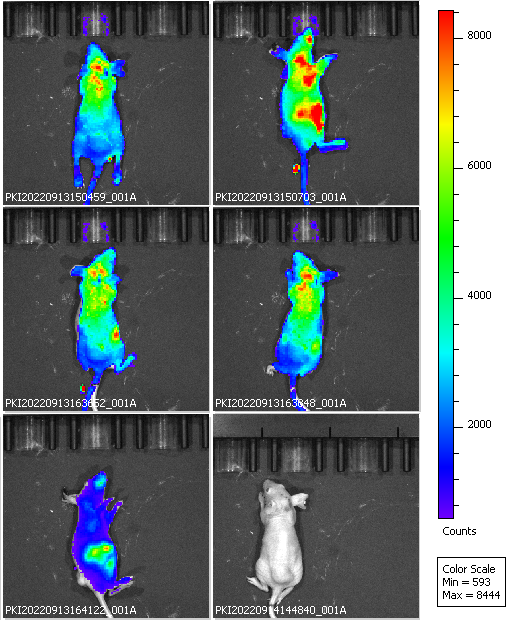

Supplement: Supplementary file 7 — Source data Fig. 5 [file 44321_2024_184_MOESM7_ESM.zip › Fig 5/Fig 5E/μ£¬μáçΘóÿ-1.tif]

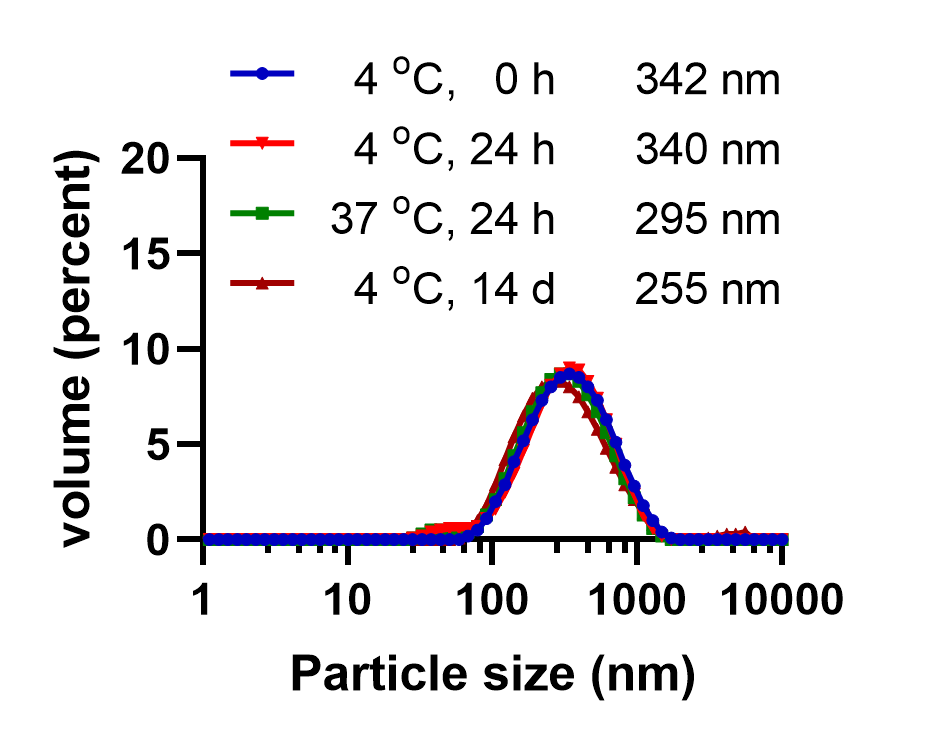

Supplement: Supplementary file 7 — Source data Fig. 5 [file 44321_2024_184_MOESM7_ESM.zip › Fig 5/Fig 5B/size.tif]

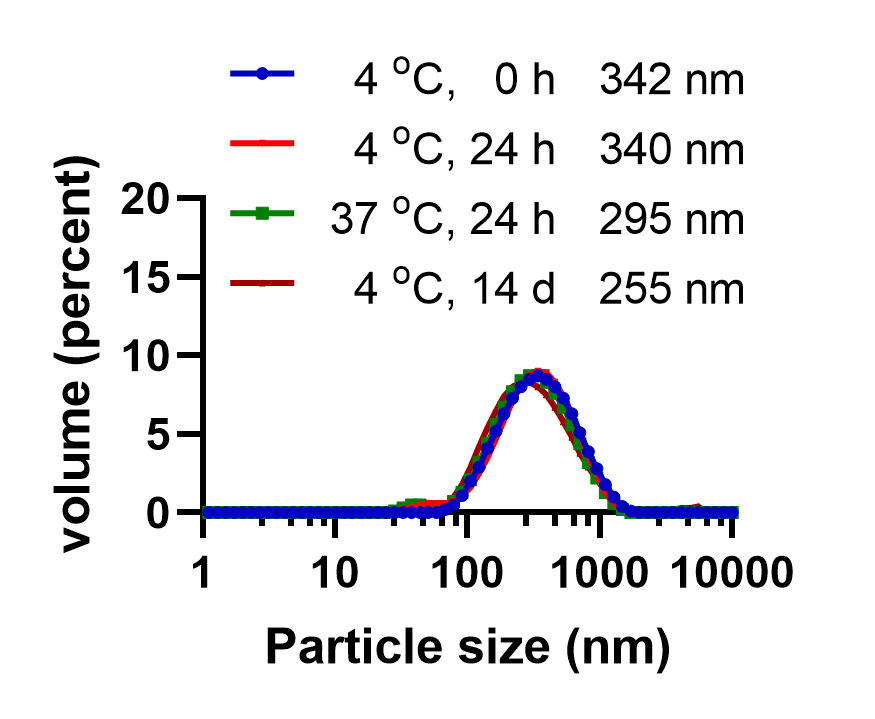

Supplement: Supplementary file 7 — Source data Fig. 5 [file 44321_2024_184_MOESM7_ESM.zip › Fig 5/Fig 5B/212.tif]

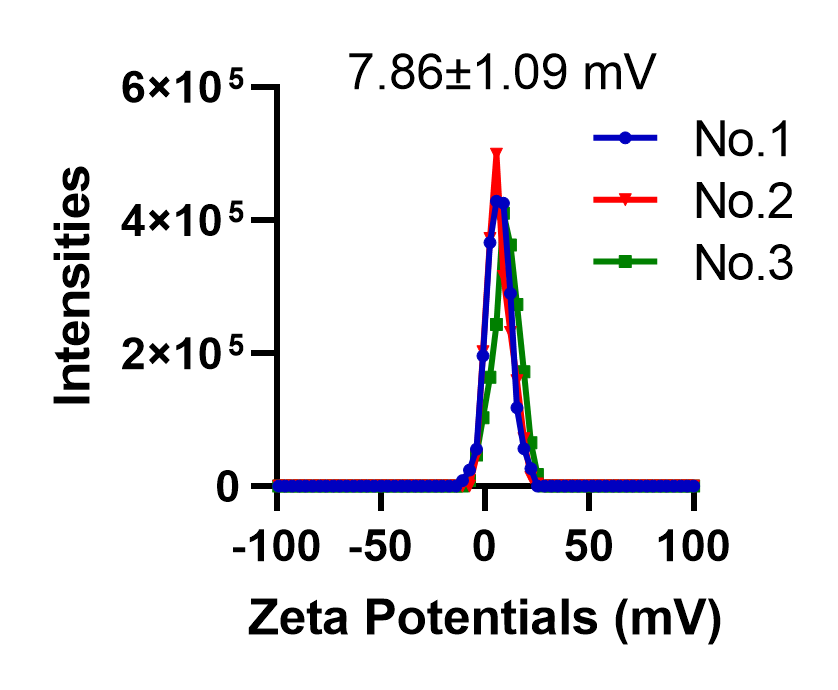

Supplement: Supplementary file 7 — Source data Fig. 5 [file 44321_2024_184_MOESM7_ESM.zip › Fig 5/Fig 5B/211.tif]

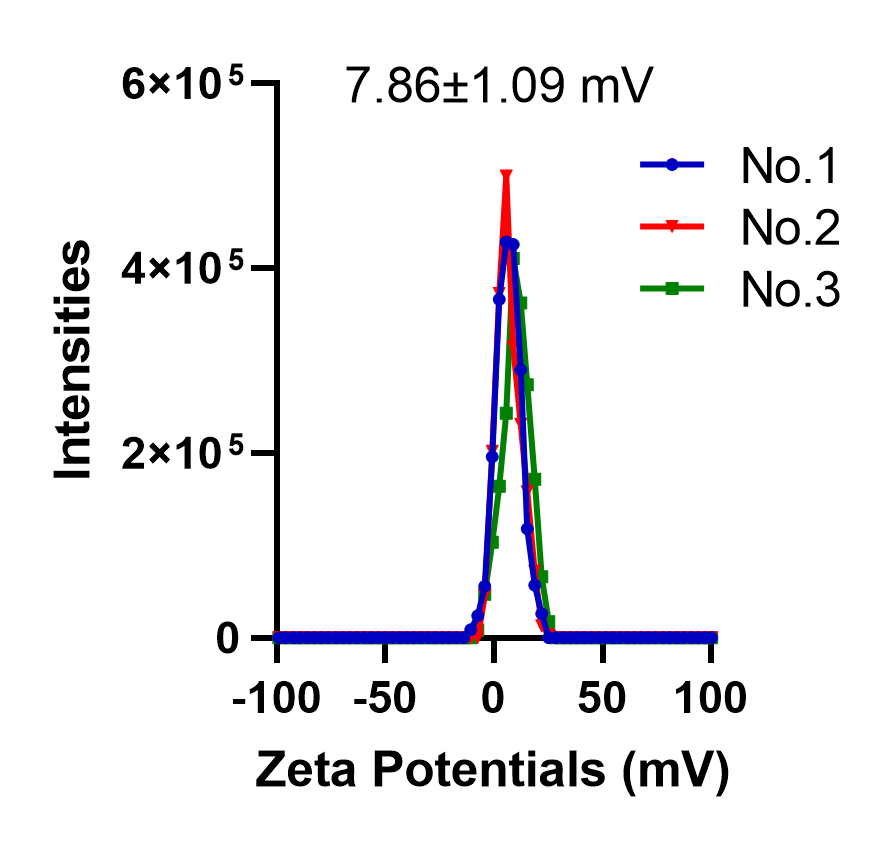

Supplement: Supplementary file 7 — Source data Fig. 5 [file 44321_2024_184_MOESM7_ESM.zip › Fig 5/Fig 5B/2122.tif]

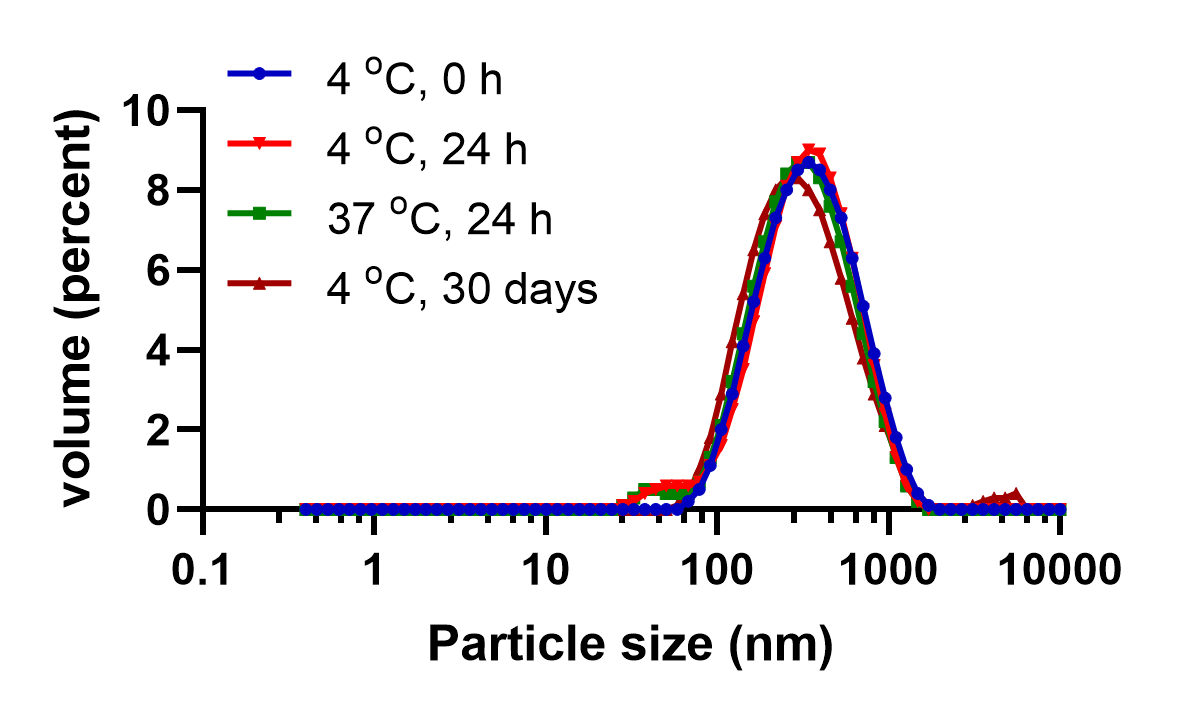

Supplement: Supplementary file 7 — Source data Fig. 5 [file 44321_2024_184_MOESM7_ESM.zip › Fig 5/Fig 5B/stability.tif]

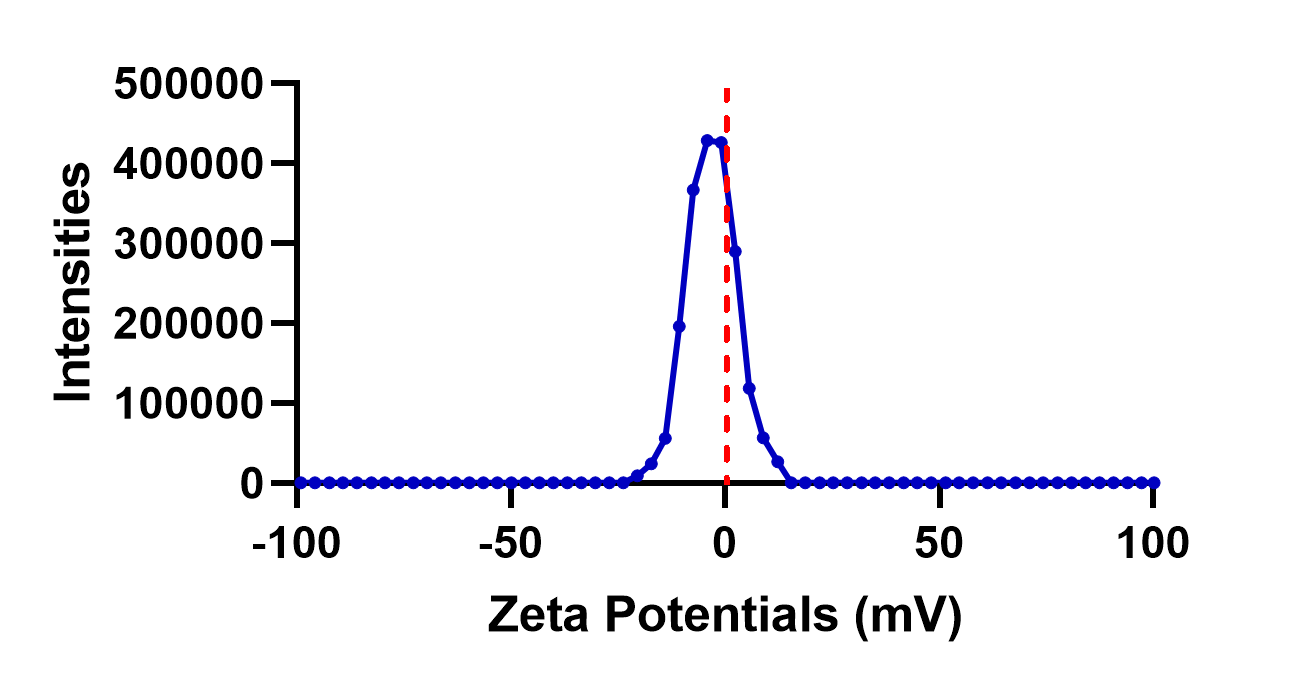

Supplement: Supplementary file 7 — Source data Fig. 5 [file 44321_2024_184_MOESM7_ESM.zip › Fig 5/Fig 5C/Zeta Potentials.tif]

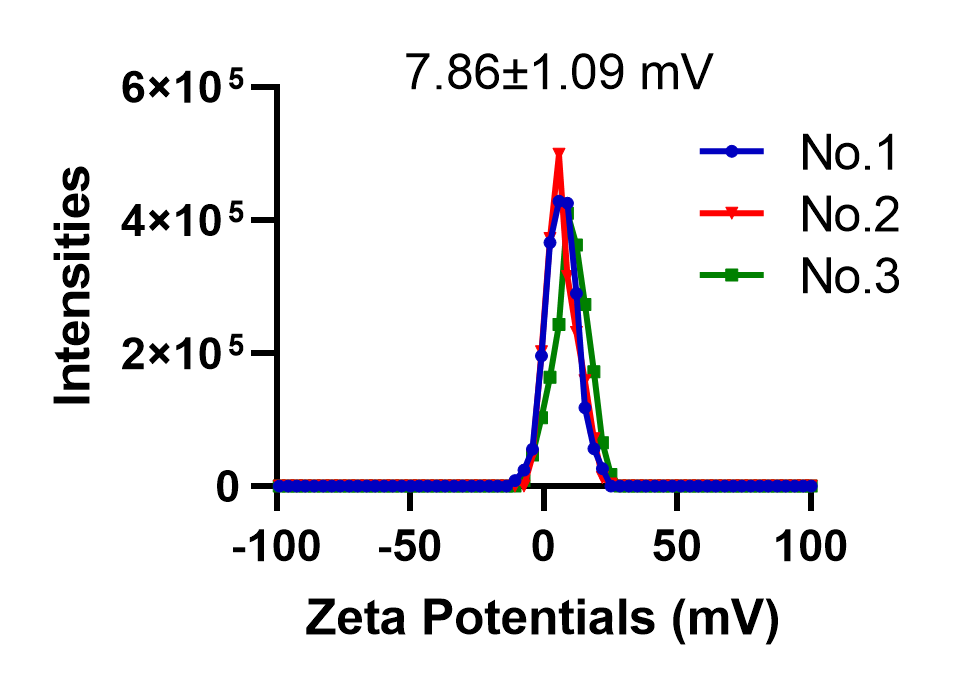

Supplement: Supplementary file 7 — Source data Fig. 5 [file 44321_2024_184_MOESM7_ESM.zip › Fig 5/Fig 5C/zeta.tif]

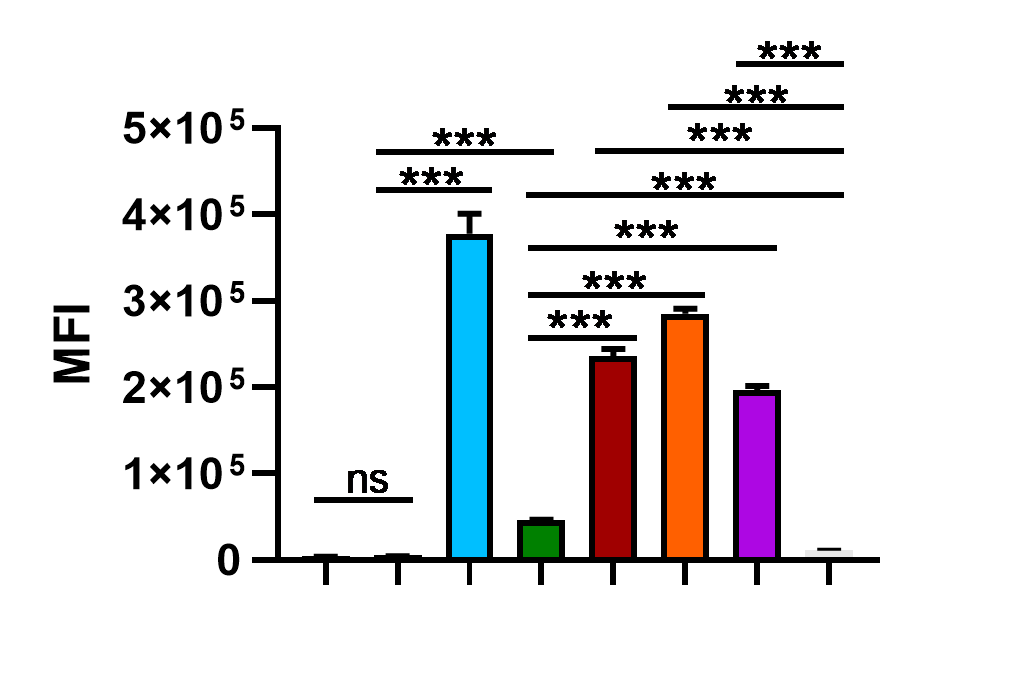

Supplement: Supplementary file 7 — Source data Fig. 5 [file 44321_2024_184_MOESM7_ESM.zip › Fig 5/Fig 5D/res 123.tif]

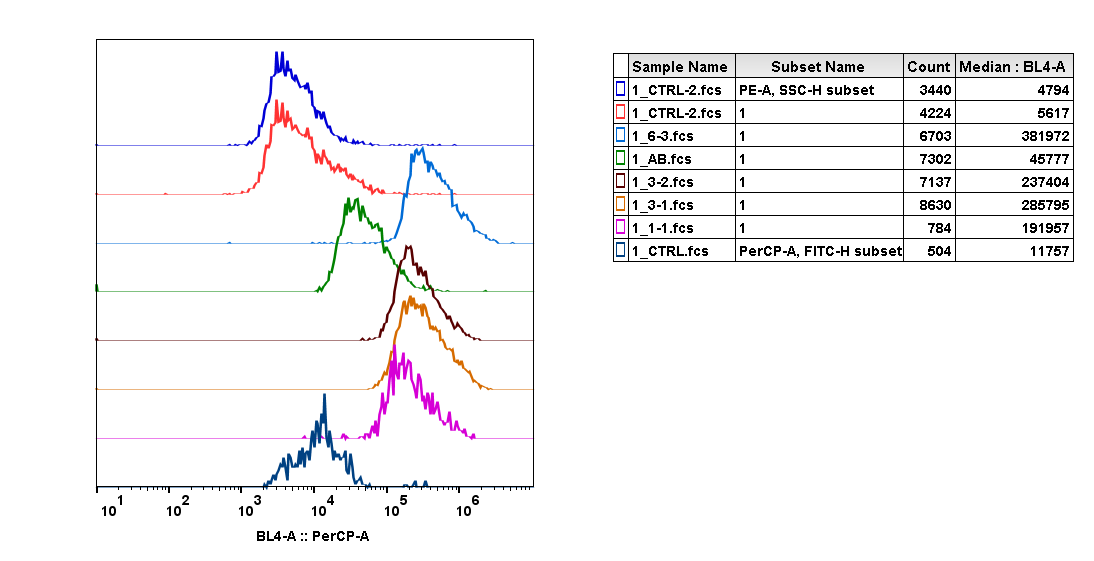

Supplement: Supplementary file 7 — Source data Fig. 5 [file 44321_2024_184_MOESM7_ESM.zip › Fig 5/Fig 5D/28-Jun-2023-Layout-1.png]

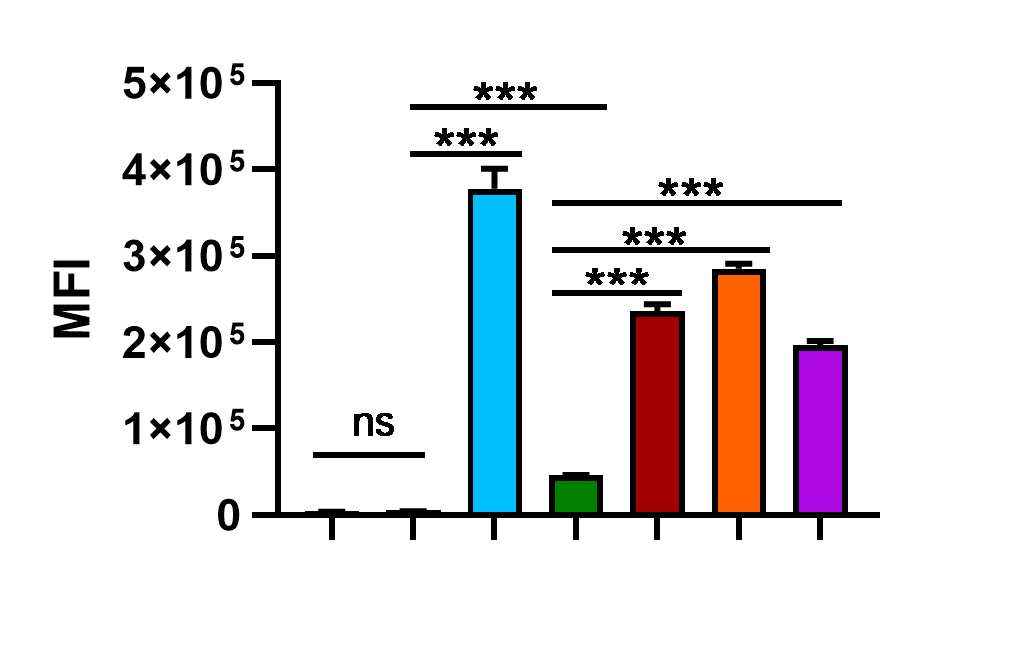

Supplement: Supplementary file 7 — Source data Fig. 5 [file 44321_2024_184_MOESM7_ESM.zip › Fig 5/Fig 5D/res1.tif]

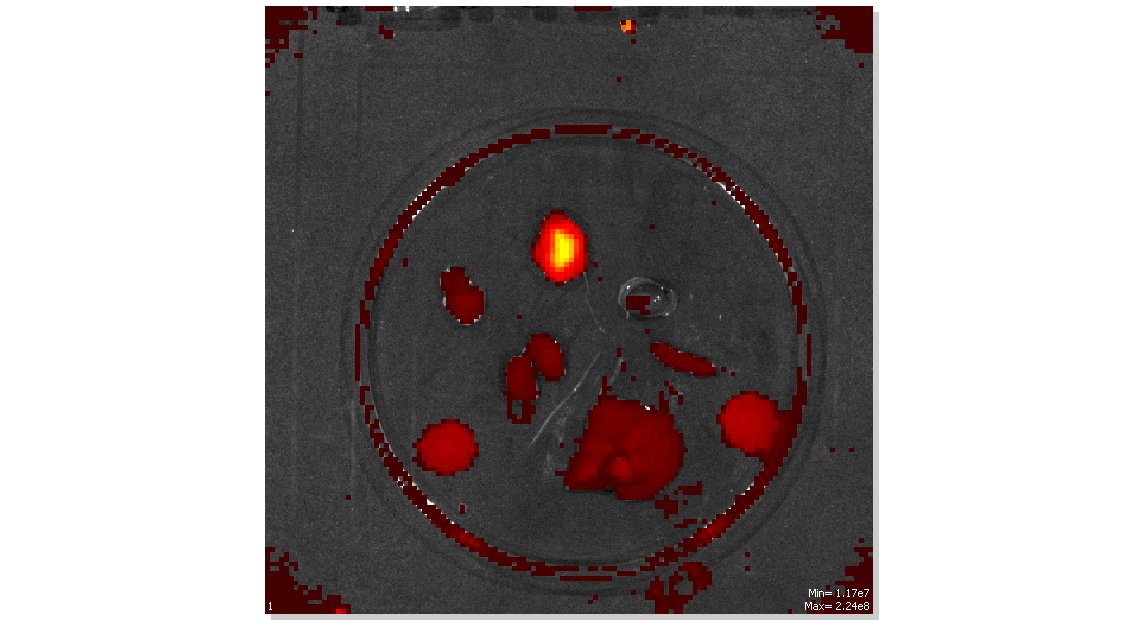

Supplement: Supplementary file 7 — Source data Fig. 5 [file 44321_2024_184_MOESM7_ESM.zip › Fig 5/Fig 5F/PKI20230323141222_SEQ/PKI20230323141222_SEQ.PNG]

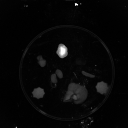

Supplement: Supplementary file 7 — Source data Fig. 5 [file 44321_2024_184_MOESM7_ESM.zip › Fig 5/Fig 5F/PKI20230323141222_SEQ/PKI20230323141222_001/luminescent.TIF]

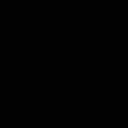

Supplement: Supplementary file 7 — Source data Fig. 5 [file 44321_2024_184_MOESM7_ESM.zip › Fig 5/Fig 5F/PKI20230323141222_SEQ/PKI20230323141222_001/readbiasonly.TIF]

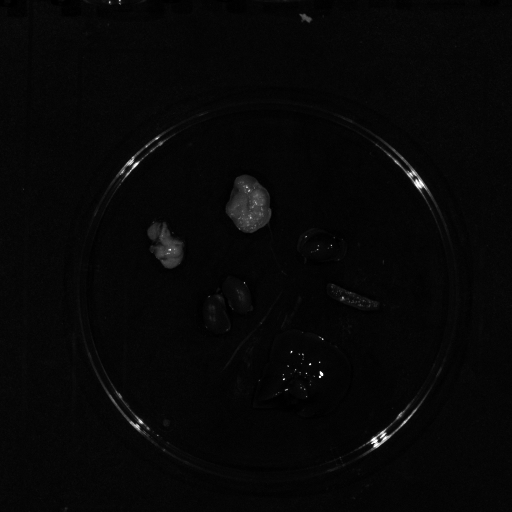

Supplement: Supplementary file 7 — Source data Fig. 5 [file 44321_2024_184_MOESM7_ESM.zip › Fig 5/Fig 5F/PKI20230323141222_SEQ/PKI20230323141222_001/photograph.TIF]

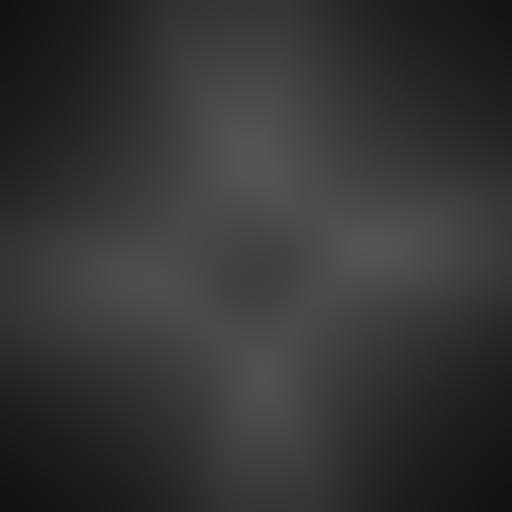

Supplement: Supplementary file 7 — Source data Fig. 5 [file 44321_2024_184_MOESM7_ESM.zip › Fig 5/Fig 5F/PKI20230323141222_SEQ/PKI20230323141222_001/fluorescentreference.tif]

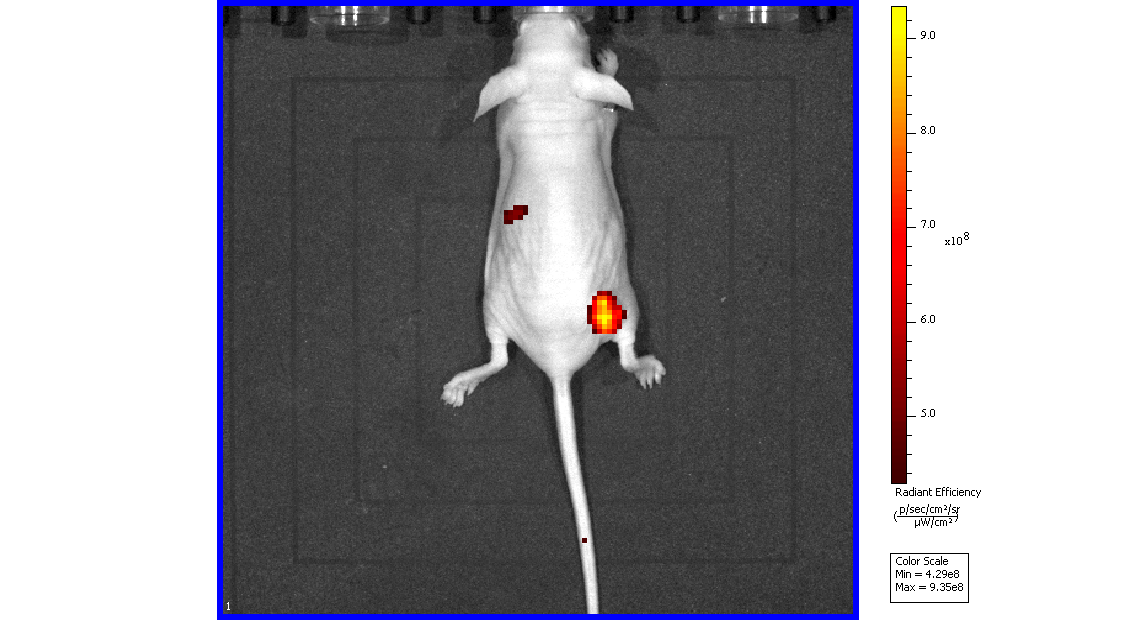

Supplement: Supplementary file 7 — Source data Fig. 5 [file 44321_2024_184_MOESM7_ESM.zip › Fig 5/Fig 5E/20221130/36h/PKI20221201131813_SEQ/PKI20221201131813_SEQ.PNG]

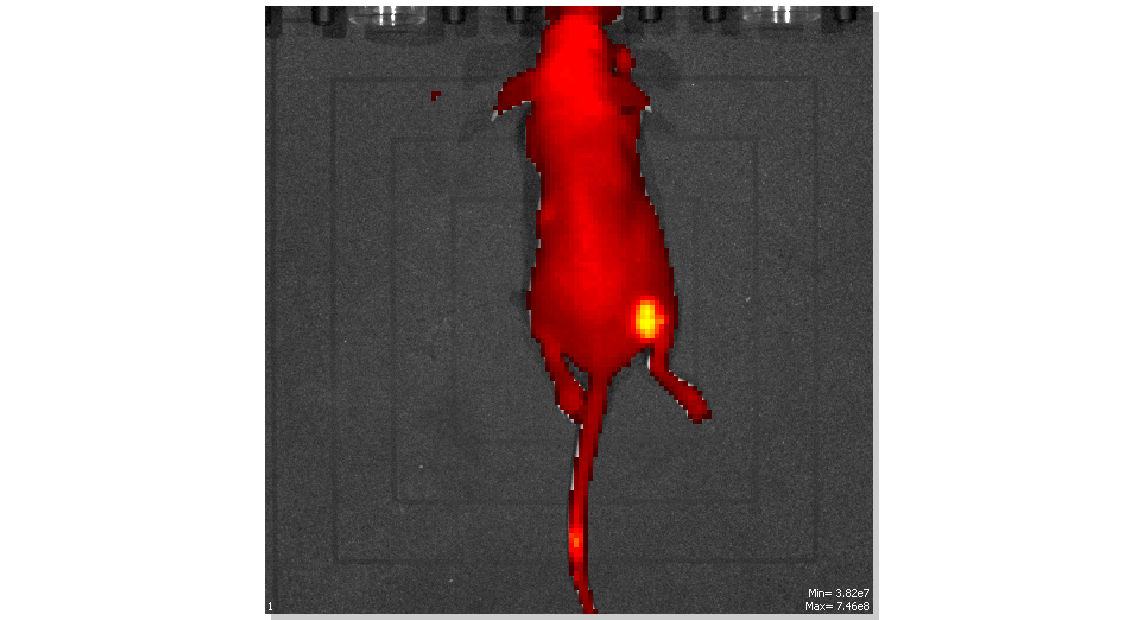

Supplement: Supplementary file 7 — Source data Fig. 5 [file 44321_2024_184_MOESM7_ESM.zip › Fig 5/Fig 5E/20221130/12 h/PKI20221130134430_SEQ/PKI20221130134430_SEQ.PNG]

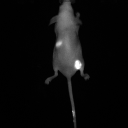

Supplement: Supplementary file 7 — Source data Fig. 5 [file 44321_2024_184_MOESM7_ESM.zip › Fig 5/Fig 5E/20221130/36h/PKI20221201131813_SEQ/PKI20221201131813_001/luminescent.TIF]

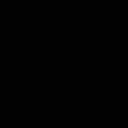

Supplement: Supplementary file 7 — Source data Fig. 5 [file 44321_2024_184_MOESM7_ESM.zip › Fig 5/Fig 5E/20221130/36h/PKI20221201131813_SEQ/PKI20221201131813_001/readbiasonly.TIF]

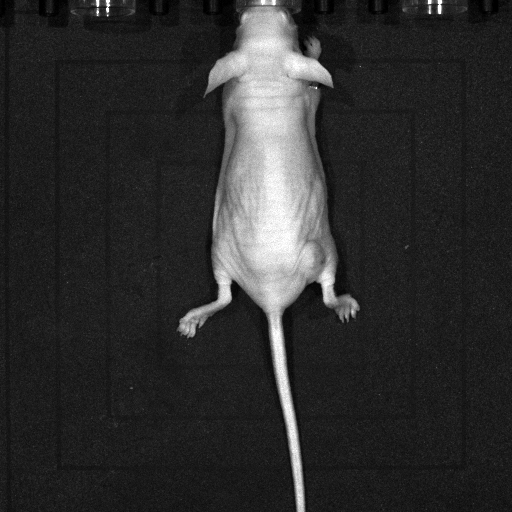

Supplement: Supplementary file 7 — Source data Fig. 5 [file 44321_2024_184_MOESM7_ESM.zip › Fig 5/Fig 5E/20221130/36h/PKI20221201131813_SEQ/PKI20221201131813_001/photograph.TIF]

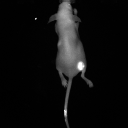

Supplement: Supplementary file 7 — Source data Fig. 5 [file 44321_2024_184_MOESM7_ESM.zip › Fig 5/Fig 5E/20221130/12 h/PKI20221130134430_SEQ/PKI20221130134430_001/luminescent.TIF]

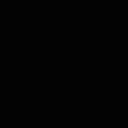

Supplement: Supplementary file 7 — Source data Fig. 5 [file 44321_2024_184_MOESM7_ESM.zip › Fig 5/Fig 5E/20221130/12 h/PKI20221130134430_SEQ/PKI20221130134430_001/readbiasonly.TIF]

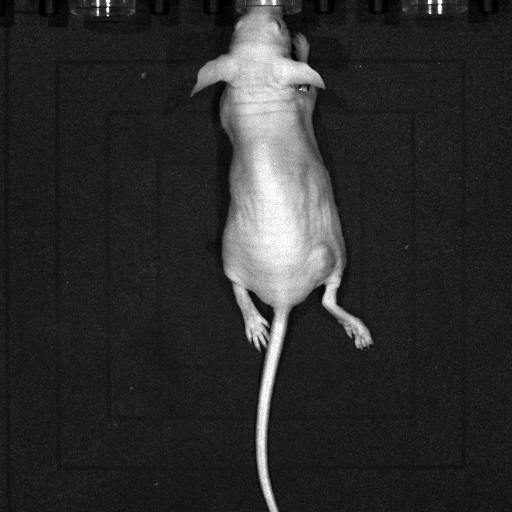

Supplement: Supplementary file 7 — Source data Fig. 5 [file 44321_2024_184_MOESM7_ESM.zip › Fig 5/Fig 5E/20221130/12 h/PKI20221130134430_SEQ/PKI20221130134430_001/photograph.TIF]

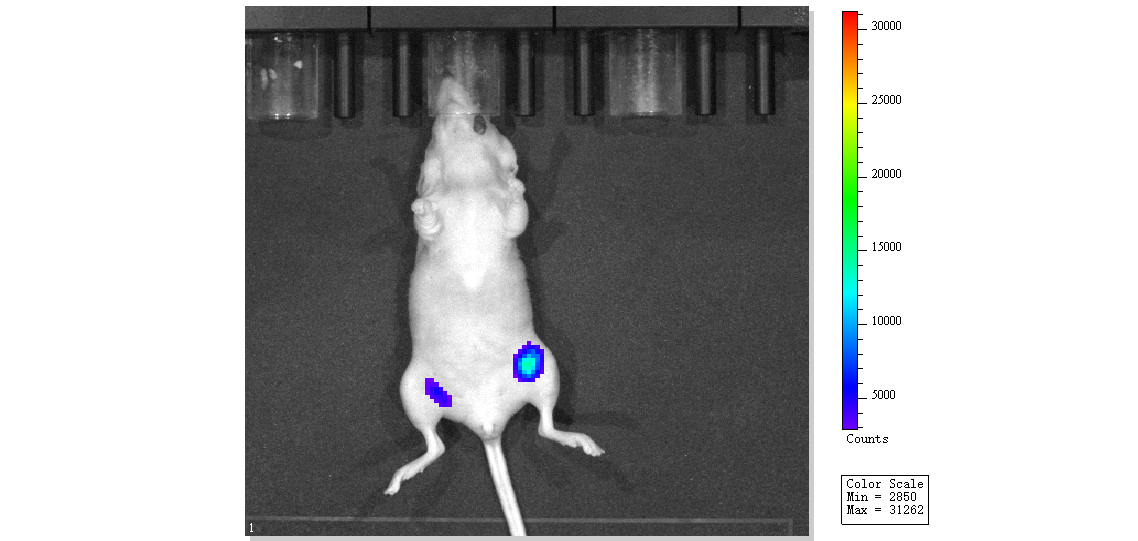

Supplement: Supplementary file 8 — Source data Fig. 6 [file 44321_2024_184_MOESM8_ESM.zip › Fig 6/Fig 6F/14.png]

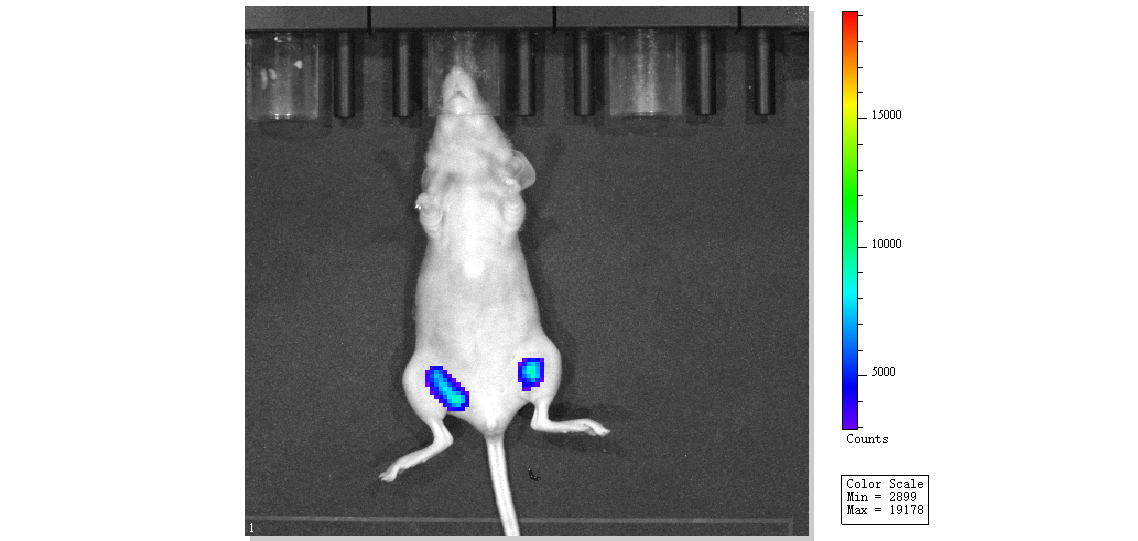

Supplement: Supplementary file 8 — Source data Fig. 6 [file 44321_2024_184_MOESM8_ESM.zip › Fig 6/Fig 6F/15.png]

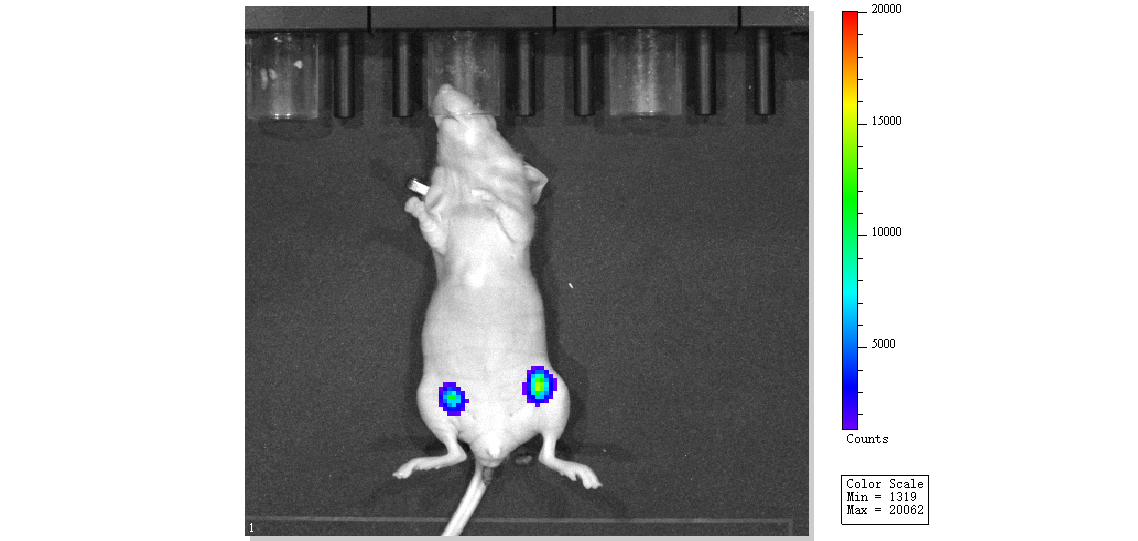

Supplement: Supplementary file 8 — Source data Fig. 6 [file 44321_2024_184_MOESM8_ESM.zip › Fig 6/Fig 6F/17.png]

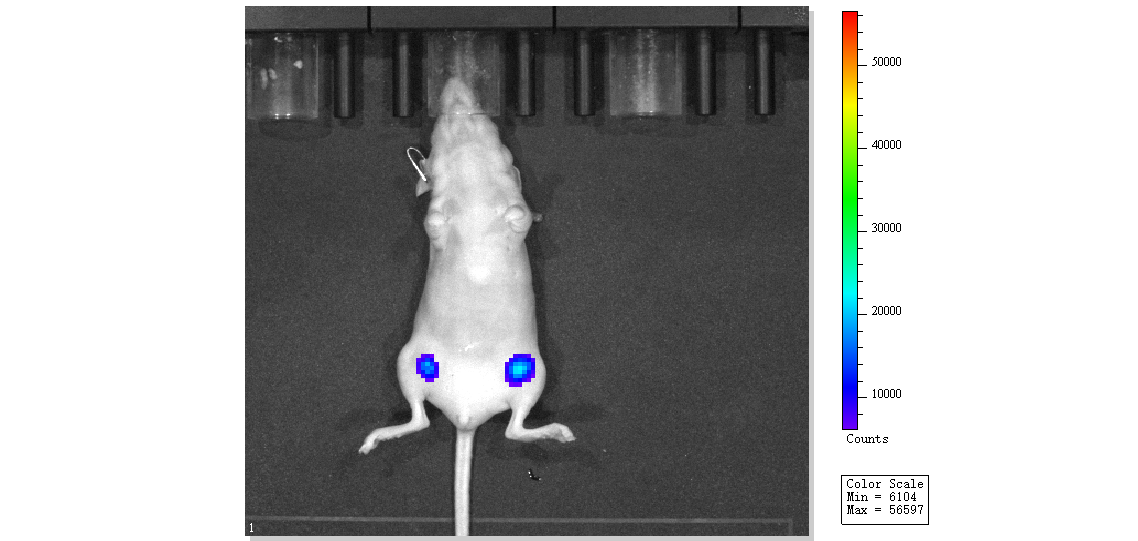

Supplement: Supplementary file 8 — Source data Fig. 6 [file 44321_2024_184_MOESM8_ESM.zip › Fig 6/Fig 6F/16.png]

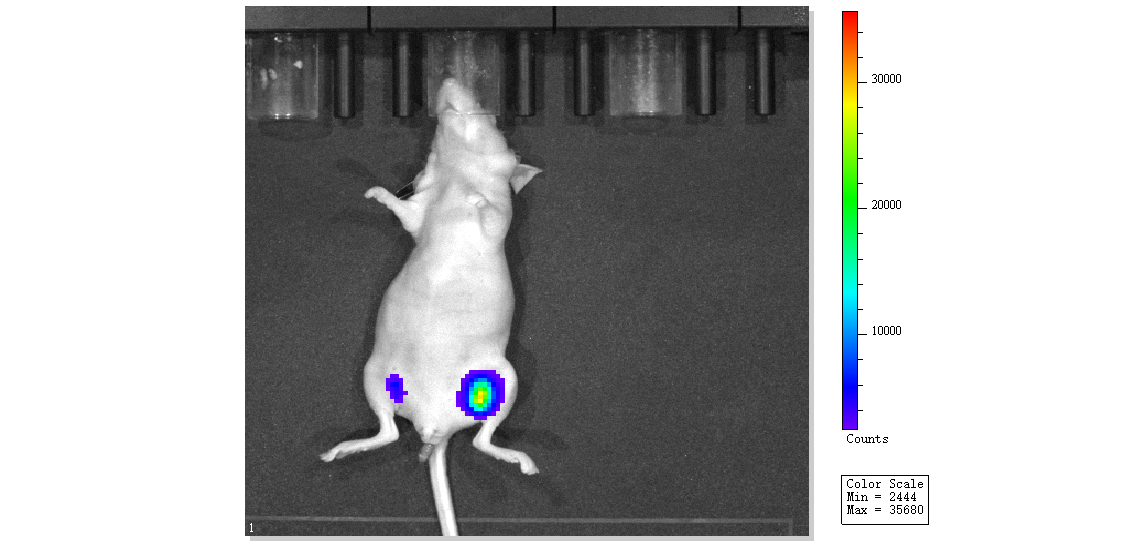

Supplement: Supplementary file 8 — Source data Fig. 6 [file 44321_2024_184_MOESM8_ESM.zip › Fig 6/Fig 6F/12.png]

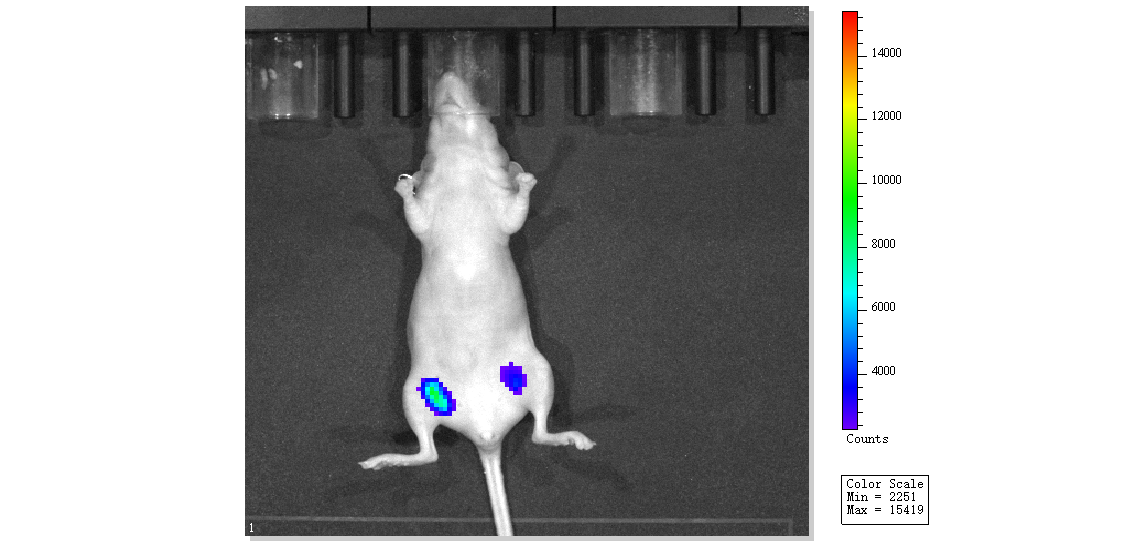

Supplement: Supplementary file 8 — Source data Fig. 6 [file 44321_2024_184_MOESM8_ESM.zip › Fig 6/Fig 6F/13.png]

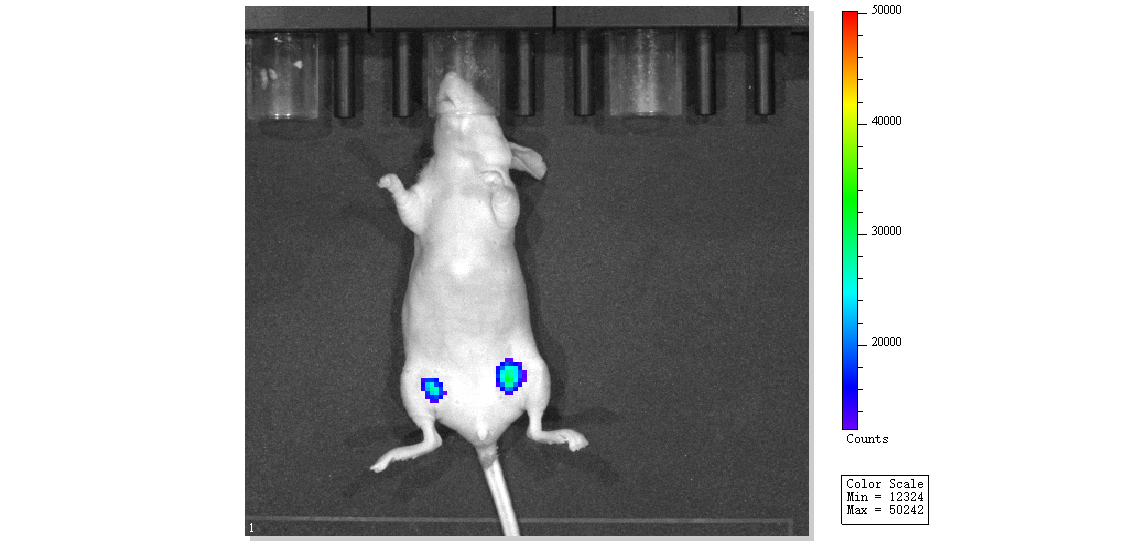

Supplement: Supplementary file 8 — Source data Fig. 6 [file 44321_2024_184_MOESM8_ESM.zip › Fig 6/Fig 6F/11.png]

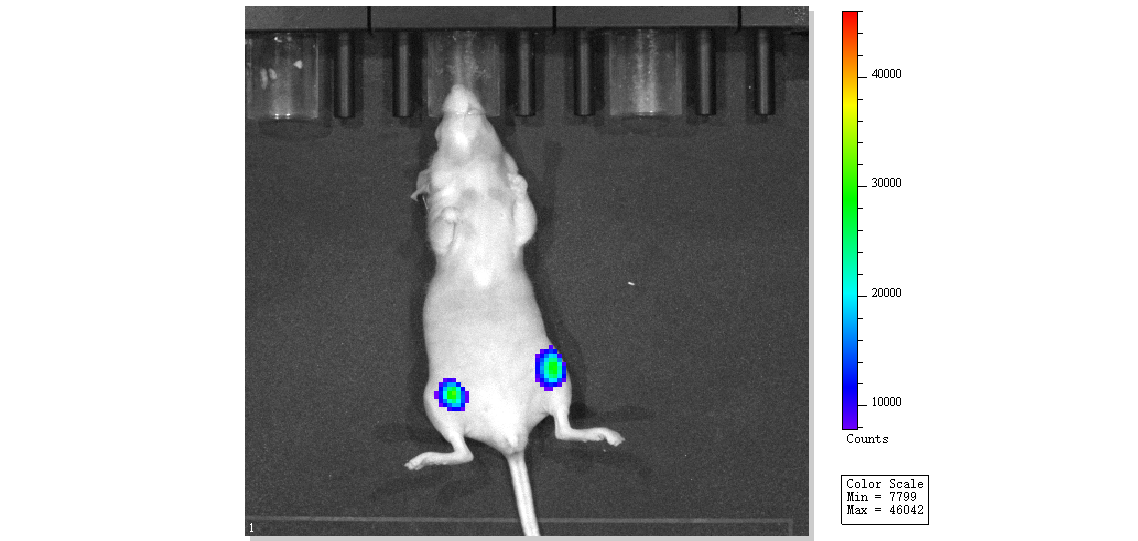

Supplement: Supplementary file 8 — Source data Fig. 6 [file 44321_2024_184_MOESM8_ESM.zip › Fig 6/Fig 6F/18.png]

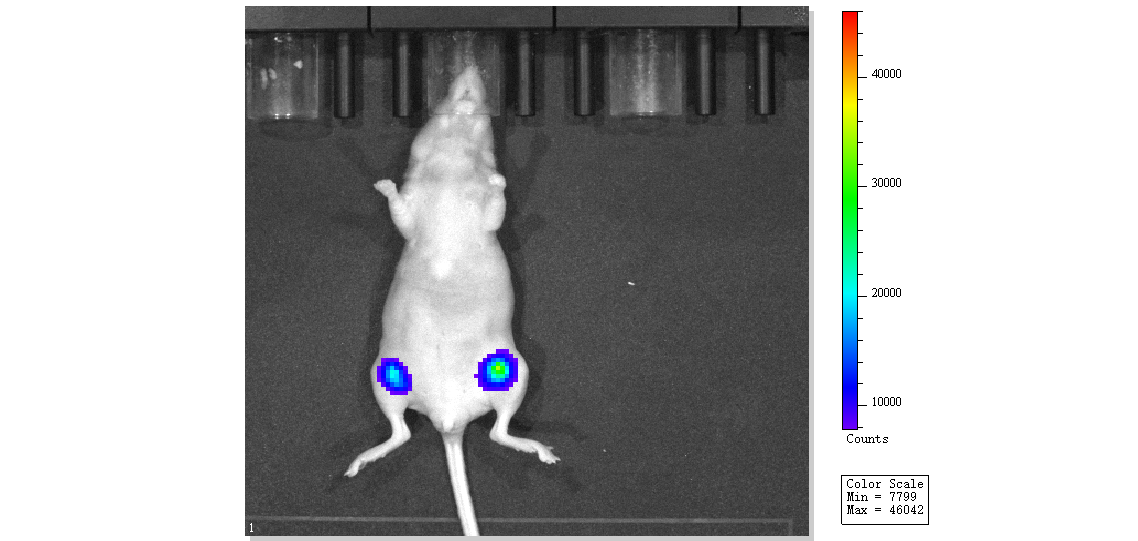

Supplement: Supplementary file 8 — Source data Fig. 6 [file 44321_2024_184_MOESM8_ESM.zip › Fig 6/Fig 6F/19.png]

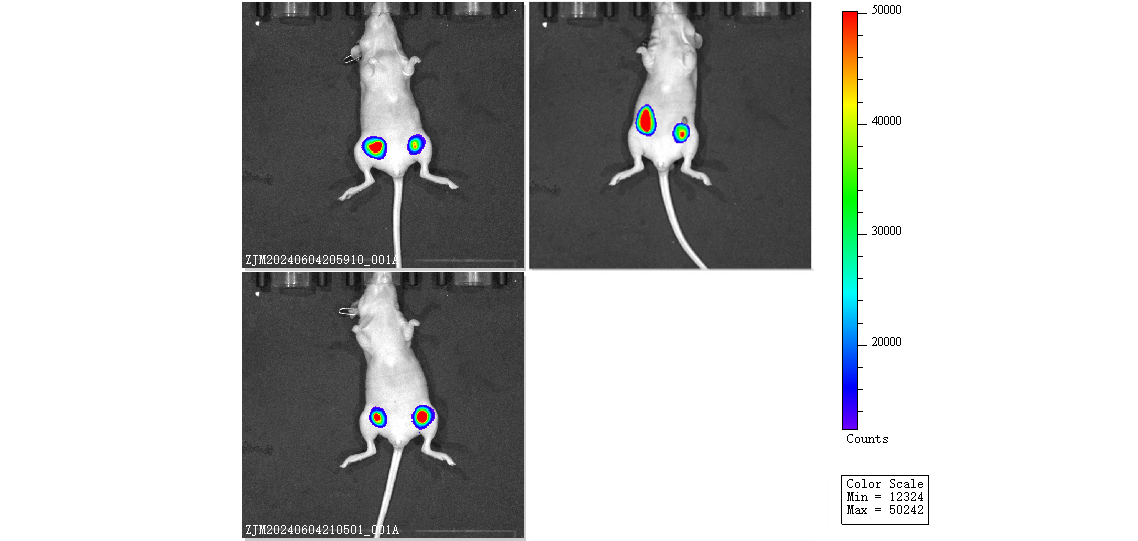

Supplement: Supplementary file 8 — Source data Fig. 6 [file 44321_2024_184_MOESM8_ESM.zip › Fig 6/Fig 6F/2.png]

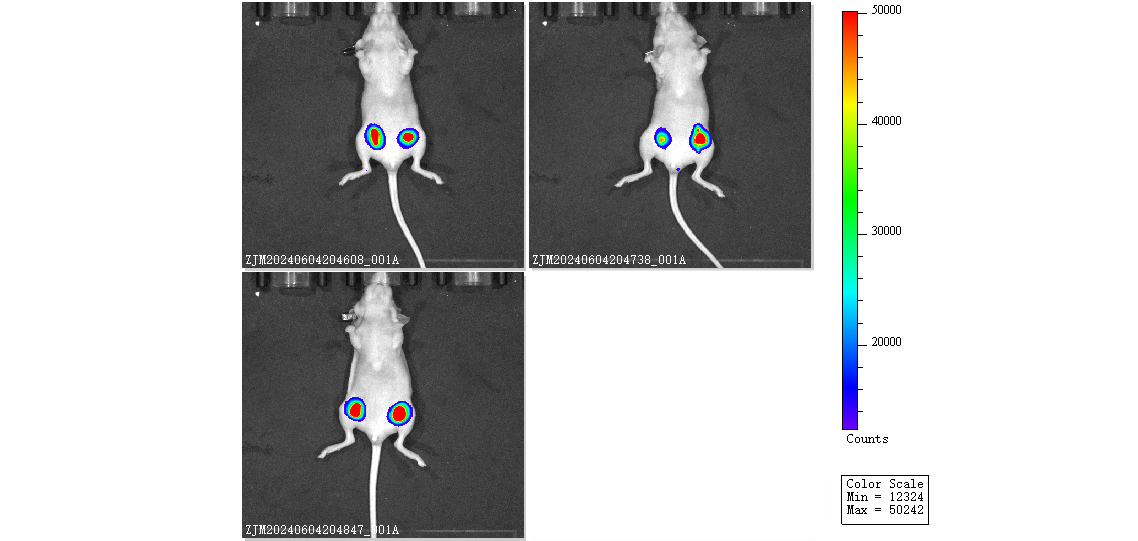

Supplement: Supplementary file 8 — Source data Fig. 6 [file 44321_2024_184_MOESM8_ESM.zip › Fig 6/Fig 6F/3.png]

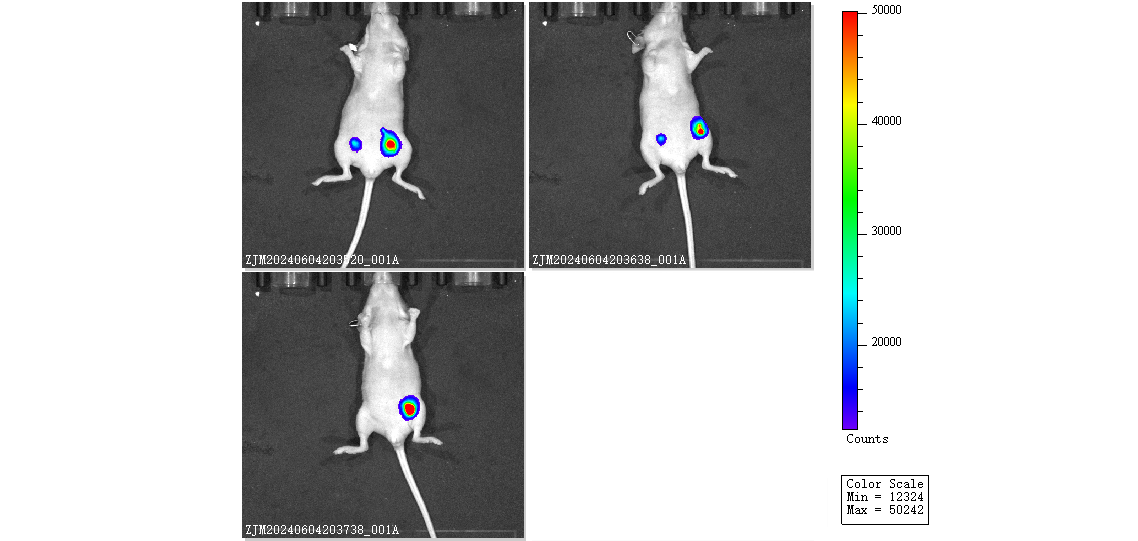

Supplement: Supplementary file 8 — Source data Fig. 6 [file 44321_2024_184_MOESM8_ESM.zip › Fig 6/Fig 6F/1.png]

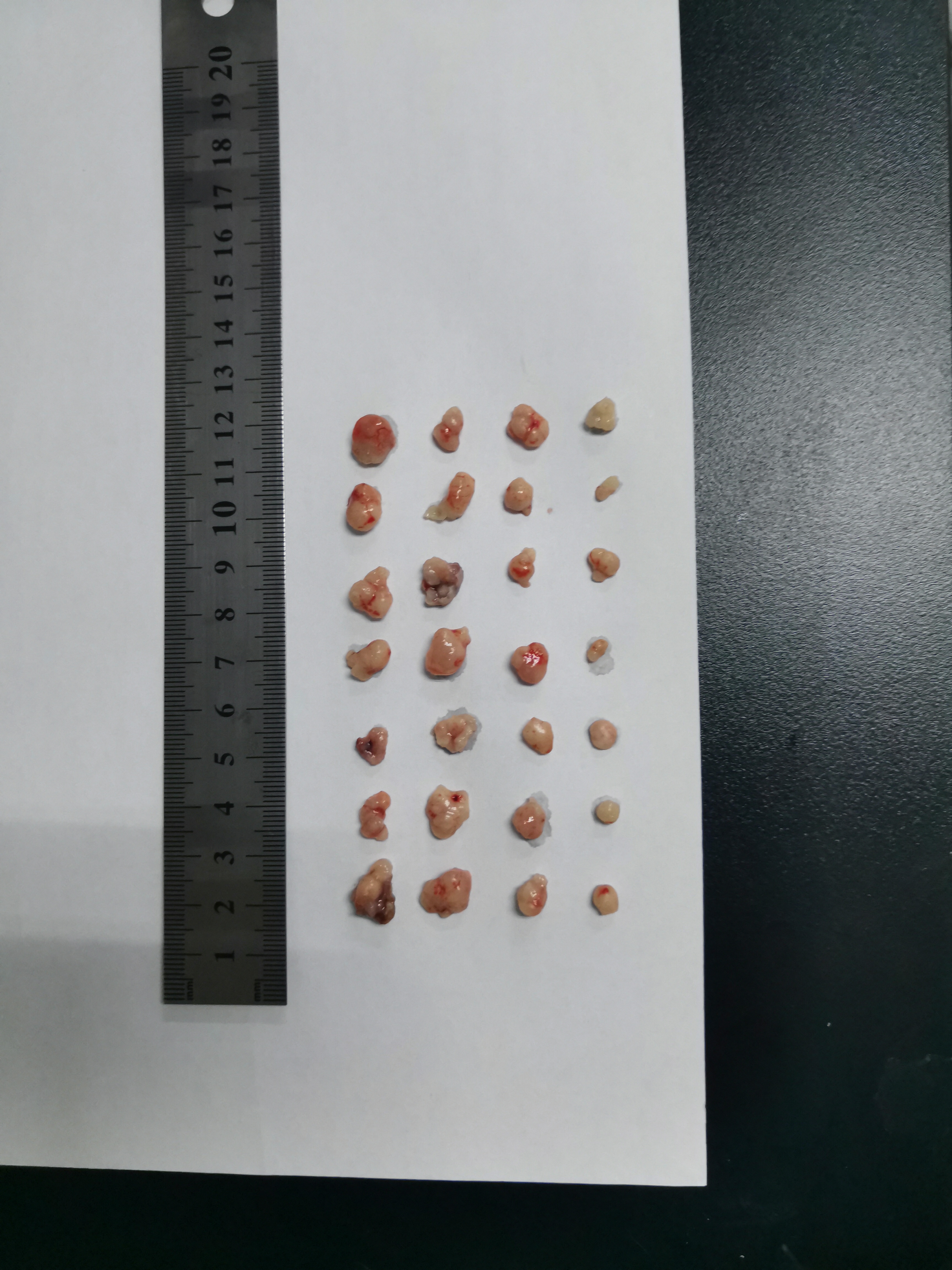

Supplement: Supplementary file 8 — Source data Fig. 6 [file 44321_2024_184_MOESM8_ESM.zip › Fig 6/Fig 6B/078e962646e0e4c47e1980e8f4ee177.jpg]

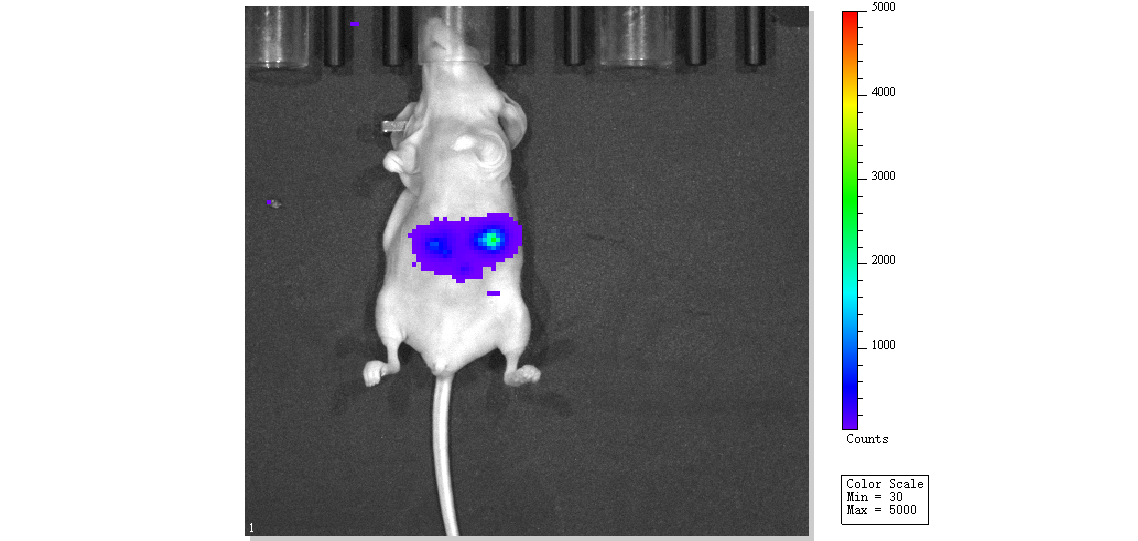

Supplement: Supplementary file 8 — Source data Fig. 6 [file 44321_2024_184_MOESM8_ESM.zip › Fig 6/Fig 6G/image/1/8.png]

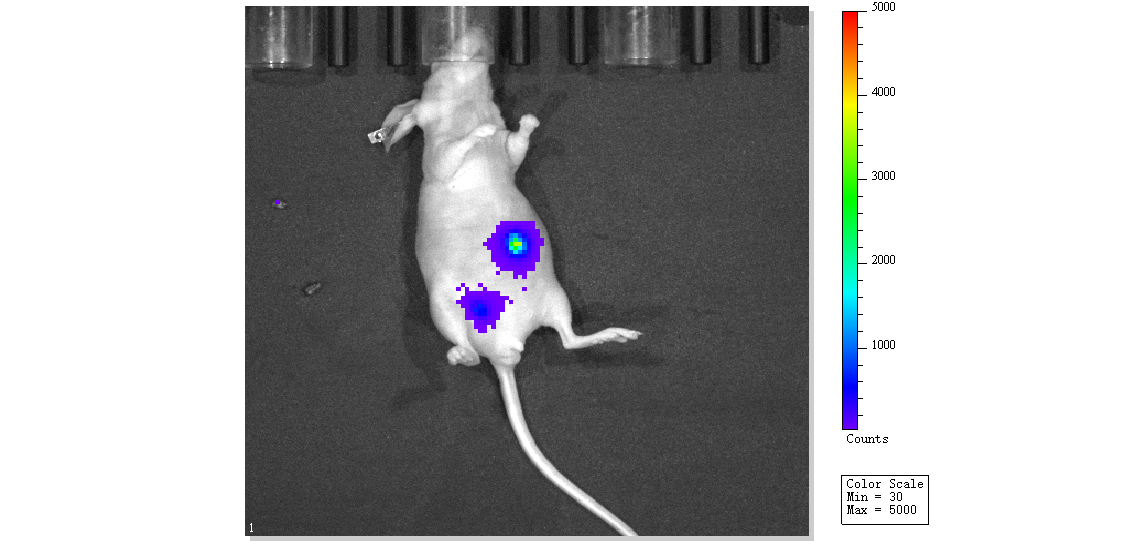

Supplement: Supplementary file 8 — Source data Fig. 6 [file 44321_2024_184_MOESM8_ESM.zip › Fig 6/Fig 6G/image/1/9.png]

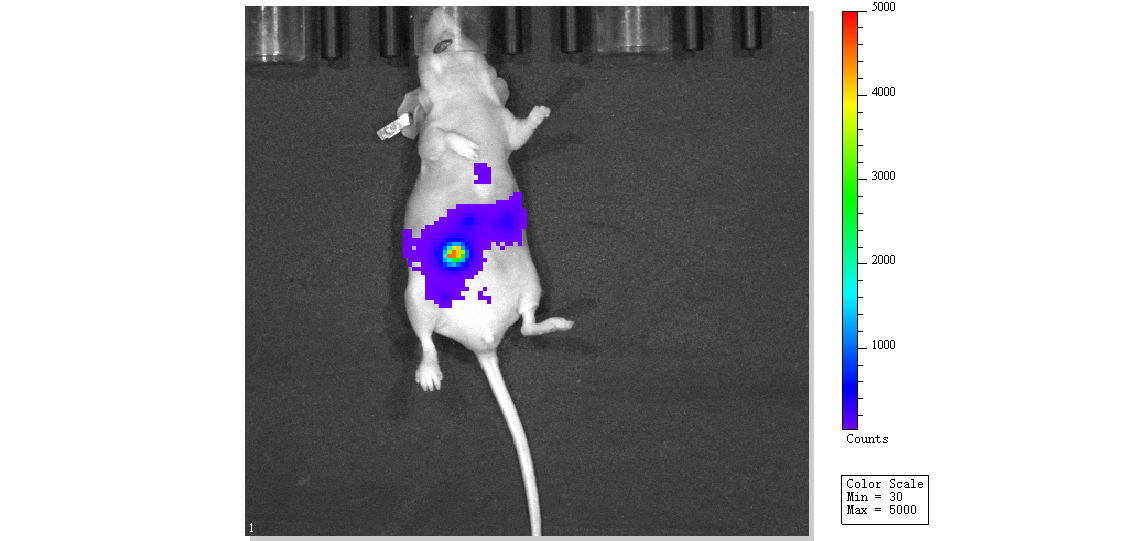

Supplement: Supplementary file 8 — Source data Fig. 6 [file 44321_2024_184_MOESM8_ESM.zip › Fig 6/Fig 6G/image/1/12.png]

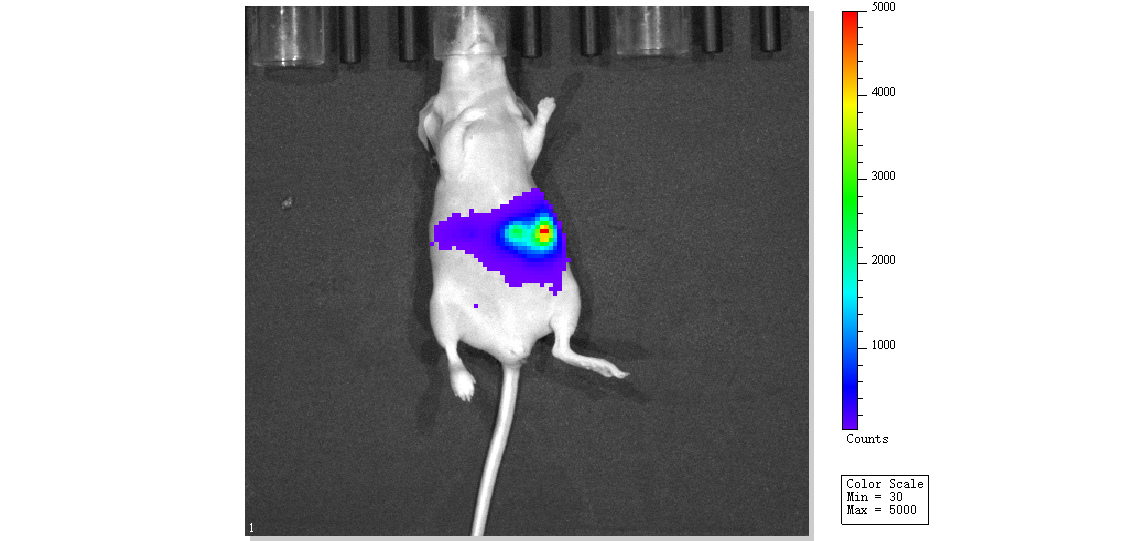

Supplement: Supplementary file 8 — Source data Fig. 6 [file 44321_2024_184_MOESM8_ESM.zip › Fig 6/Fig 6G/image/1/11.png]

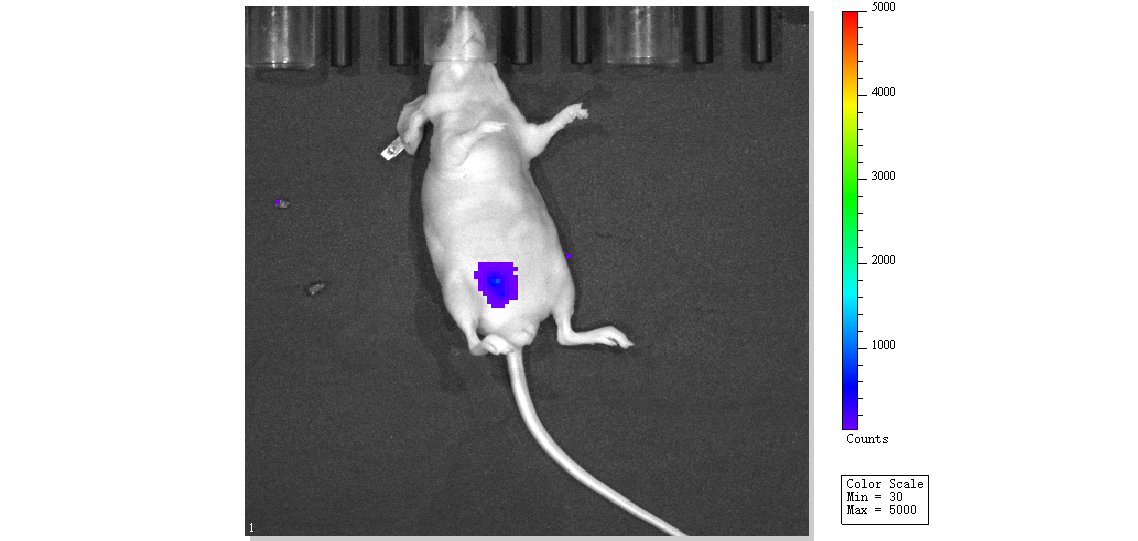

Supplement: Supplementary file 8 — Source data Fig. 6 [file 44321_2024_184_MOESM8_ESM.zip › Fig 6/Fig 6G/image/1/10.png]

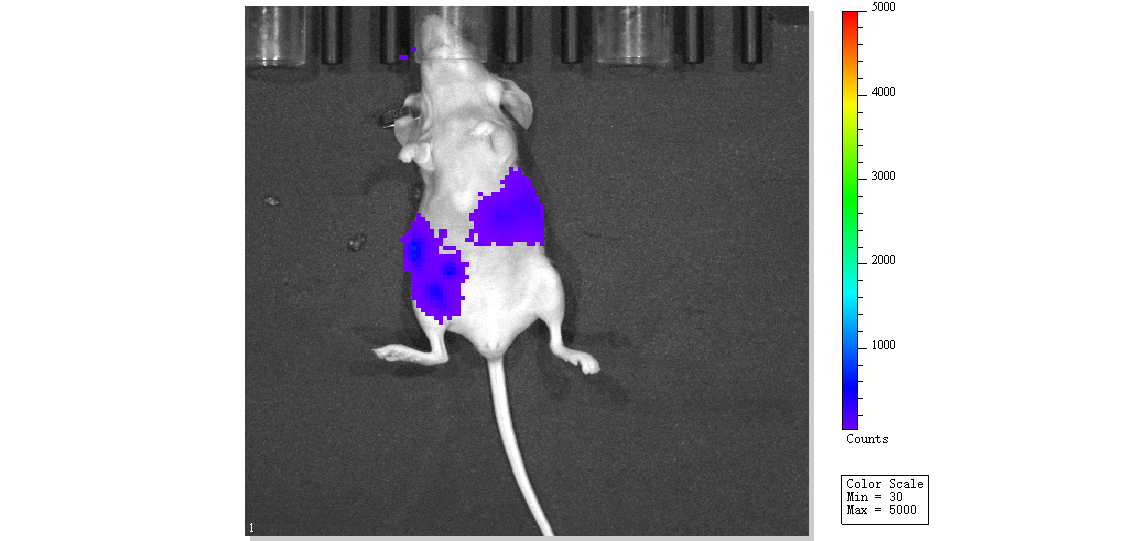

Supplement: Supplementary file 8 — Source data Fig. 6 [file 44321_2024_184_MOESM8_ESM.zip › Fig 6/Fig 6G/image/1/4.png]

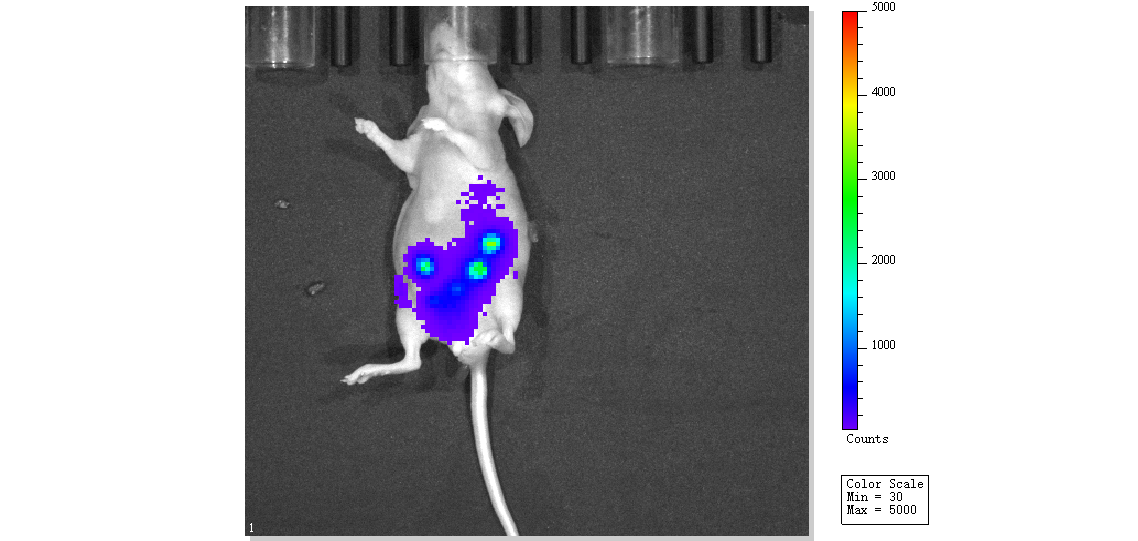

Supplement: Supplementary file 8 — Source data Fig. 6 [file 44321_2024_184_MOESM8_ESM.zip › Fig 6/Fig 6G/image/1/5.png]

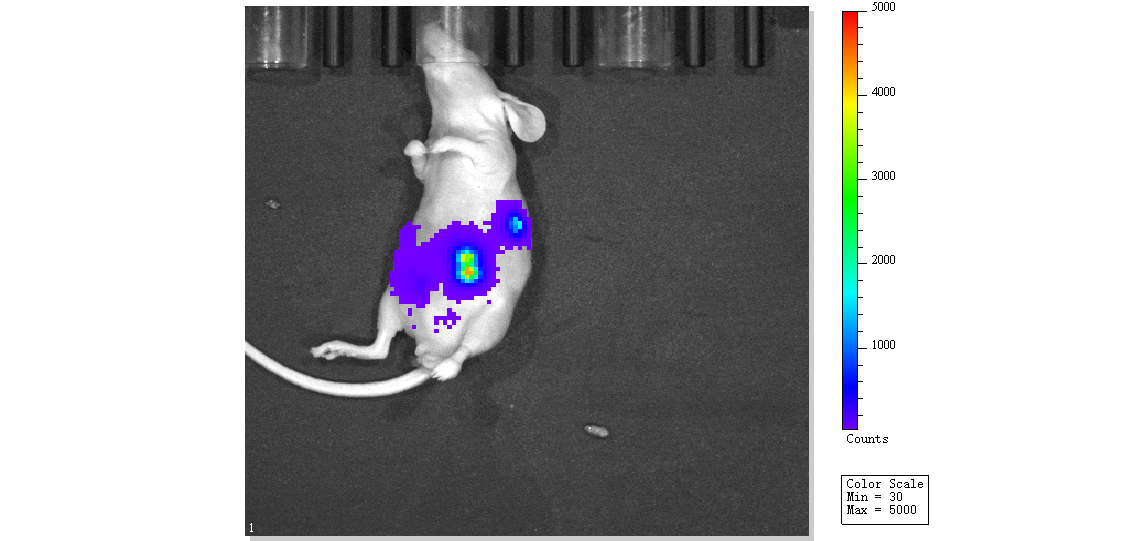

Supplement: Supplementary file 8 — Source data Fig. 6 [file 44321_2024_184_MOESM8_ESM.zip › Fig 6/Fig 6G/image/1/7.png]

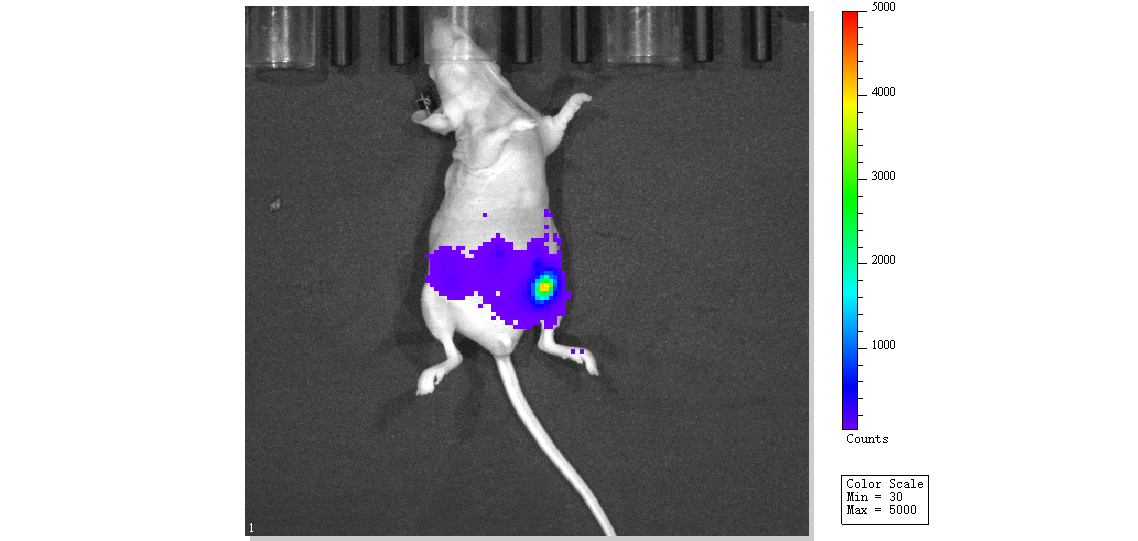

Supplement: Supplementary file 8 — Source data Fig. 6 [file 44321_2024_184_MOESM8_ESM.zip › Fig 6/Fig 6G/image/1/6.png]

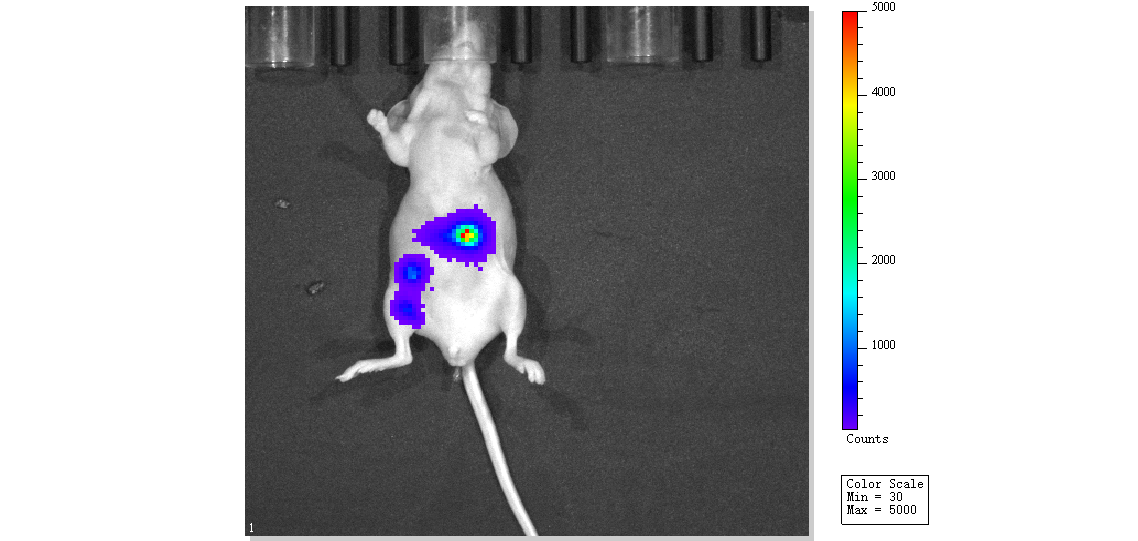

Supplement: Supplementary file 8 — Source data Fig. 6 [file 44321_2024_184_MOESM8_ESM.zip › Fig 6/Fig 6G/image/1/2.png]

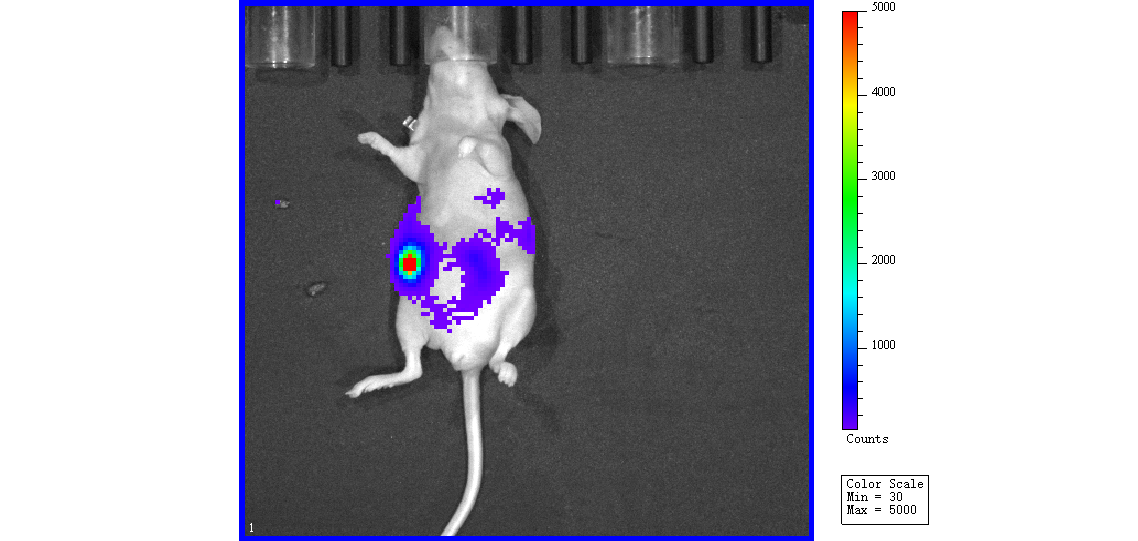

Supplement: Supplementary file 8 — Source data Fig. 6 [file 44321_2024_184_MOESM8_ESM.zip › Fig 6/Fig 6G/image/1/3.png]

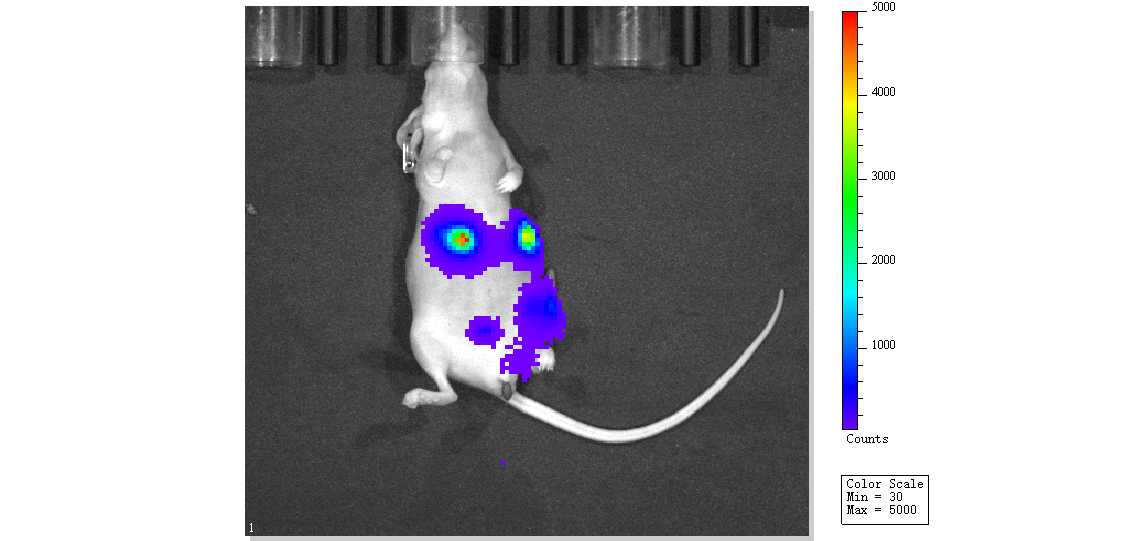

Supplement: Supplementary file 8 — Source data Fig. 6 [file 44321_2024_184_MOESM8_ESM.zip › Fig 6/Fig 6G/image/1/1.png]

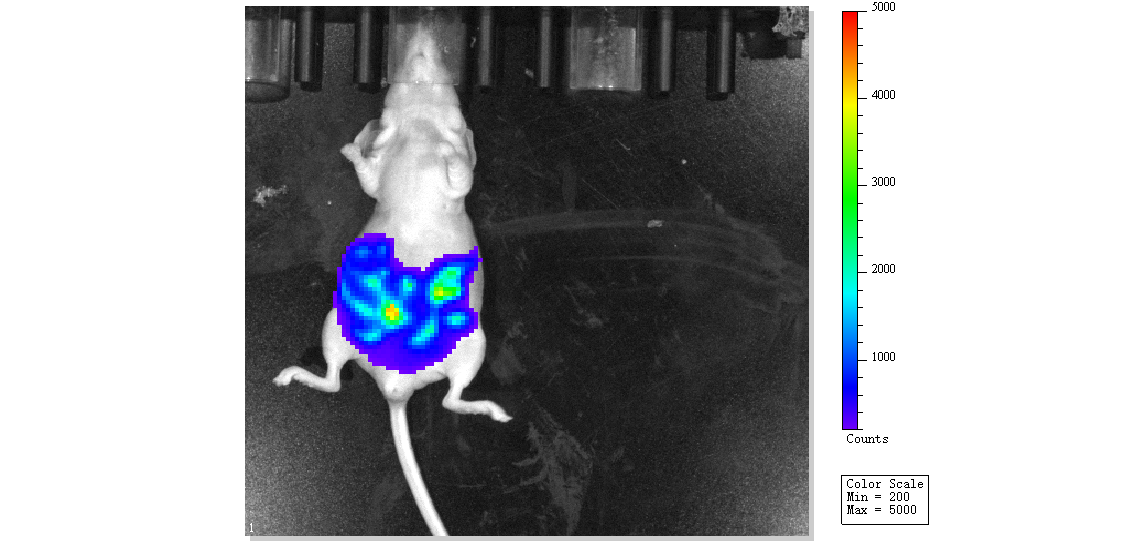

Supplement: Supplementary file 8 — Source data Fig. 6 [file 44321_2024_184_MOESM8_ESM.zip › Fig 6/Fig 6G/image/3/8.png]

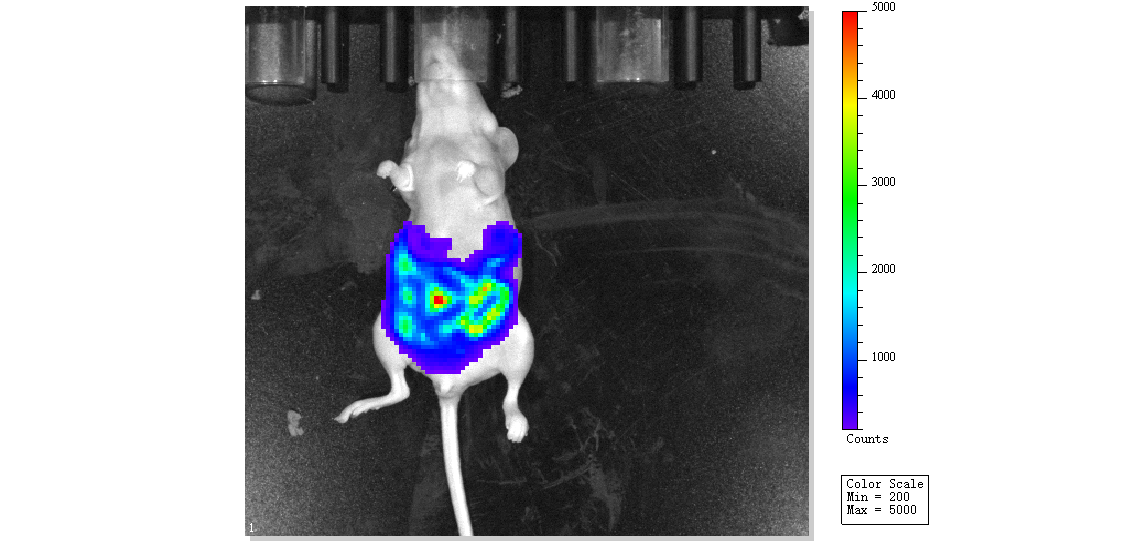

Supplement: Supplementary file 8 — Source data Fig. 6 [file 44321_2024_184_MOESM8_ESM.zip › Fig 6/Fig 6G/image/3/9.png]

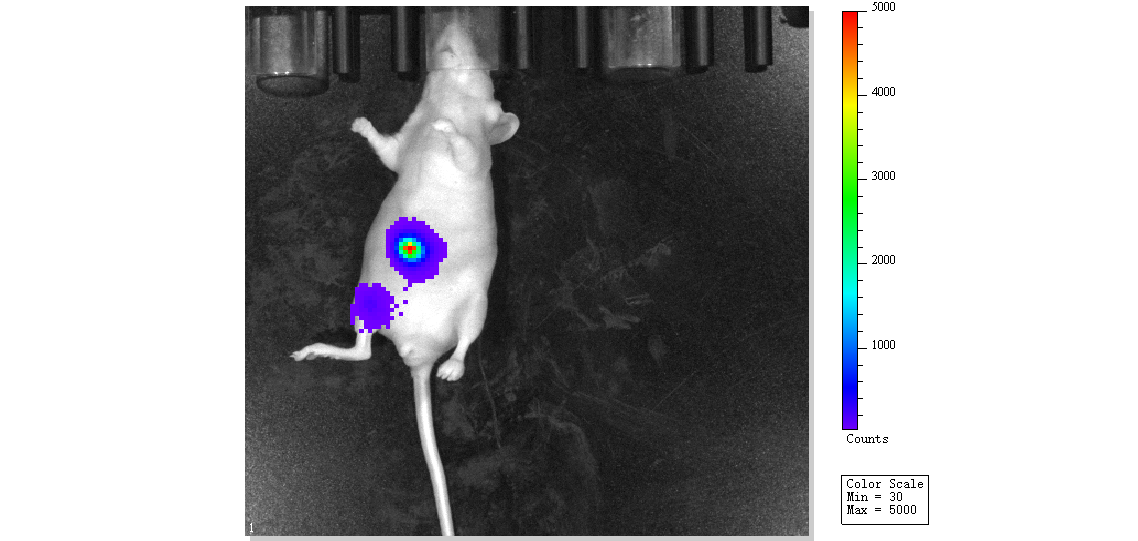

Supplement: Supplementary file 8 — Source data Fig. 6 [file 44321_2024_184_MOESM8_ESM.zip › Fig 6/Fig 6G/image/3/12.png]

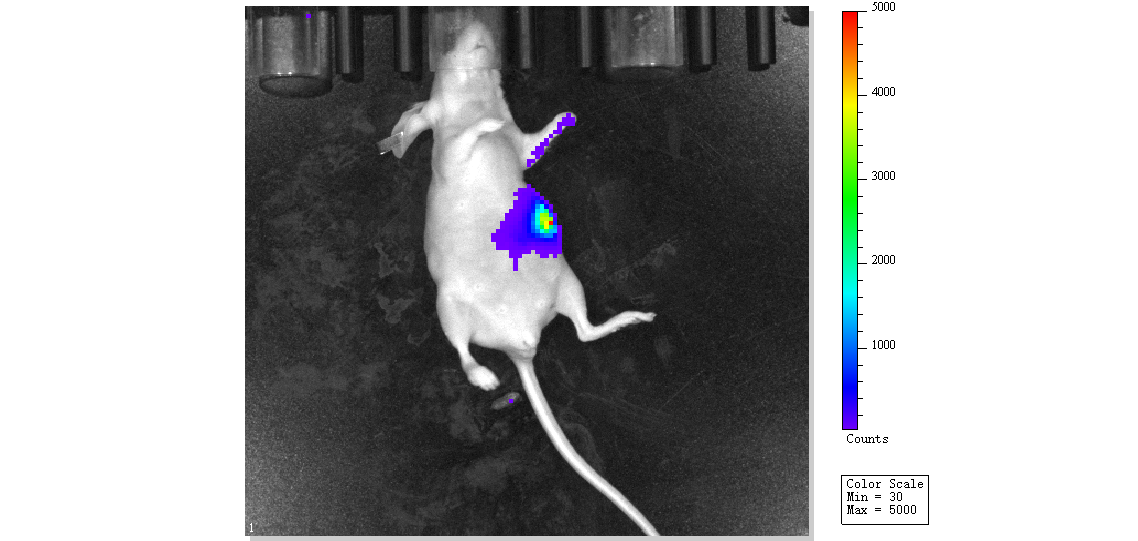

Supplement: Supplementary file 8 — Source data Fig. 6 [file 44321_2024_184_MOESM8_ESM.zip › Fig 6/Fig 6G/image/3/11.png]

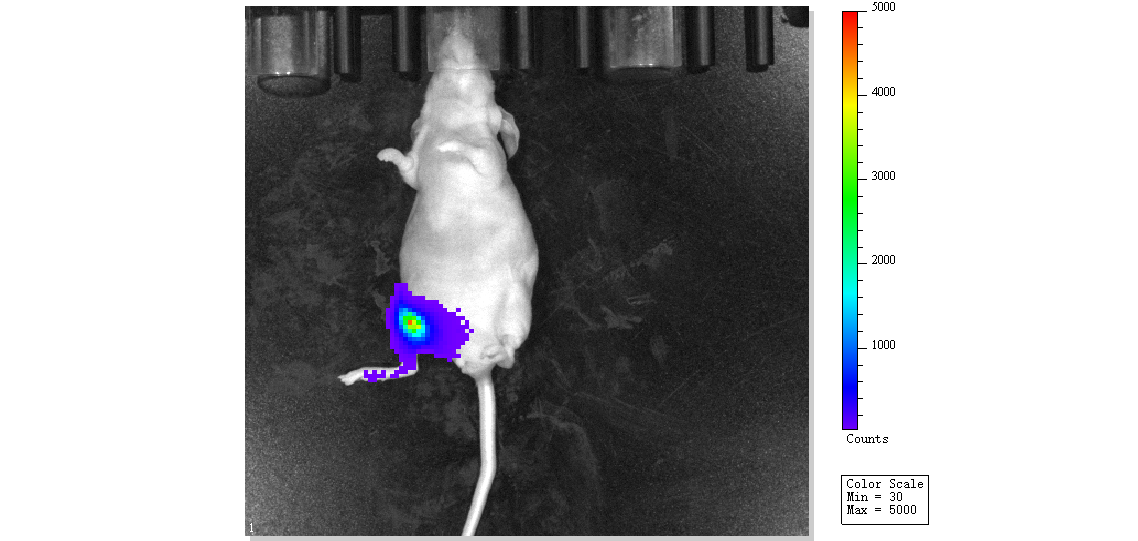

Supplement: Supplementary file 8 — Source data Fig. 6 [file 44321_2024_184_MOESM8_ESM.zip › Fig 6/Fig 6G/image/3/10.png]

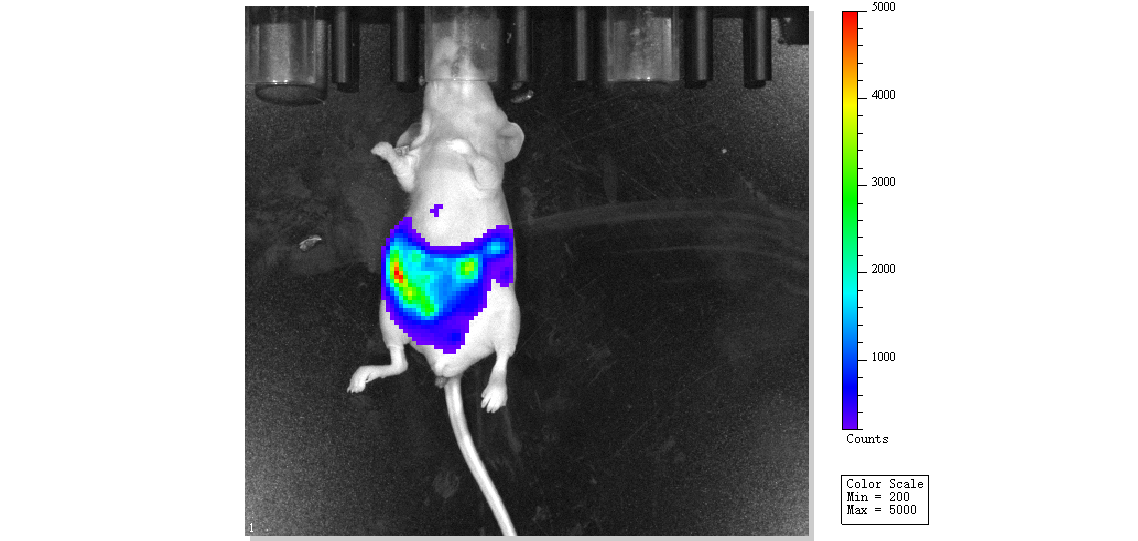

Supplement: Supplementary file 8 — Source data Fig. 6 [file 44321_2024_184_MOESM8_ESM.zip › Fig 6/Fig 6G/image/3/4.png]

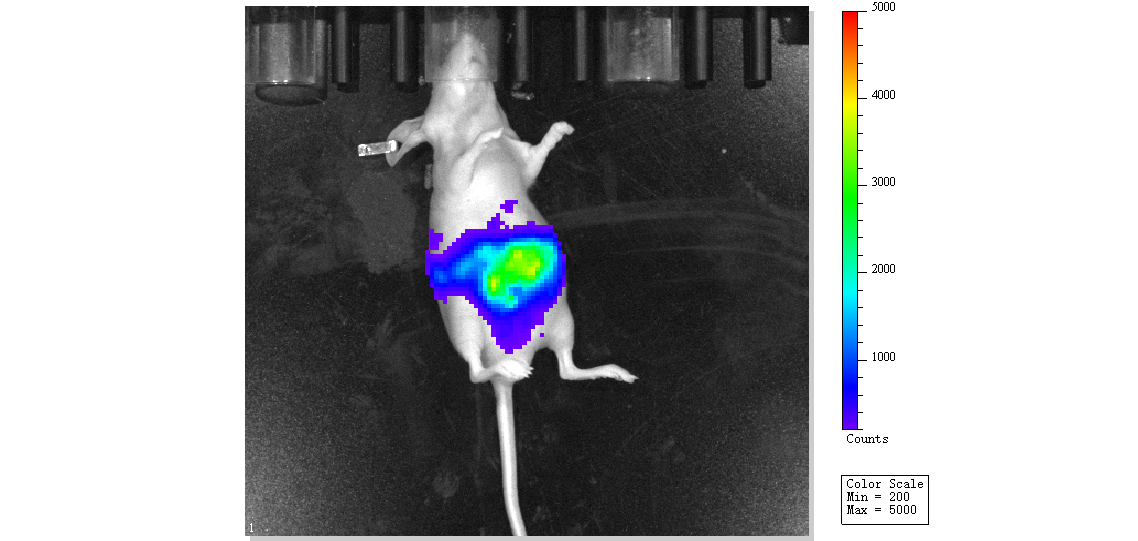

Supplement: Supplementary file 8 — Source data Fig. 6 [file 44321_2024_184_MOESM8_ESM.zip › Fig 6/Fig 6G/image/3/5.png]

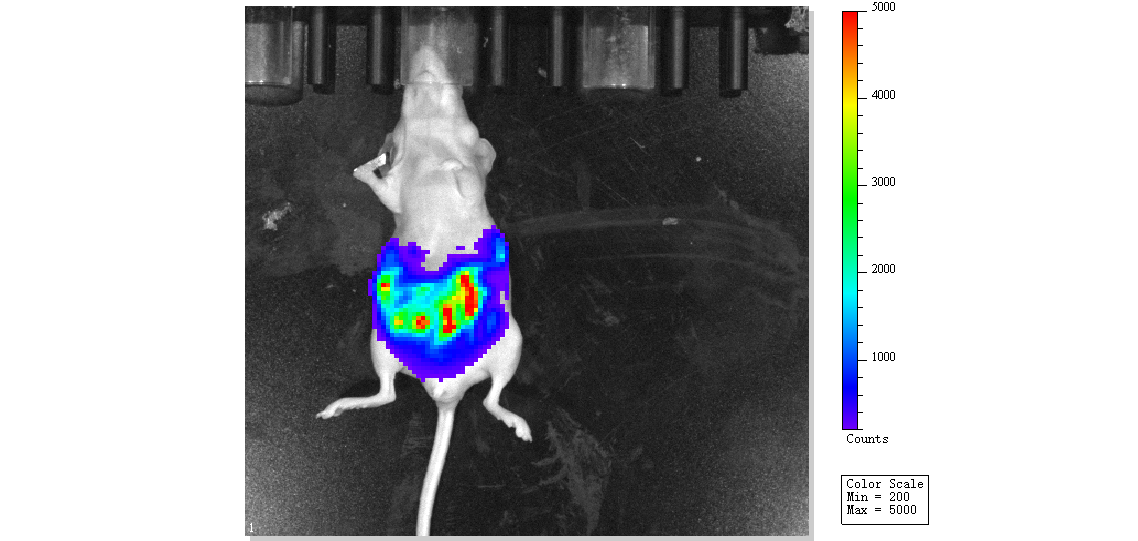

Supplement: Supplementary file 8 — Source data Fig. 6 [file 44321_2024_184_MOESM8_ESM.zip › Fig 6/Fig 6G/image/3/7.png]

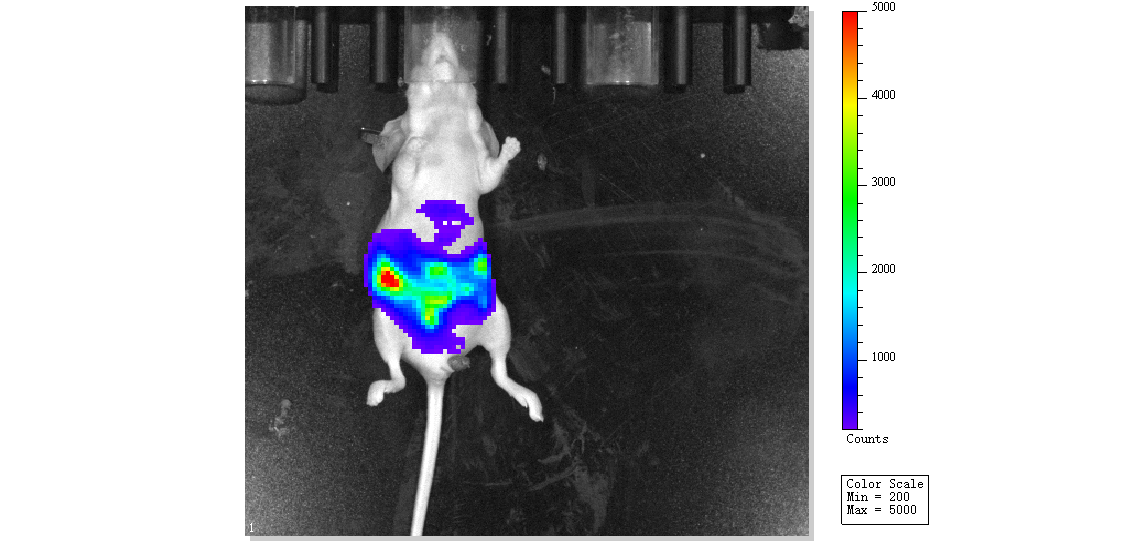

Supplement: Supplementary file 8 — Source data Fig. 6 [file 44321_2024_184_MOESM8_ESM.zip › Fig 6/Fig 6G/image/3/6.png]
