# Supplementary figures and images for: A therapeutic regimen using neoantigen-specific TCR-T cells for HLA-A*2402-positive solid tumors (part 2 of 2)
Source: EMBO Mol Med. 2025 Jan 2;17(2):365–83. doi: 10.1038/s44321-024-00184-1 (PMC11821884; doi:10.1038/s44321-024-00184-1)

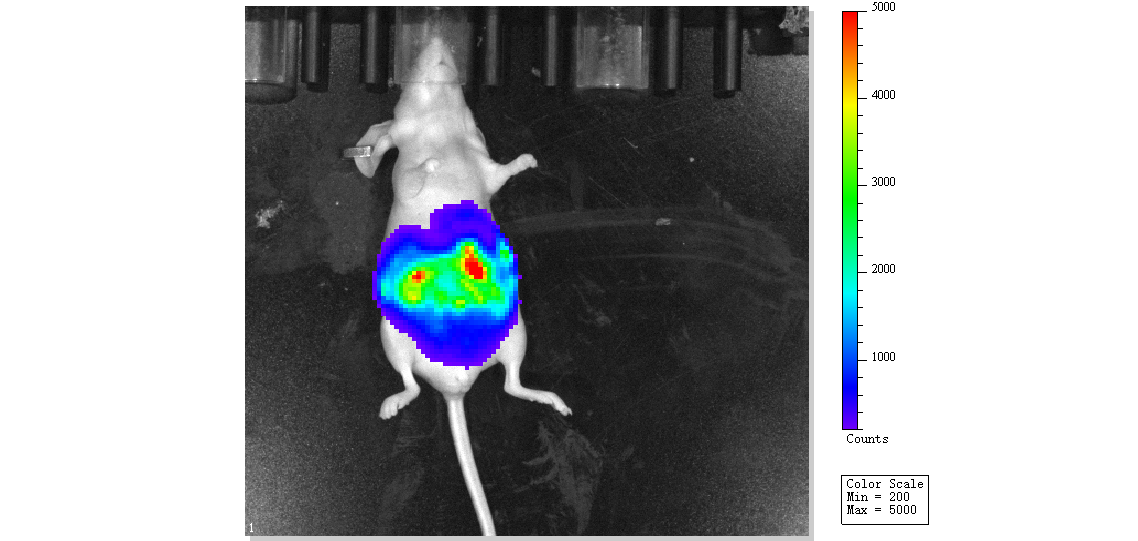

Supplement: Supplementary file 8 — Source data Fig. 6 [file 44321_2024_184_MOESM8_ESM.zip › Fig 6/Fig 6G/image/3/2.png]

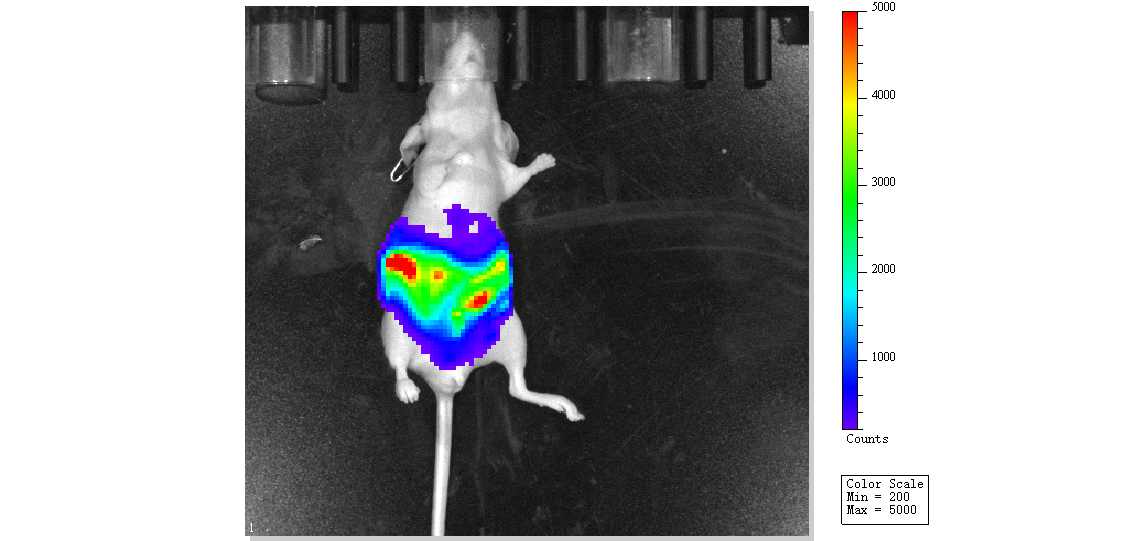

Supplement: Supplementary file 8 — Source data Fig. 6 [file 44321_2024_184_MOESM8_ESM.zip › Fig 6/Fig 6G/image/3/3.png]

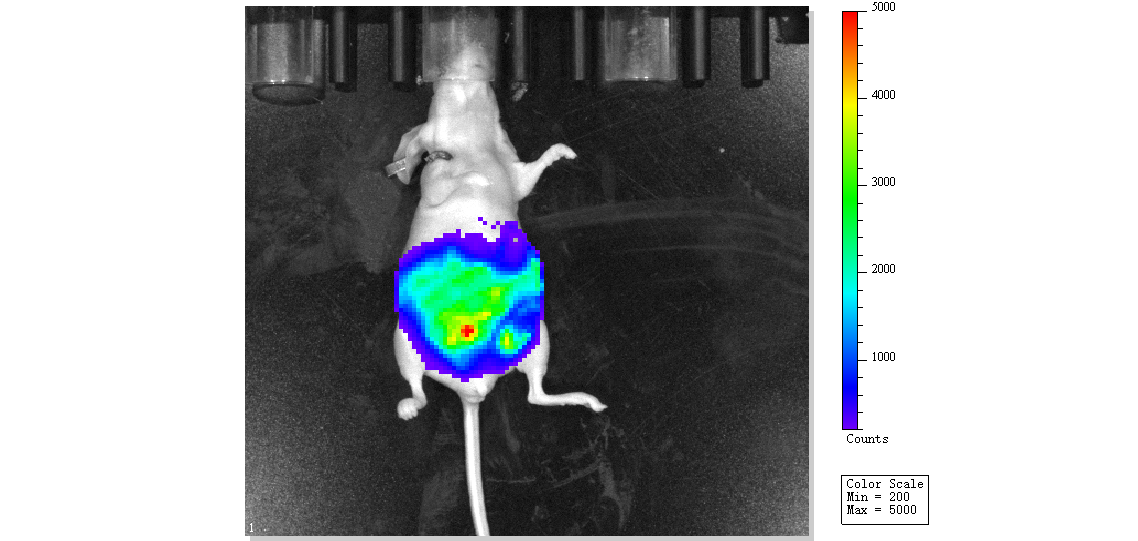

Supplement: Supplementary file 8 — Source data Fig. 6 [file 44321_2024_184_MOESM8_ESM.zip › Fig 6/Fig 6G/image/3/1.png]

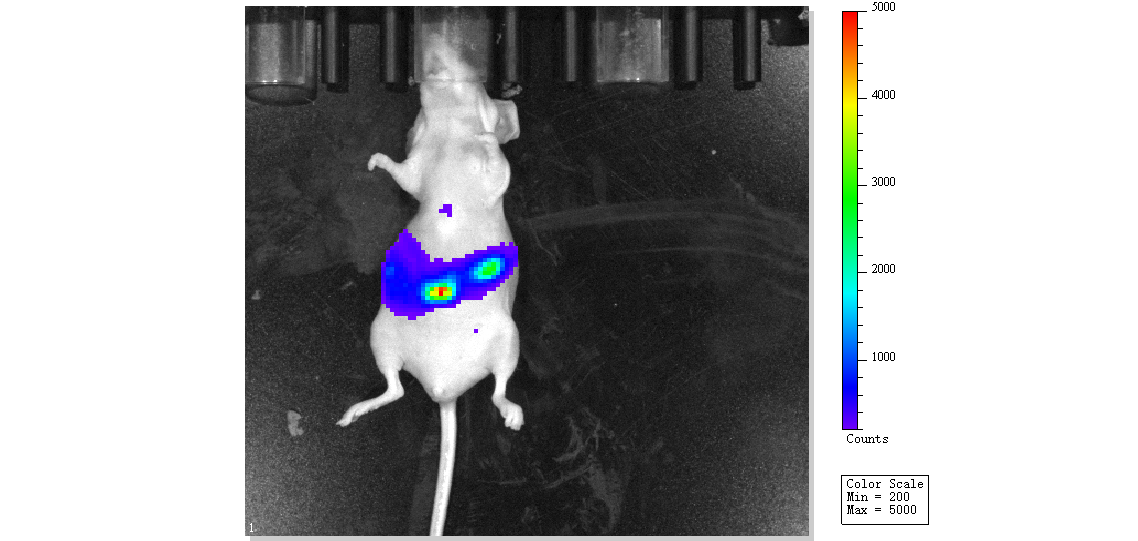

Supplement: Supplementary file 8 — Source data Fig. 6 [file 44321_2024_184_MOESM8_ESM.zip › Fig 6/Fig 6G/image/2/8.png]

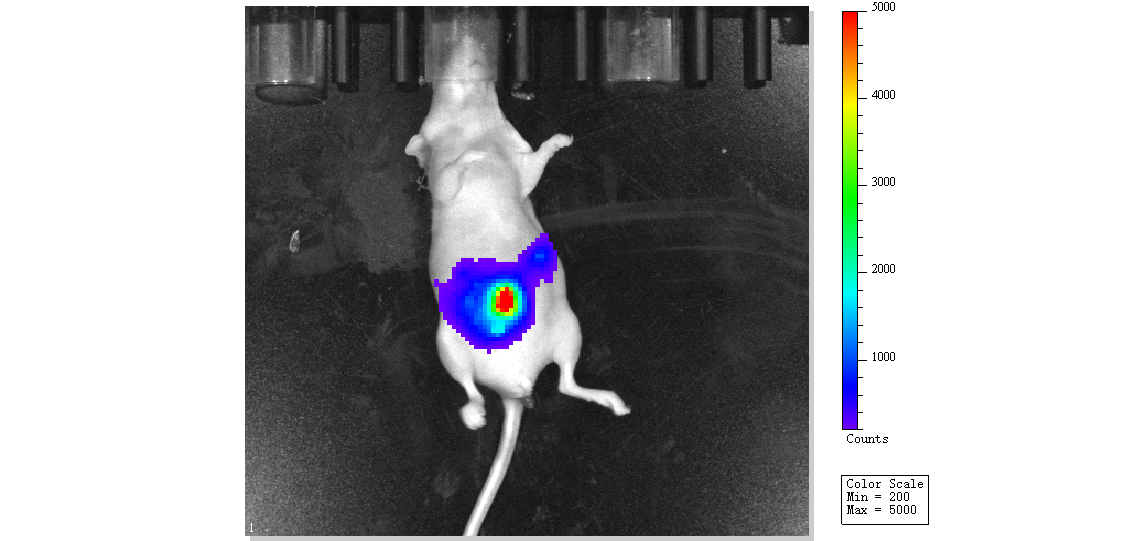

Supplement: Supplementary file 8 — Source data Fig. 6 [file 44321_2024_184_MOESM8_ESM.zip › Fig 6/Fig 6G/image/2/9.png]

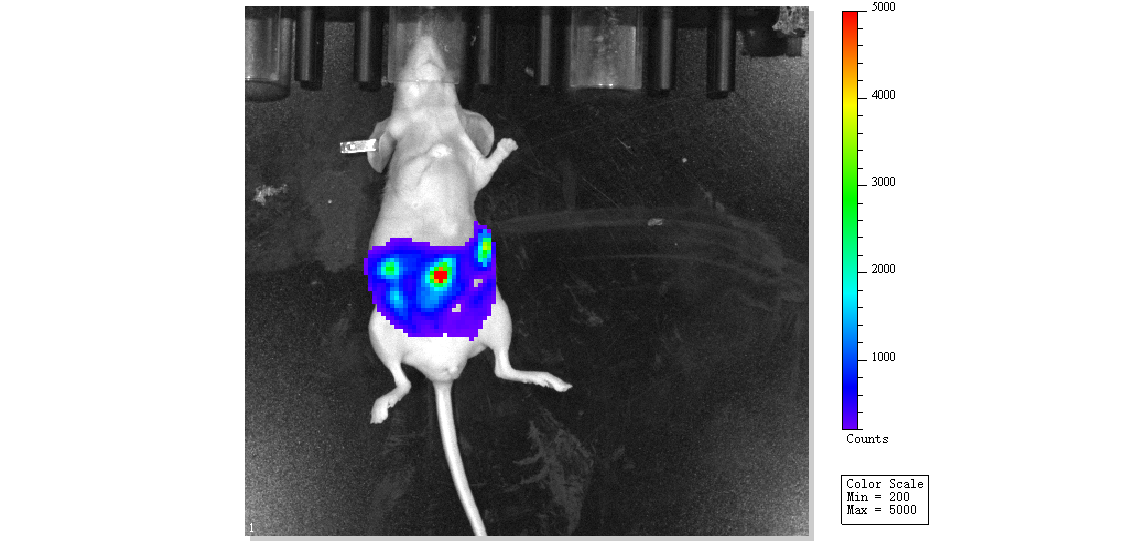

Supplement: Supplementary file 8 — Source data Fig. 6 [file 44321_2024_184_MOESM8_ESM.zip › Fig 6/Fig 6G/image/2/12.png]

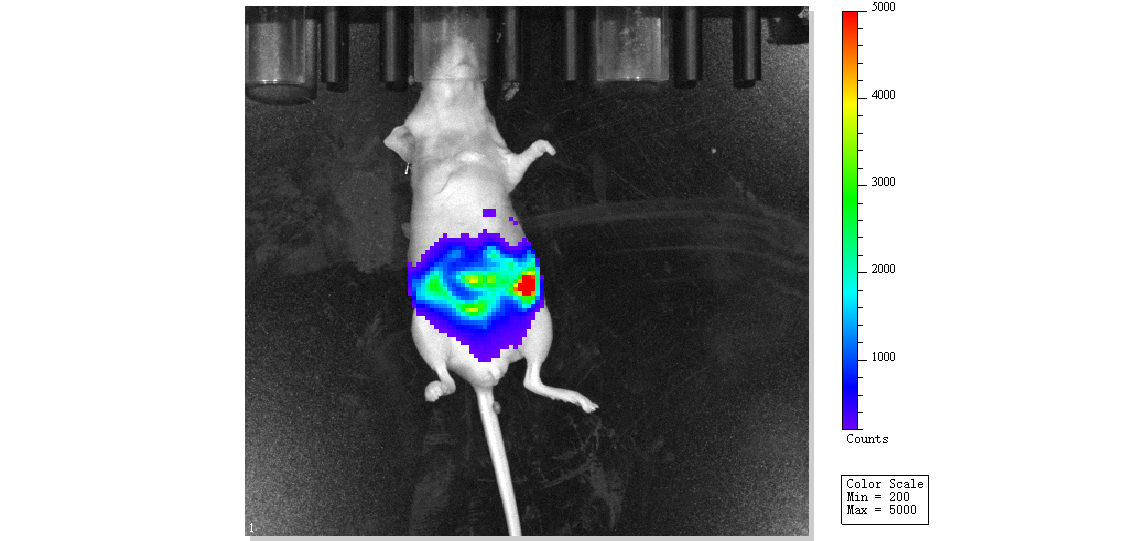

Supplement: Supplementary file 8 — Source data Fig. 6 [file 44321_2024_184_MOESM8_ESM.zip › Fig 6/Fig 6G/image/2/11.png]

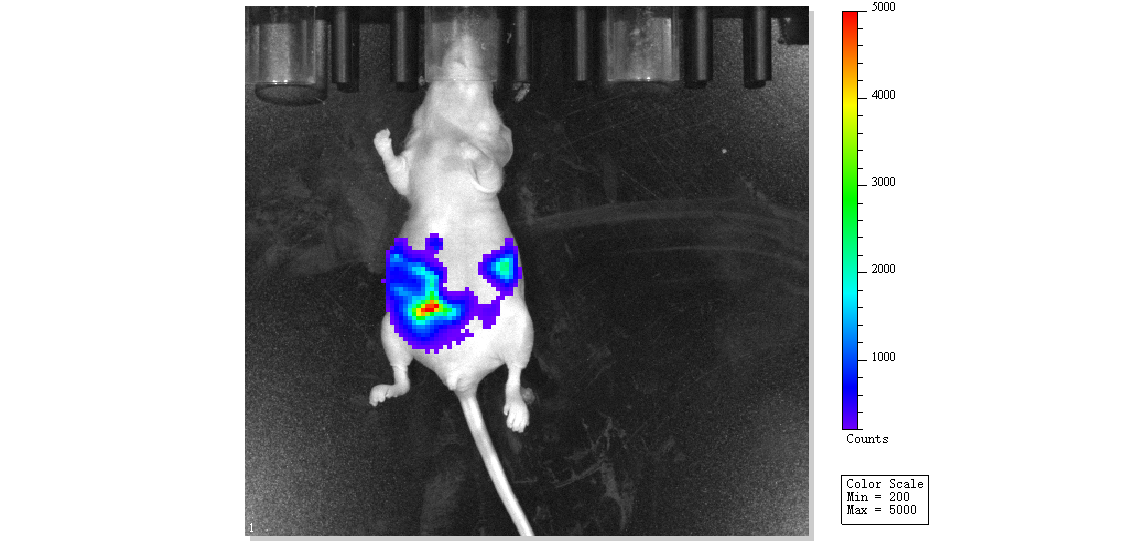

Supplement: Supplementary file 8 — Source data Fig. 6 [file 44321_2024_184_MOESM8_ESM.zip › Fig 6/Fig 6G/image/2/10.png]

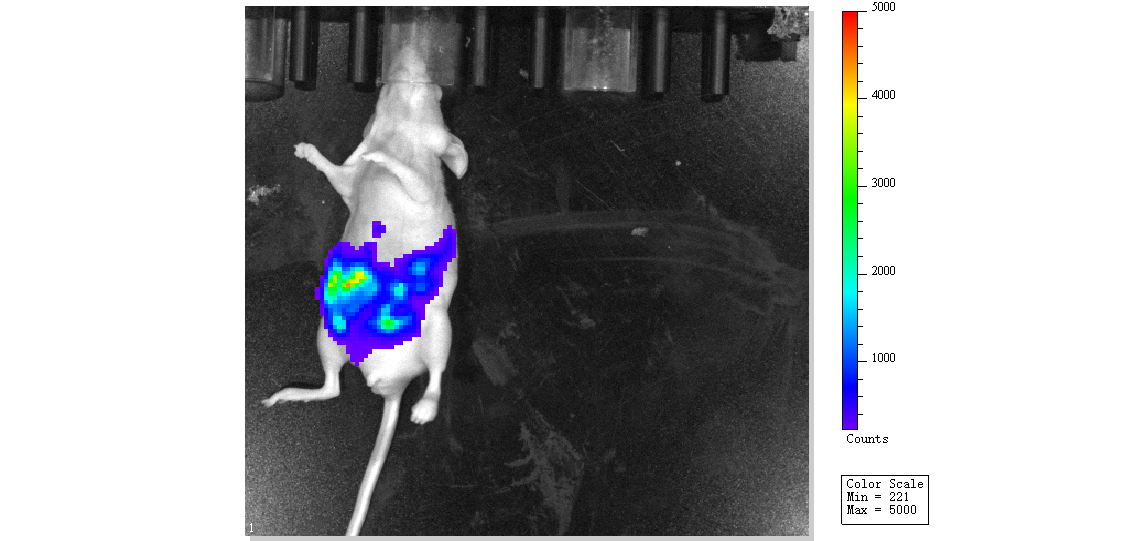

Supplement: Supplementary file 8 — Source data Fig. 6 [file 44321_2024_184_MOESM8_ESM.zip › Fig 6/Fig 6G/image/2/4.png]

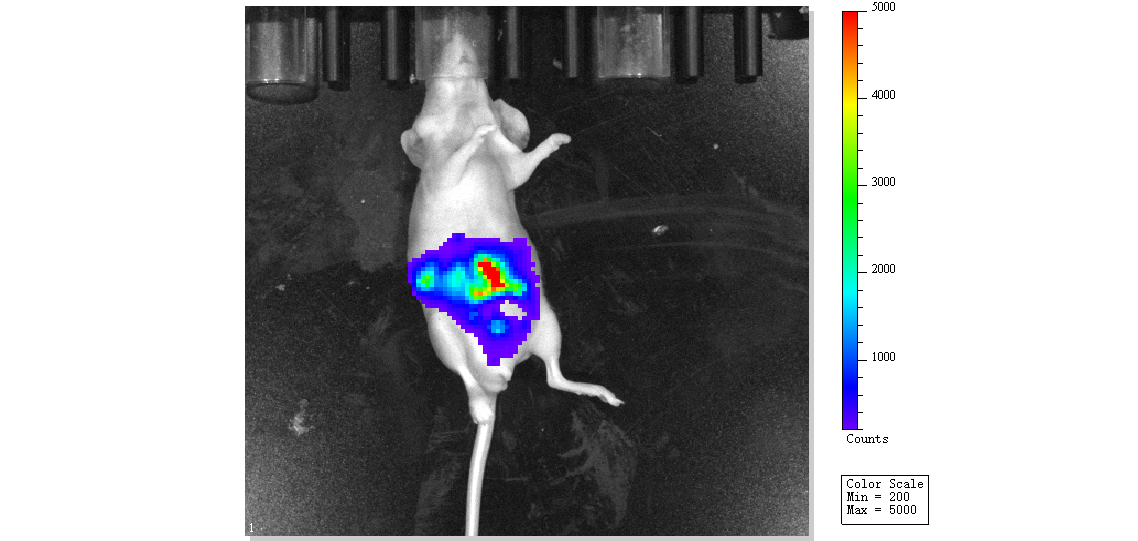

Supplement: Supplementary file 8 — Source data Fig. 6 [file 44321_2024_184_MOESM8_ESM.zip › Fig 6/Fig 6G/image/2/5.png]

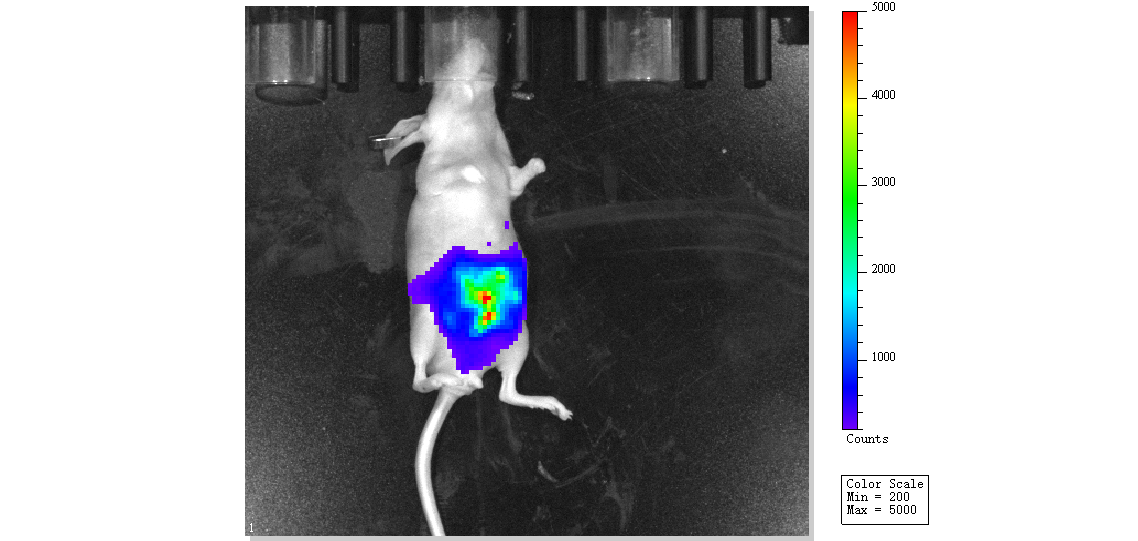

Supplement: Supplementary file 8 — Source data Fig. 6 [file 44321_2024_184_MOESM8_ESM.zip › Fig 6/Fig 6G/image/2/7.png]

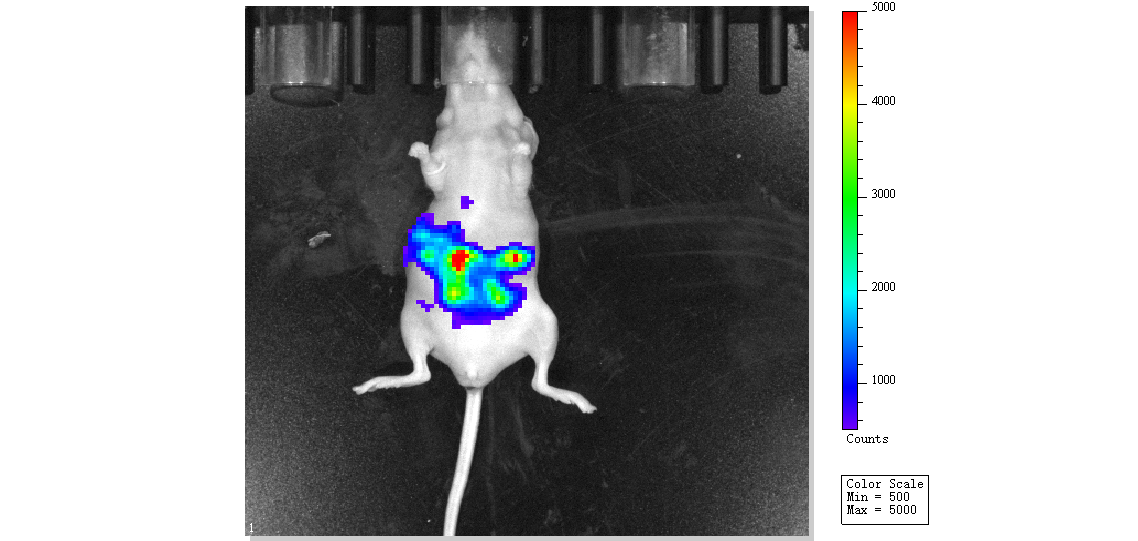

Supplement: Supplementary file 8 — Source data Fig. 6 [file 44321_2024_184_MOESM8_ESM.zip › Fig 6/Fig 6G/image/2/6.png]

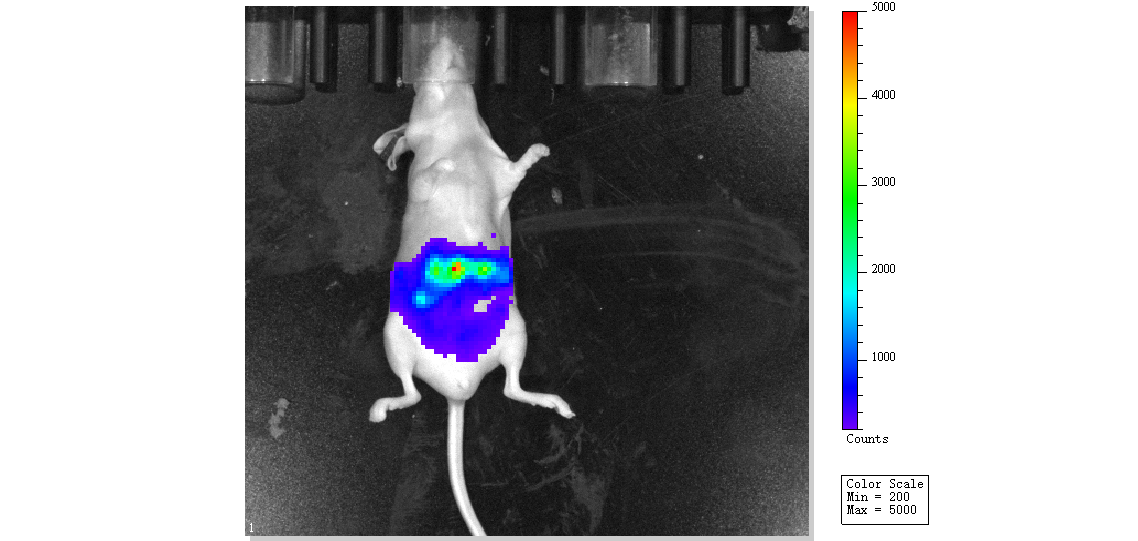

Supplement: Supplementary file 8 — Source data Fig. 6 [file 44321_2024_184_MOESM8_ESM.zip › Fig 6/Fig 6G/image/2/2.png]

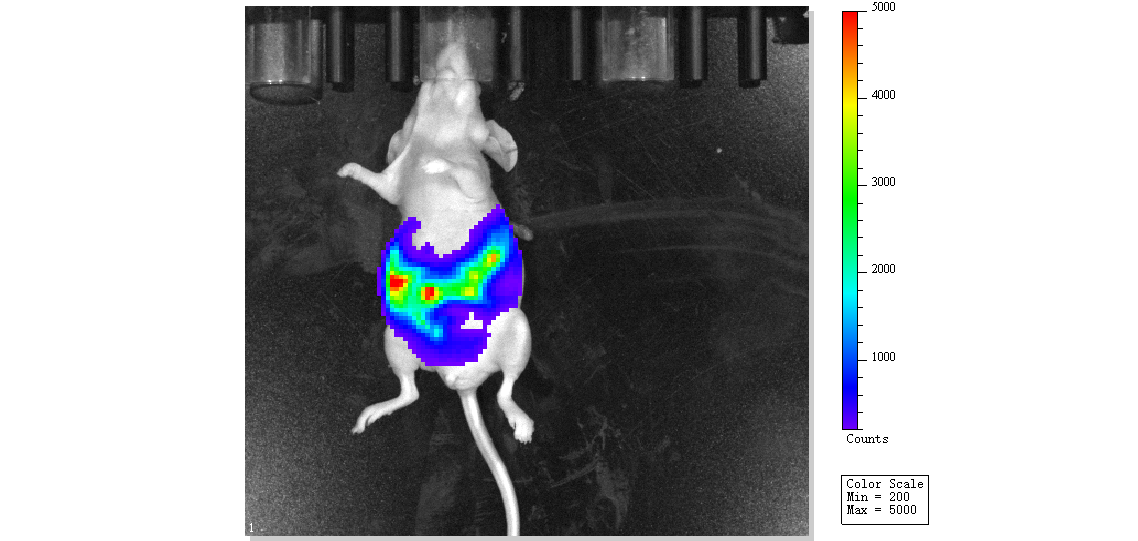

Supplement: Supplementary file 8 — Source data Fig. 6 [file 44321_2024_184_MOESM8_ESM.zip › Fig 6/Fig 6G/image/2/3.png]

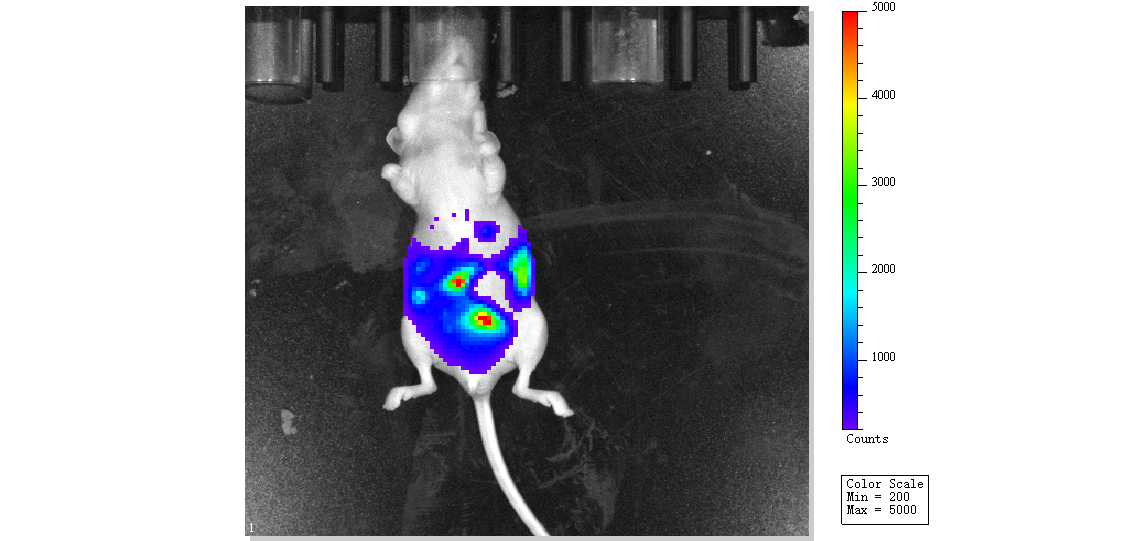

Supplement: Supplementary file 8 — Source data Fig. 6 [file 44321_2024_184_MOESM8_ESM.zip › Fig 6/Fig 6G/image/2/1.png]
